# Molecular and Ionic Diffusion in Ion Exchange Membranes and Biological Systems (Cells and Proteins) Studied by NMR

**DOI:** 10.3390/membranes11060385

**Published:** 2021-05-24

**Authors:** Vitaliy I. Volkov, Alexander V. Chernyak, Irina A. Avilova, Nikita A. Slesarenko, Daria L. Melnikova, Vladimir D. Skirda

**Affiliations:** 1 Institute of Problems of Chemical Physics RAS, 142432 Chernogolovka, Russia; sasha_cherniak@mail.ru (A.V.C.); irkaavka@gmail.com (I.A.A.); wownik007@mail.ru (N.A.S.); 2 Scientific Center in Chernogolovka RAS, 142432 Chernogolovka, Russia; 3 Institute of Physics, KazanFederal University, 420008 Kazan, Russia; melndaria@gmail.com (D.L.M.); kazanvs@mail.ru (V.D.S.)

**Keywords:** ion exchange membranes, hydration, protein association, red blood cells, chemical shift, spin–relaxation, pulsed field gradient NMR

## Abstract

The results of NMR, and especially pulsed field gradient NMR (PFG NMR) investigations, are summarized. Pulsed field gradient NMR technique makes it possible to investigate directly the partial self-diffusion processes in spatial scales from tenth micron to millimeters. Modern NMR spectrometer diffusive units enable to measure self-diffusion coefficients from 10^−13^ m^2^/s to 10^−8^ m^2^/s in different materials on ^1^ H, ^2^ H, ^7^ Li, ^13^ C, ^19^ F, ^23^ Na, ^31^ P, ^133^ Cs nuclei. PFG NMR became the method of choice for reveals of transport mechanism in polymeric electrolytes for lithium batteries and fuel cells. Second wide field of application this technique is the exchange processes and lateral diffusion in biological cells as well as molecular association of proteins. In this case a permeability, cell size, and associate lifetime could be estimated. The authors have presented the review of their research carried out in Karpov Institute of Physical Chemistry, Moscow, Russia; Institute of Problems of Chemical Physics RAS, Chernogolovka, Russia; Kazan Federal University, Kazan, Russia; Korea University, Seoul, South Korea; Yokohama National University, Yokohama, Japan. The results of water molecule and Li^+^, Na^+^, Cs^+^ cation self-diffusion in Nafion membranes and membranes based on sulfonated polystyrene, water (and water soluble) fullerene derivative permeability in RBC, casein molecule association have being discussed.

## 1. Cation-Exchange Membranes. Structure, Hydration, Ionic, and Molecular Mobility

Ion-exchange membranes are widely applied for modern electrochemical technologies and separation processes. New materials design requires an electro mass transfer investigation. This research is mainly concerned about macroscopic transport processes [1,2,3,4,5,6,7]. However, ion and molecular translation microscopic mobilities have to be investigated for membrane selectivity mechanism understanding.

Of most interest is the relationship between the following fundamentally important characteristics that determine the ion and molecular transport:

1. The nanoscale structure of ion transport channels. The structure and dynamics of polymer matrix at the submicro level from several tenths of nanometer (sizes of solvated ions and molecules) to several nanometers or several tens of nanometers (characteristic lateral dimensions and lengths of ionic channels), determine the selective ion transport because these structural units form transport path for ion transfer by macroscopic distances. Studying the nanostructure opens up the prospects for targeted synthesis of ion exchange polymer, insofar as their preparation is accompanied by the formation of the nanostructure.
2. The type of interaction of mobile ions and hydration water molecules with functional groups. Data on the structure of ionic complexes and on the mechanisms of interaction of ions and water molecules with the polymer matrix are necessary for understanding the mechanisms of selectivity of ion-exchange membranes and elementary steps of the diffusion transport of ions.
3. The elementary steps of diffusion of ions and molecules, which can be characterized by the lifetime of a species on functional group, the time of translational displacement, the partial diffusion coefficient on various spatial scales (if diffusion occurs in a heterogeneous medium).

The problem of elementary diffusion jumps logically follows from the aforesaid. Evidently, the time of elementary jump and the height of the potential barrier overcome by a moving species are largely determined by the geometry of diffusion channels and the structure of hydrate ionic complexes. This information is necessary for both the elaboration of adequate transport models and the targeted synthesis of high-performance ion exchange polymers.

The knowledge of structure and dynamics in a different spatial scale and in a broad band of molecular motion frequencies may be obtained by NMR directly.

*NMR spectroscopy* The most popular method is ^1^H NMR, which was used to study Dowex 50 W, CU-2 sulfonate cation exchanger resin and the corresponding membranes MC-40, cation exchange membranes based on polyethylene and sulfonated grafted polystyrene MSC [8,9,10,11,12,13,14,15,16,17] and perfluorinated cation-exchange membranes [18,19,20,21,22,23,24,25,26]. To date, techniques have been developed for recording high-resolution NMR spectra and the main factors that determine the chemical shift of water protons in granulated sulfonate cation-exchangers and ion-exchange membranes have been elucidated. The required information can also be obtained from the solid-state high-resolution NMR spectroscopy data [23,24]. Information on hydration of ionic channels in membranes is of fundamental importance for understanding the mechanism of migration of cations and water molecules.

Alkaline metal cations of lithium, sodium, and cesium were studied by NMR on ^7^ Li, ^23^ Na and ^133^ Cs nuclei in cation-exchange membranes [16,17,20,21,27,28,29,30,31,32] and in sulfonated polystyrene salts [33,34]. Some qualitative data about ionogenic group-cation interaction and cation motion were obtained.

*Pulse NMR methods* NMR relaxation techniques were for the first time applied for local cationic and water molecules mobility characterization in polymeric electrolytes more than 50 years ago [10]. Spin–lattice and spin–spin relaxation times measurements on ^1^ H, ^7^ Li, ^19^ F NMR nuclei were performed in Nafion and MF-4SC (Russian Nafion type membrane) membranes [35,36,37,38,39,40,41]. Unfortunately, the numerical calculation of correlation times is hard work because of wide molecular motion frequency distribution.

The study of the metal ion mobility is associated with even more serious complications. The most serious obstacle is the absence of theoretical works to serve as the base for studying the region of diffusion motion with the characteristic correlation times longer than ω_o_^−1^ (where *ω*_0_ is the NMR frequency, usually, ~10^9^ Hz). To date, ^7^ Li and ^23^ Na NMR relaxation works have been performed dealing with the mobilities of lithium and sodium cations in the CU-2 type sulfonate cation exchangers and the corresponding membranes, perfluorinated sulfonate cation-exchange membranes [31,32,33,34,39,40,41].

The pulsed field gradient NMR method, [42,43] which makes it possible to directly measure the diffusion coefficients of protons and other ions in heterogeneous media, is free from these drawbacks. The number of studies of this type substantially increased in recent decades, [5,6,19,38,39,40,41,44,45,46,47,48,49,50,51,52,53,54,55,56,57,58,59,60,61,62,63,64,65,66,67,68,69,70,71,72,73,74,75,76,77,78,79,80,81,82,83] which was associated with the increased interest in the problems of ionic mobility in polyelectrolytes.

To summarize the foregoing, the following points should be underlined. The magnetic resonance techniques and, especially, NMR methods provide the unique possibility of acquiring detailed information on the state of molecules and ions, the local molecular and ionic mobility, and the diffusion on the spatial scale from several tenths of nanometer to several millimeters. The advantages of NMR spectroscopy also involve the possibility of studying one and the same sample under conditions resembling the service conditions by several methods simultaneously, which makes it possible to compare the results of different measurements and unambiguously interpret them. For these studies to be performed the experimental procedures should be worked out and the problems associated with theoretical quantitative description of data should be solved.

The NMR methods are especially attractive for acquiring detailed information on the ion and molecular transport in polymer electrolytes. The modern level of experimental research instruments allows one to study both elementary processes and macroscopic transfer under the service conditions of electrochemical systems. The successful introduction of NMR methods into the research and technological practice is limited by the lack of publications devoted, first of all, to demonstration of the potential of experimental NMR techniques in this research field.

Despite the considerable number of NMR studies of polymer electrolytes, the reviews on this subject are scarce. In the present review, the experimental results obtained by NMR methods on the ion and water molecular transport in polymer ion exchangers carried out in Russia and abroad are analyzed and generalized. From our point of view, such an analysis will demonstrate the potential of modern NMR methods and help to reveal some fundamental features of ion and molecular transport in ion exchange membranes at the molecular level.

The main results of NMR studies in ion-exchange membranes are discussed. Attention is focused on the potential of NMR techniques in solving particular problems in relation to the most widely known ion exchangers. The most thorough studies were carried out for perfluorinated membranes. Using these membranes as examples, an attempt is made to find the relationship between the polymer matrix structure, the ion hydration details, and the diffusion mobility of ions and molecules on different spatial scales and then to apply this information for revealing details of the ion transport mechanism in ion-exchange membranes.

### 1.1. Ion-Exchange Membranes, Nanochannel Structure

In this review we consider mainly two types of well-known cation exchange membranes.

The first type is perfluorinated sulfonic cation exchange membrane Nafion and its Russian analog MF-4SC, carboxyl cation exchange membrane F-4CF. The second one is membranes on the basis of sulfonated polystyrenes.

**Figure 1:**
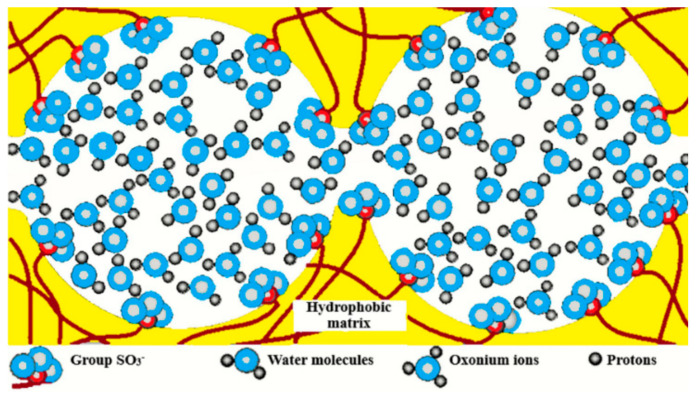
Schematic representation of Gierke model. Reprinted with permission from [87]. Copyright 2013 Springer Nature.

The main studies with the use of NMR methods were carried out on perfluorinated sulfonate cation-exchange membranes Nafion and MF-4SC (Russian analogue of Nafion). At present, the nanostructure of perfluorinated sulfonate cation-exchange membranes has been studied in sufficient detail. Several models were proposed for describing the transport channels [5,84,85,86,87,88,89,90,91,92,93,94,95] of which the Gierke model [5,6,89,90,91] was used most widely (Figure 1). This model is also applied for describing of inorganic channels in polystyrene sulfonic cation exchange resins and membranes.

**Figure 2:**
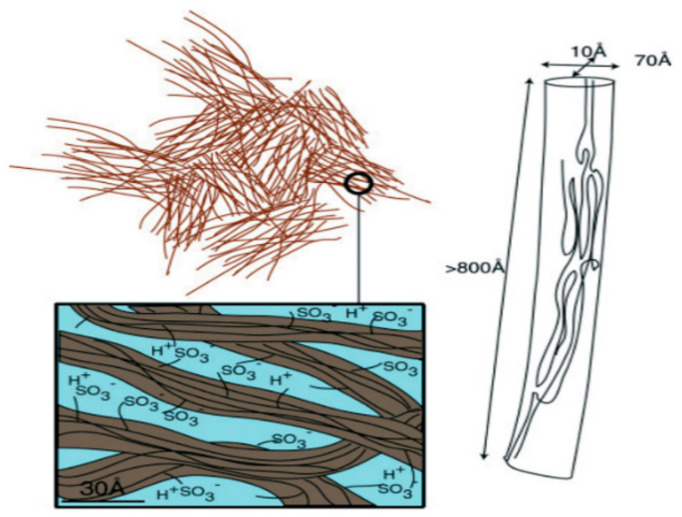
New insight of a Nafion membrane multiscale structure (adapted from [95]).

This model is based on the concept that water molecules and counterions form spherical clusters with walls built of sulfo groups. It was proposed that the clusters are connected by channels. The limiting step of diffusion is ion and molecular transport in the channels, which were not observed experimentally. On the basis of more detailed small angle X-ray scattering investigation, a group of authors [5,84,85,95] proposed the channel model of perfluorinated membranes (Figure 1 and Figure 2).

**Figure 3:**
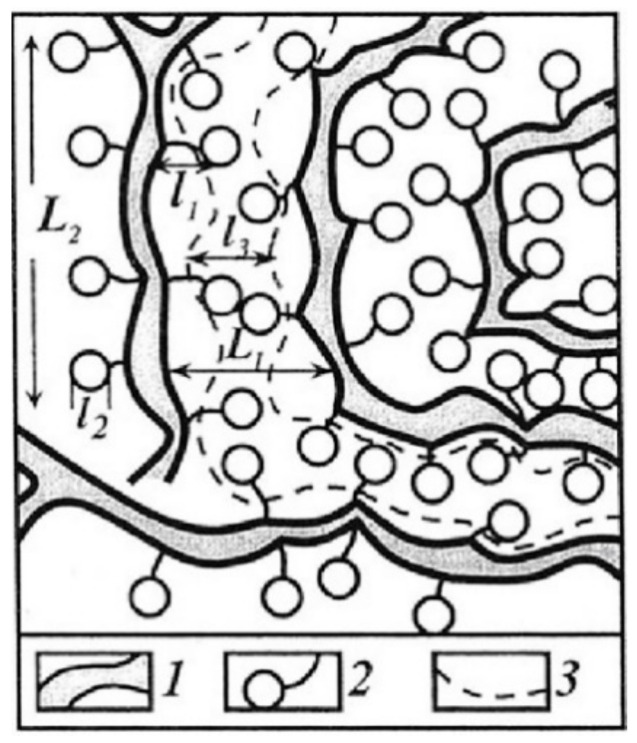
Structure of the amorphous part of a perfluorinated sulfonate cation-exchange membrane [5]. (**1**) Polymer backbone; (**2**) hydrated counter-ions and functional groups at a low moisture content; (**3**) transport channels for ions and water molecules at a high moisture content; *L*_1_ = 4 nm according to small-angle X-Ray scattering data [84,85]; *L*_2_ = 10 nm according to Mössbauer spectroscopy [93]; *l*_1_ = l.2–1 nm according to ENDOR and relaxation NMR data [96,97,98,99]; *l*_3_ = 1.5 nm according to standard porosimetry [94] and ENDOR [98,99] methods.

The detailed Nafion structure model is based on the data of small-angle X-Ray scattering [84,85] Mössbauer spectroscopy [93] and standard porosimetry [94] and shown in Figure 3. It was found that associates including sulfo groups, counterions, and water molecules are formed in the amorphous part of the membrane. These associates form the ion transport channels with the volume of approximately a fourth of the total membrane volume [96,97].

The key point of this structure model is the comparison of macroscopic lithium cations and water molecules self-diffusion coefficients with calculated microscopic self-diffusion coefficients (Table 1). From NMR relaxation data the correlation times *τ*_i_ of water molecules and Li^+^ cations jumping were calculated. Self-diffusion coefficients of water molecules and lithium cations were estimated from Einstein equation as *l*^2^/6*τ*_i_ where *l* is the jump length of water molecule (0.3 nm) or Li^+^ cation (0.7 nm, the average distance between SO_3_^−^ groups).

The good agreement between experimental macroscopic and calculated self-diffusion coefficients is observed. It is confirmed the conclusion from channel structure model that macroscopic water and ion transfer is controlled by microscopic particle jumping.

Therefore, this interpretation of ^1^ H and ^7^ Li relaxation data is conformed to Nafion structure model (Figure 3).

Another extensive class of membranes is sulfonate cation-exchange membranes based on styrene-divinylbenzene. Both heterogeneous and homogeneous membranes were studied as well as gel and macroporous sulfonate cation exchangers CU-2 and CU-23 and their foreign analogues. The ESR studies of Cu^2+^ cations used as paramagnetic probes revealed two domains containing hydrated copper (II) complexes with different mobility [100]. The dependences of the correlation times and diffusion coefficients of water on the moisture content [44,45] were consistent with the assumption that the structure of transport channels in such ion exchangers is described by the model in Figure 1, which is widely used for ionic transport in these ion-exchangers. Cluster-channel structure model is also suitable for explanation of electro mass transfer in membranes (MSC) based on polyethylene and sulfonated grafted polystyrene [16,17].

It should be concluded that ion transport is related in many respects to the mobility of water molecules and is determined by the character of membrane hydration. To reveal the mechanisms of ionic conduction, it is important to correlate the character of hydration of ionogenic groups with the translational mobility of water molecules and ions and the ionic conductivity of membranes. Such studies should be carried out for membranes with a well-known structure of transport channels.

### 1.2. Specific Features of Cation Hydration. Mechanism of Cation—Functional Group Interaction

For a cation hydration characterization in cation-exchangers, ^1^H NMR spectra of water molecules are analyzed. The first calculation of one charge cation hydration numbers were carried out on sulfonate cation exchange resin Dowex 50 W in the beginning of 1970-th just after spectrometers of NMR started to be on sale.

#### 1.2.1. Cation Hydration

##### The Dependence of 1H Chemical Shift on the Humidity

Acid ionic form

The most thoroughly studied membranes are perfluorinated sulfonate cation-exchange membranes Nafion, MF-4SC [18,19,20,21,22,23,25], MF-4SC membranes modified with inorganic dopants [21] and perfluorinated carboxylic membranes F-4CF in various ionic forms [20,21]. 
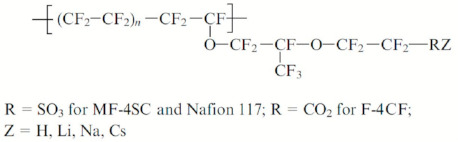


**Figure 4:**
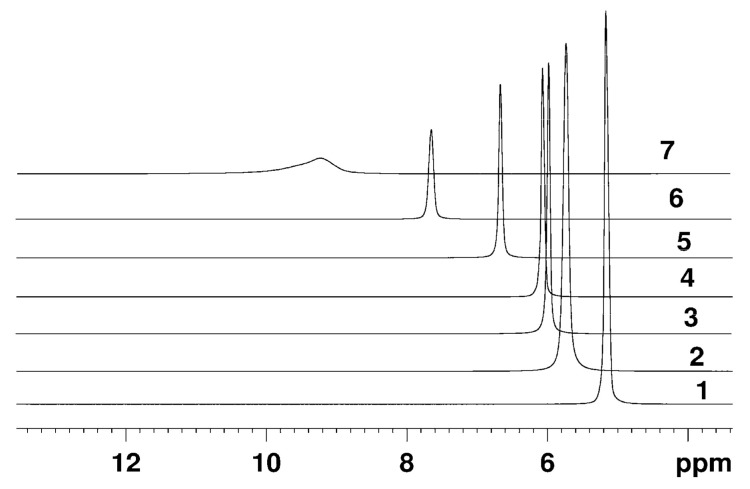
^1^ H NMR spectra in acid ionic form of Nafion 117 membrane at different humidity: (1)*RH* = 95%; (2) *RH* = 78%; (3) *RH* = 64%; (4) *RH* = 58%; (5) *RH* = 32%; (6) *RH* = 10%; (7) *RH* = 0%. Reprinted with permission from [25]. Copyright 2019 Springer Nature.

**Figure 5:**
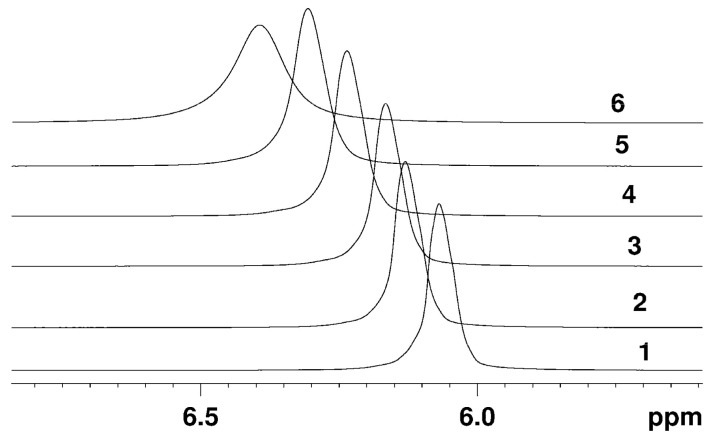
^1^ H NMR spectra in acid ionic form of Nafion membranes at different temperatures. Membrane samples were equilibrated with water vapor at 58% relative humidity. (1) +25 °C; (2) +10 °C; (3)0 °C; (4) −20 °C; (5) −40 °C; (6) −60 °C. Reprinted with permission from [25]. Copyright 2019 Springer Nature.

The typical NMR spectra of protons in a perfluorinated sulfonate cation exchange membrane in its Li^+^, Na^+^, Cs^+^ forms and of hydrated H^+^ cations measured at various relative humidity of the environment are rather narrow singlet lines, indicating high mobility of water molecules and H^+^ counter-ions in membranes. Some examples of the evolution of ^1^H NMR spectra at different humidity and temperature are shown in Figure 4 and Figure 5.

The H^+^ ionic form ^1^H NMR spectrum is a singlet, which position is shifted to lower magnetic fields relatively to bulk water signal. The NMR line is rather narrow even at low humidity and below 0 °C which indicates high proton mobility at these conditions. The chemical shift value depends on water content and temperature (Figure 4 and Figure 5).

**Figure 6:**
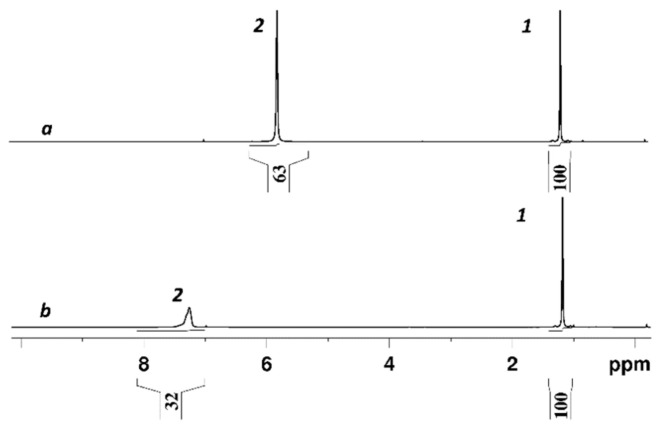
The example of ^1^H NMR spectra of Nafion 117 membrane in 95 volume percent CCl_4_ + 5 volume percent of C_6_H_12_: (**a**) Nafion 117 membrane equilibrated at 75% *RH*, (**b**) Nafion 117 membrane equilibrated at 10% *RH*; 1–signal of C_6_H_12_, 2–signal of water and hydrated H^+^ protons. Figures under signals are the area of NMR lines. Reprinted with permission from [25]. Copyright 2019 Springer Nature.

Counter ion H^+^ hydration numbers calculation in sulfonate cation exchange resins and membranes [8,9,13,14,15,16,25], as well as in aqueous solutions of sulfonic acids [101,102,103,104] on the basis of ^1^H chemical shift temperature and moisture content dependences analysis, was carried out. The absolute value of water molecules per sulfonate group (*λ*) have to be obtained in order to calculate hydration number correctly. As a rule membrane moisture content (*λ_g_*) is determined by gravimetric technique. In our case during this procedure the sample of membrane dries on phosphoric anhydride or at temperature 110 °C until the constant mass. After this drying ^1^H NMR line remains narrow (spectrum 7 in Figure 4) which is evidence of high proton mobility. Therefore it was assumed that in perfluorinated sulfonate cation membranes two residual water molecules per H^+^ cation are persisted and form hydroxonium ion [H_5_O_2_]^+^ [13,14,15,18,19,25,105]. The absolute water content was determined by ^1^ H NMR spectroscopy technique directly and residual water content is 1.50 ± 0.50 in Nafion 115 membrane after drying to constant weight at 100 °C [106]. Following this paper, we have measured the absolute water content in Nafion 117 membrane dried to the constant weight at 110 °C or equilibrated with P_2_O_5_ at room temperature. ^1^ H NMR spectra of the membrane at 10% and 75% humidity are shown in Figure 6. Cyclohexane solution in carbon tetrachloride inserted in the NMR sample tube was used as standard (line 1 in Figure 6). Line 2 in the Figure 6 is the averaged signal of water molecule protons and hydrated H^+^ cation.

The amount of residual water molecules, calculated as the average value from 20 measurements for different drying procedures (drying at 110 °C and with P_2_O_5_) was *λ*_0_ = 1.9 ± 0.4.

The observed ^1^H NMR line is the average signal from H^+^, hydrated H^+^(H_2_O)_h_ water molecules, and water molecules of the next hydration spheres. Due to the fast (compared to the chemical shift difference between these three proton positions) molecular exchange the only NMR line with an average chemical shift is observed as it is shown in Figure 4 and Figure 5. The resulting chemical shift *δ* is a superposition of chemical shifts of H^+^ and hydrated water molecules *δ_c_* in H^+^(H_2_O)_h_ and water protons *δ_H2O_*—Equation (1), where *p_c_* and *p_H2O_* are the respective relative protons fractions.

(1) $$\delta =p_{c}\cdot \delta_{c}+p_{H_{2}O}\cdot \delta_{H_{2}O}$$

It is assumed that H^+^ ion forms stable aqua complex with humidity independent hydration number *h*. The chemical shift *(δ-δ_H2O_)* is approximated by Equation (2).

(2) $$\delta -\delta_{H_{2}O}=\frac{\left(2h+1\right)\cdot \left(\delta_{c}-\delta_{H_{2}O}\right)}{2\left(\lambda_{g}+h\right)+1}$$

**Figure 7:**
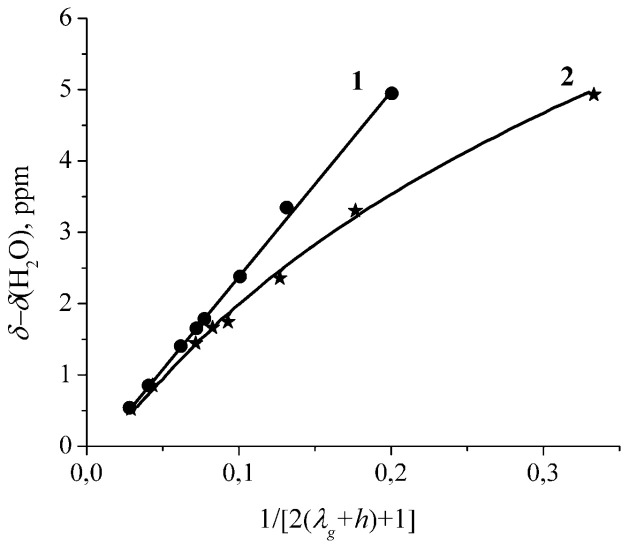
Dependences on humidity of ^1^H chemical shift (relative to the bulk water signal) in Nafion 117 membranes. *λ_g_* is the gravimetrically measured amount of water molecules per sulfonate group after membrane drying to constant weight at 110 °C or equilibrated with dry P_2_O_5_, *h* is hydration number of H^+^(H_2_O)_h_); 1—*h* = 2 (H_5_O_2_^+^) line is Equation 1.2 at *δ_H2O_ =* 4.3 ppm and *δ_c_* = 13.8 ppm, circles—experiment; 2—*h* = 1 (H_3_O^+^) is experimental curve given for comparison. Reprinted with permission from [25]. Copyright 2019 Springer Nature.

The dependences of chemical shift on the humidity are shown in Figure 7. This dependence is described well by Equation (2) when *h* is 2 (curve 1, Figure 7). This *h* value is equal to the residual water amount *λ*_0_ *=* 1.9 ± 0.4 which was obtained above by the direct NMR measurement. Therefore, it may be concluded that after membrane drying until the constant weight two hydrated water molecules form hydroxonium cation H_5_O_2_^+^. This conclusion is agreed with sulfonic cation exchanger resins and MF-4SC membrane H^+^ ionic form ^1^H NMR investigation [8,9,13,14,15,16].

Some authors [107,108] have proposed that in dry Nafion H^+^ ionic forms the hydronium ion H_3_O^+^ (*h* = 1) is formed. According to the direct NMR measurements carried by us and the authors of [106] the amount of residual water *λ*_0_ is about two and the dependence of chemical shift on humidity is approximated by Equation 1.2 at *h* = 2 (curve 1 in Figure 7) that corresponds to hydroxonium ion. Assumption of one residual water molecule (hydronium ion, *h* = 1) contradicts to the direct water content NMR measurements and Equation (2) (curve 2, Figure 7). Therefore, the assumption of hydroxonium ion formation at low water content looks more probable.

Two residual water molecules and (H_5_O_2_)^+^ hydrated ion formation at low water content are typical for all sulfonic cation exchangers: sulfonic cation exchange resins, MC, and MSC membranes.

Salt ionic forms

**Figure 8:**
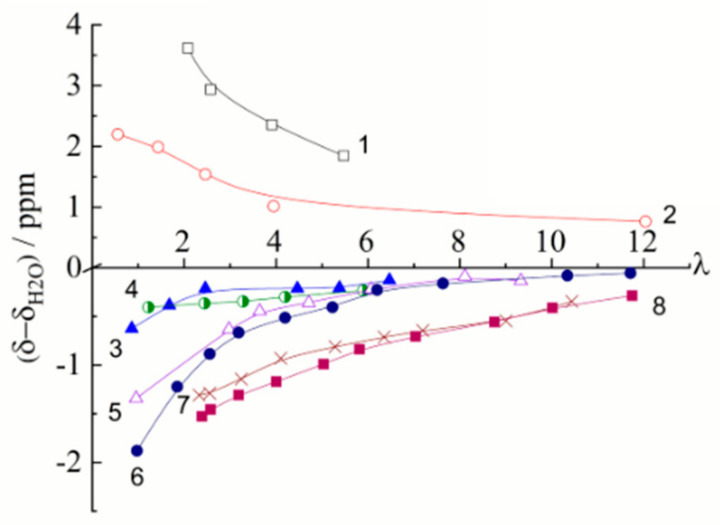
Chemical shift (*δ*) differences between water protons in the membrane and in bulk water as a function of the moisture content of MF-4SC (1–4) and F-4CF (5–8) membranes in various ionic forms. Membrane ionic form: (1, 5) H^+^, (2, 6) Li^+^, (3, 7) Na^+^, (4, 8) Cs^+^. Reprinted with permission from [20]. Copyright 2011 Elsevier.

**Figure 9:**
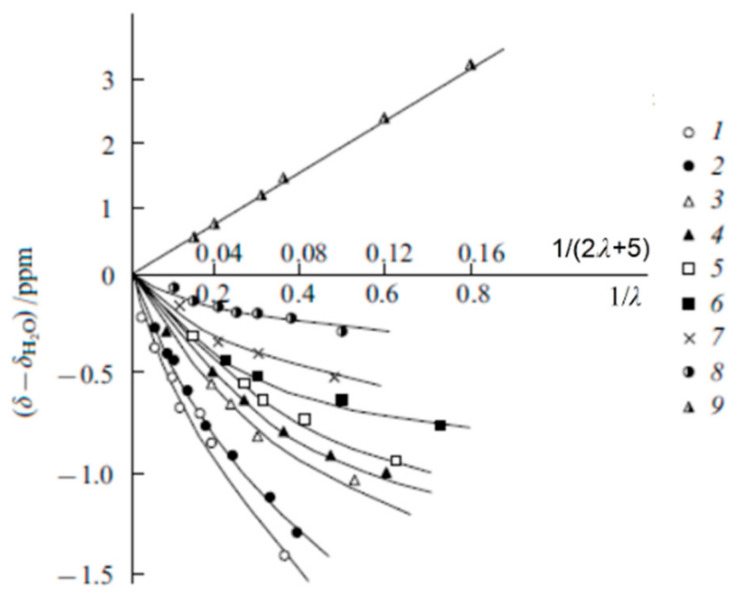
Dependences of chemical shifts of water on the moisture content of MF-4SC membrane. Membrane ionic form: (1) Li^+^, (2) Na^+^, (3) K^+^, (4) Rb^+^, (5) Cs^+^, (6) Ba^2+^, (7) Ca^2+^, (8) Mg^2+^, (9) H^+^ (adapted from [19]).

As the moisture content decreases, the NMR spectral lines shift downfield for membranes in the acidic form and upfield for membranes in the salt form relative to those of free water. Such changes in the chemical shifts of water protons cause by a destruction of a hydrogen-bond network in the salt-form membranes and strengthening of hydrogen bonds as a result of hydration of H^+^ counter-ions. Dependences of ^1^H chemical shift on water content in different ionic forms of sulfonated and carboxylic membranes are shown in Figure 8 and Figure 9.

The moisture content measured by gravimetry is expressed as the number of water molecules (*λ*) per ionogenic group. The ^1^ H chemical shifts sharply change starting from a certain critical value *λ* because at low water content, all water molecules occupy the first hydration shell of the cation and are strongly affected by the cation. The hydration energy of H^+^, Li^+^, and Na^+^ cations is higher and the hydration energy of Cs^+^ is lower than the hydrogen bond energy; hence, the changes in the chemical shifts of water protons are not so pronounced in Cs films (curve 4, Figure 8 and curve 8, Figure 9). In the acidic form of sulfonate cation exchange membranes two remaining water molecules forms the stable cation H_5_O_2_^+^ with the counter-ion H^+^. Therefore the observed dependence of the chemical shift on the real fraction of water protons in the membrane 1/(2*λ* + 5) is a straight line (line 9 in Figure 9) [19].

The calculated hydration numbers (the average number of water molecules in the hydration shell of an ion (*h*)) for H^+^, Li^+^, Na^+^ and Cs^+^ cations are as follows [19,20], Table 2:

The hydration numbers for Li^+^, Na^+^ and Cs^+^ were also assessed from the dependences of ^7^ Li, ^23^ Na, and ^133^ Cs chemical shifts on the moisture content. These values agree well with the data of proton magnetic resonance.

The hydration numbers of cations in carboxylic membranes are smaller than in sulfonate cation exchange membranes. This is probably caused by the fact that in F-4SC membranes, the hydration shell of cations includes, first of all, carboxyl groups, whereas in MF-4SC membranes even at *λ* ~ *h* the local environment of a cation contains only water molecules.

It should be noted that the hydration numbers of doubly charged ions substantially exceed those of singly charged ions [19]. In the membranes with a different polymer matrix and, correspondingly, a different structure of transport channels, for instance, styrene–divinylbenzene systems, the hydration is of the same nature.

**Figure 10:**
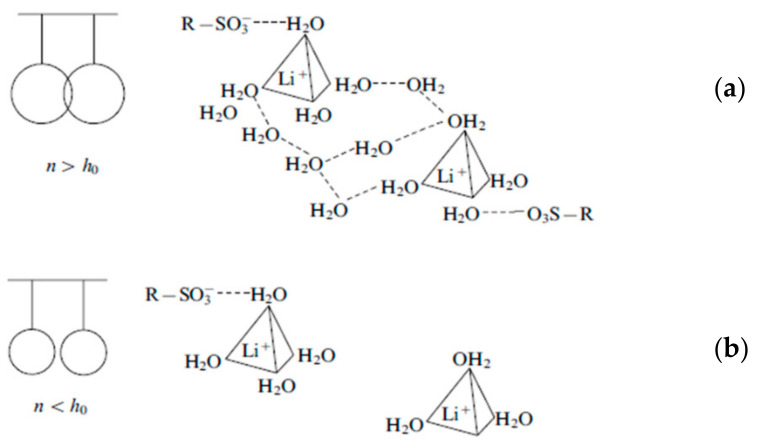
Illustration of hydration of functional groups in the salt forms of MF-4SC membranes by the example of its Li^+^-form. Reprinted with permission from [39]. Copyright 2002 Springer Nature.

This fact and the weak dependence of the chemical shift in electrolyte solutions on the type of anion suggest that in cation-exchange membranes, water molecules interact mainly with cations. The fraction of broken hydrogen bonds increases with increase in the hydration energy of cations [29]. For *λ* > *h*, the cation and the functional group form a solvent-separated ion pair and the hydration shells of ionogenic groups overlap (Figure 10a). For a low moisture content *λ* < *h,* a network of hydrogen bonds between water molecules is broken, thus hampering the ion jumping between the neighboring ionogenic groups (Figure 10b).

##### Temperature Dependence of ^1^H Chemical Shift

Acid ionic form

**Figure 11:**
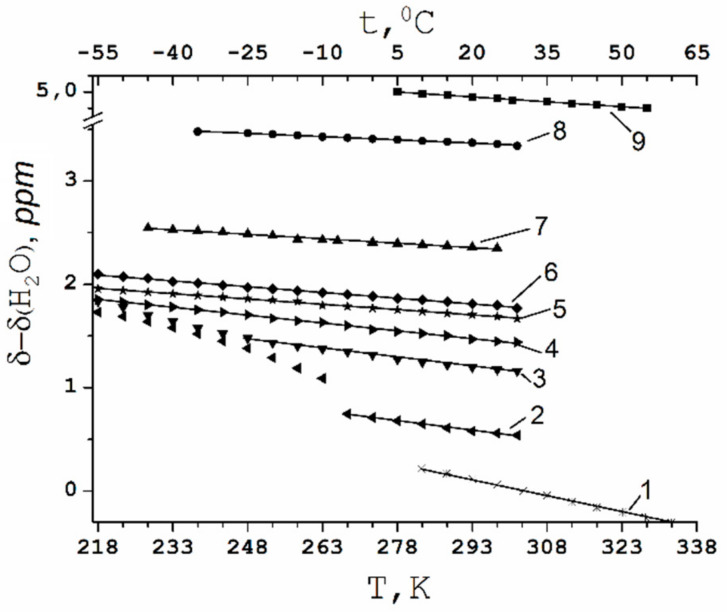
Temperature dependences of ^1^ H NMR chemical shifts in acid ionic form of Nafion membrane at different water content (*δ_H2O_* is bulk water chemical shift at 25 °C). (1) bulk H_2_O; (2) *RH* 98% (*λ* = 17.5 ± 0.4); (3) *RH* 95% (*λ* = 12 ± 0.4); (4) *RH* 78% (*λ* = 7.4 ± 0.4); (5) *RH* 64% (*λ* = 6.4 ± 0.4); (6) *RH* 58% (*λ* = 5.8 ± 0.4); (7) *RH* 32% (*λ* = 4.4 ± 0.4); (8) RH 10% (*λ* = 3.2 ± 0.4); (9) *RH* 0% (*λ* = 1.9 ± 0.4, drying on P_2_O_5_ or at 110 °C). Reprinted with permission from [25]. Copyright 2019 Springer Nature.

Counter ion H^+^ hydration number may be also calculated from ^1^ H chemical shift temperature variation. The ^1^ H NMR spectra in Nafion 117 membrane at different temperature is shown in Figure 5. In acid ionic form of membrane lines are shifted to lower magnetic fields, but line width increases with the lowering temperature. The narrow line width at temperature below 0 °C indicates high proton mobility at low temperature. The temperature dependences of chemical shift at different water contents are shown in Figure 11. These dependences are straight lines until *λ* = 7–8 in the whole temperature range from −60 °C to + 50 °C (curves 4-9 in Figure 11) and at *λ* = 12 in temperature range above −25 °C (curve 3 in Figure 11) and at *λ* = 17.5 above −10 °C (curve 2 in Figure 11). The slope of these lines increases with the increasing of *λ*; the slope of the lines is highest for bulk water (curve 1 in Figure 11).

Follow to ^1^ H NMR temperature investigation of acidic aqueous solutions [101,102,103] and sulfonic cation exchange resins [8,9,14,15,16] in H^+^ ionic form the hydration numbers were calculated from Equation (3):

(3) $$h=\lambda -\frac{\left(0.5+\lambda \right)\cdot \frac{d\delta }{\mathrm{dT}}}{\frac{{d\delta }_{H_{2}O}}{\mathrm{dT}}},$$

where *λ* = λ_g_ + *λ*_0_. Hydration numbers *h* of H^+^ cation in H^+^ ionic form Nafion membranes at different *λ* are listed in Table 3.

Hydration numbers are differing with humidity variation. At low water content (*λ* is about 2–4) *h* is closed to 2, which agrees with previous result, obtained from proton chemical shift humidity dependences. With following *λ* increasing *h* also increases until *h* ≈ 4.

Hereby in spite of rather crude approaches of proton chemical shift humidity and temperature dependences interpretation some important conclusions may be done.

At low water content H^+^ cation strongly binds two water molecules which are not able to be desorbed at high temperature and vacuum drying forming hydroxonium H_5_O_2_^+^ ion. With humidity increasing the more water rich hydrated complex H_9_O_4_^+^ is formed. The same hydration peculiarities are observed for all sulfonic cation exchangers.

Salt ionic forms

From ^1^ H chemical shift temperature dependences hydration numbers of Li^+^, Na^+^, and Cs^+^ ions may be calculated. Relevant techniques were applied to sulfonic cation exchangers [8,9,14,15,16] and salt aqueous solutions [101,102,103].

**Figure 12:**
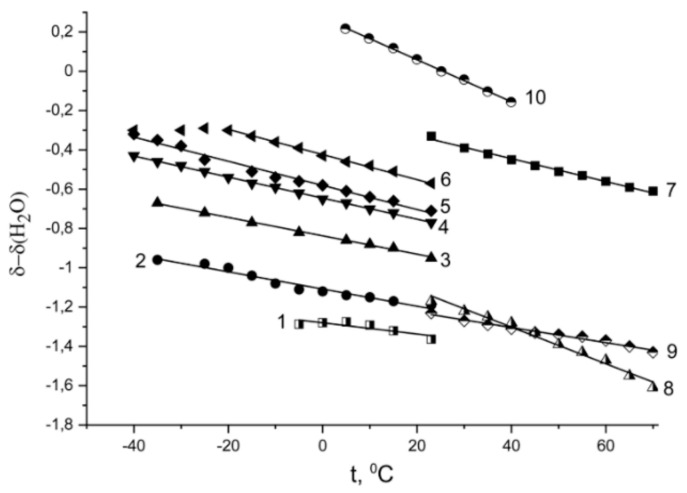
Temperature dependence of water proton chemical shifts in the Li^+^, Na^+^, and Cs^+^ ionic forms of Nafion membranes at various relative humidity where *δ_H2O_* is the bulk water chemical shift *δ_H2O_* = 4.30 ppm relatively TMS, at 20 °C: Li^+^ ionic form: (1) *λ* = 0.9, (2) *λ* = 2.0, (3) *λ* = 4.0, (4) *λ* = 5.7, (5) *λ* = 7.4, (6) *λ* = 10.7, (7) *λ* = 12; (8) Na^+^ ionic form, *λ* = 10; (9) Cs^+^ ionic form, *λ* = 4; (10) bulk water; λ is amount of water molecules per sulfonated group. Reprinted with permission from [109]. Copyright 2021 Elsevier.

Temperature chemical shift dependences at different water contents are shown in Figure 12. These dependences are straight lines, which slope increased following by *λ* increasing; the line slope is highest for bulk water (curve 10 in Figure 12).

Following ^1^ H NMR temperature investigation of sulfonic cation exchange resins and membranes [8,9,14,15,16] as well as salt aqueous solutions [101,102,103] in salt ionic form the hydration numbers *h* were calculated from Equation (4):

(4) $$h=\lambda \left[1-\frac{\frac{d\delta }{\mathrm{dT}}}{\frac{{d\delta }_{H_{2}O}}{\mathrm{dT}}}\right],$$

where *λ* is the number of water molecules per sulfonate group; *δ* is the measured ^1^H chemical shift; *δ_H2O_* is the bulk water ^1^ H chemical shift. Hydration numbers *h* of Li^+^ cation in appropriate ionic form of Nafion membranes at different humidity are listed in Table 4.

Hydration numbers are differed with humidity variation. At high water content *λ* > 10.7, hydration number *h* about 4–6, this value is closed to lithium cation hydration number in dilute lithium salt aqueous solutions (*h* = 5–6), where Li^+^ forms a separate ionic pair with anion. With reducing water content Li^+^ hydration number is decreased and oxygen atom of sulfonate group replaces the oxygen of water molecule; thereby a contact ionic pair is created. Sodium cation hydration number *h* is 6 ± 1 (*λ* = 10 at 98% *RH*) which is the same as *h* in dilute sodium salt aqueous solutions [103,104] so the separate ionic pair Na^+^—SO_3_^−^ group is formed at these conditions. For Cs^+^ cation hydration number *h* is 1 ± 0.2 (*λ* = 4 at 98% *RH*) this value is less compare with *h* in aqueous solution [103] and in Dowex 50W (cation resin) [8] (*h* = 3–4). It means that even at maximum water content Cs^+^ interact with Nafion SO_3_^−^ group directly and contact ionic pair is forming. This cesium cation hydration particularity is due to the fact that compare to lithium and sodium cations Cs^+^ hydration energy is less than hydration bond energy between water molecules [110].

Hereby, in spite of rather crude approaches of proton chemical shift temperature dependences interpretation, some important conclusions may be done.

At low water content Li^+^ and Na^+^ cations are in direct contact with sulfonate groups, while at high water content water molecules are built in between cation and SO_3_^−^ groups. Cesium cation interacts with sulfonate group directly at any water content.

Hydration numbers *h* of Li^+^, Na^+^ Cs^+^ in MSC membrane are shown in Table 5, hydration numbers for same cations in equimolar aqueous salt chloride solutions and membrane water uptakes are also shown for comparison. The *h* values of Li^+^, Na^+^, and Cs^+^ in MSC membranes at high humidity is practically equal to salt solution ones. The crystallography radii and Stokes–Einstein hydrodynamic ion radii are also presented in Table 5. We have calculated hydrodynamic ion radii from Stokes–Einstein equation on the basis of ionic diffusion coefficient in chloride aqueous solution. Ion diffusion coefficient concentration dependences were approximated to infinite dilute concentration.

**Figure 13:**
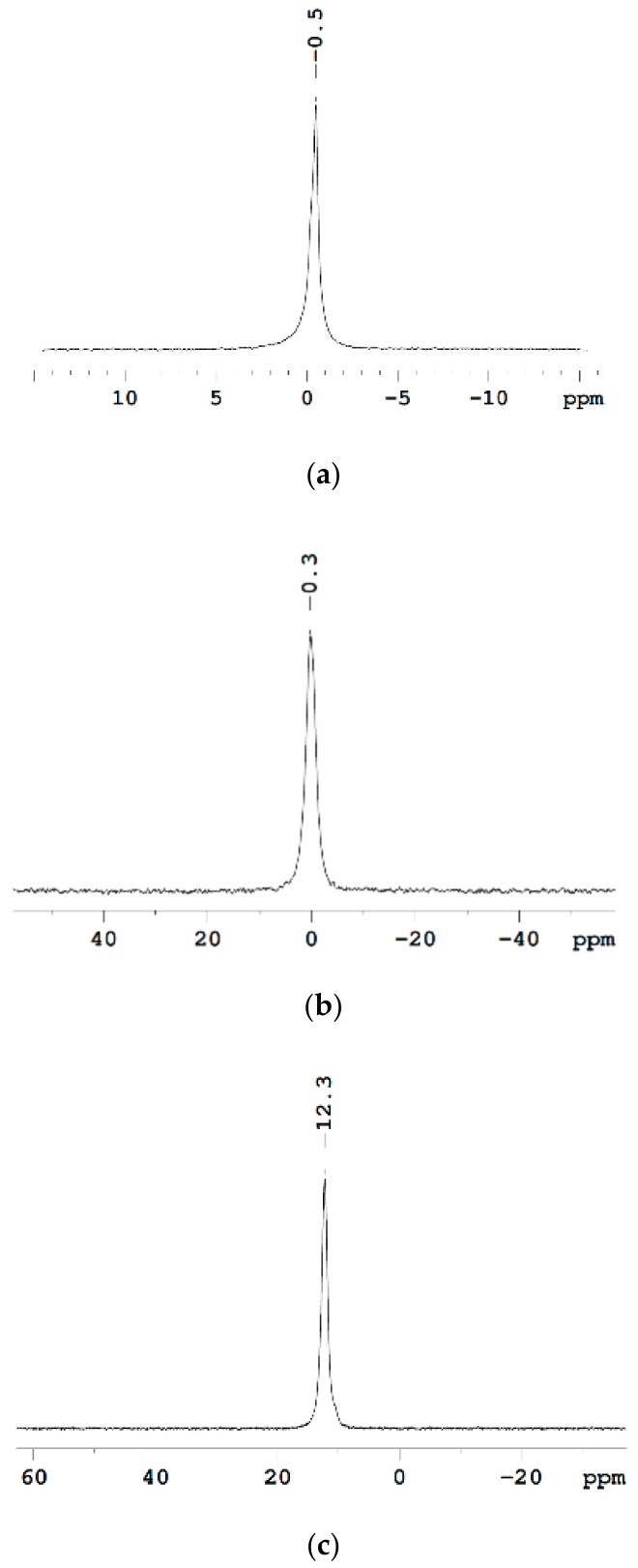
NMR spectra of ^7^ Li (**a**), ^2 3^Na (**b**), and ^133^ Cs (**c**) nuclei in appropriate ionic form of MSC membrane at *RH* = 95% [17].

Li, ^23^ Na, and ^133^ Cs NMR Spectroscopy. Mechanisms of Cation—Ionogenic Group Interaction^7^ Li, ^23^ Na, and ^133^ Cs NMR spectra of in MSC membranes are shown in Figure 13, for example. These spectra are represented by narrow lines that belong to Li^+^, Na^+^, and Cs^+^ cations in appropriate ionic forms.

**Figure 14:**
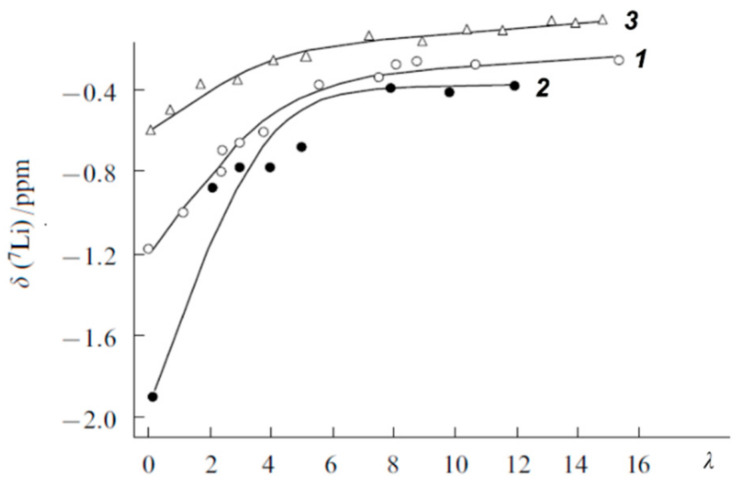
Dependences of chemical shifts in ^7^ Li NMR spectra on the moisture content of the Li^+^ forms of perfluorinated sulfonate cation-exchange membranes MF-4SC (1), Nafion 117 (2), and carboxylic membranes F-4CF (3). Reprinted with permission from [21]. Copyright 2010 Springer Nature.

Figure 14 shows the dependences of chemical shifts in ^7^ Li NMR spectra on the moisture content in the Li^+^ form of MF-4SC, Nafion 117, and carboxylic F-4CF membranes.

For water content *λ* > 4, the chemical shifts depend slightly on the membrane type and the moisture content; for *λ* < 4, the ^7^Li chemical shift for sulfonate cation exchange membranes to decrease. The half-widths of ^7^Li NMR lines at *λ* > 3–4 are almost the same for all membranes, while their line width increase is observed at *λ* < 2–3 which is due to the longer lifetimes of Li^+^ ions located on ionogenic groups.

**Figure 15:**
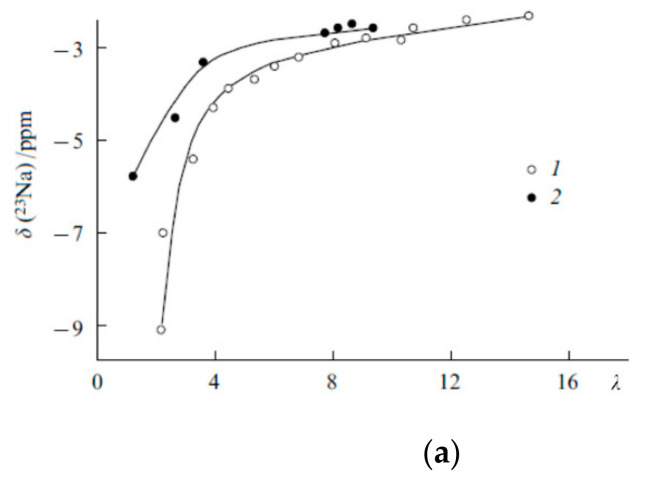

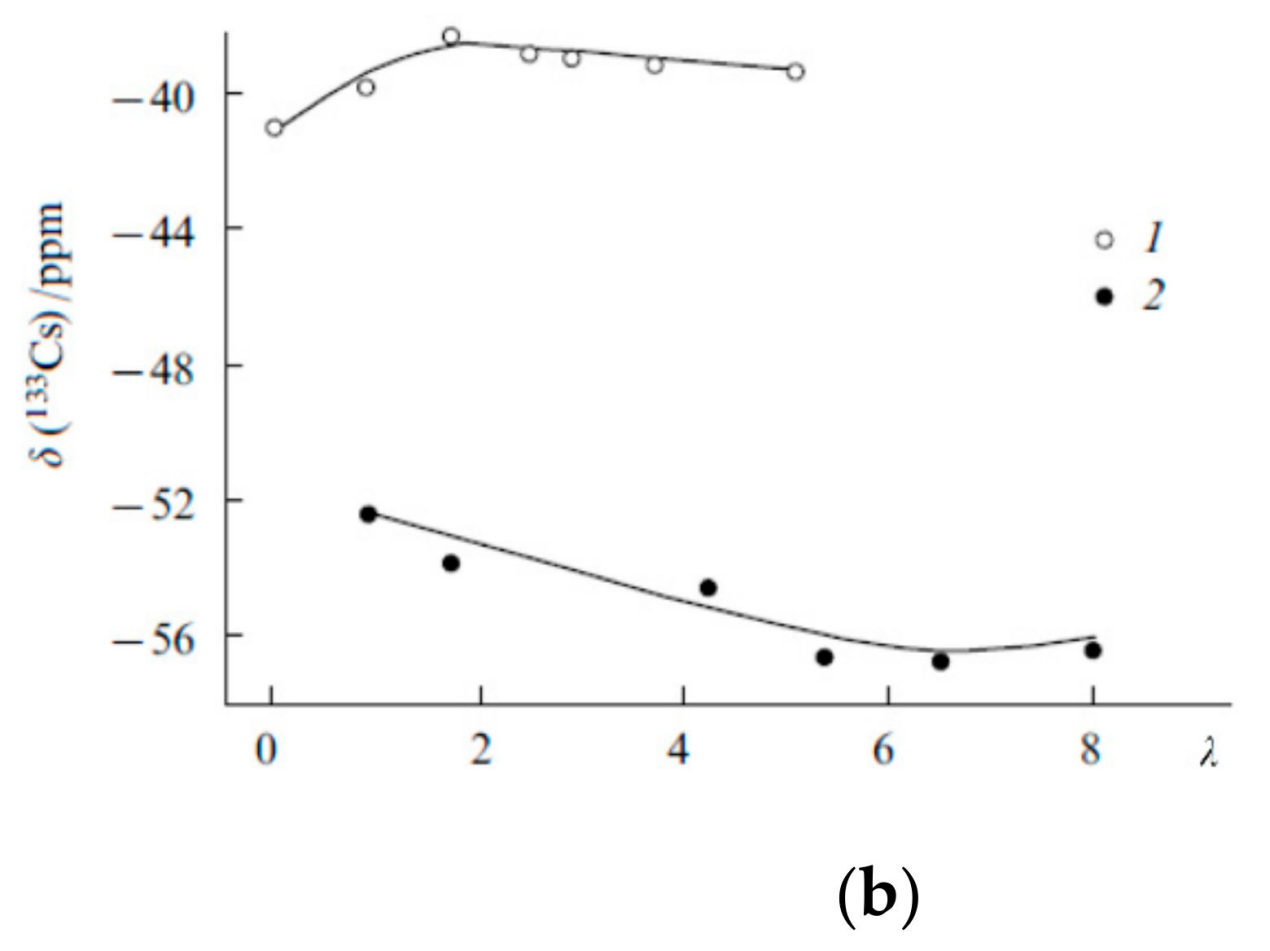
Chemical shifts in ^23^ Na NMR (**a**) and ^133^ Cs NMR (**b**) spectra vs. the moisture content of perfluorinated sulfonate cation-exchange membranes MF-4SC (1) and carboxylic membranes F-CF (2). Reprinted with permission from [21]. Copyright 2010 Springer Nature.

The dependences of chemical shifts on the moisture content in the Na^+^ form of MF-4SC and F-4CF membranes (Figure 15a) are similar to the corresponding dependences for their Li^+^ form. For *λ* > 4–5, the ^23^ Na chemical shifts are close for sulfonate cation-exchange and carboxylic membranes and start to dramatically decrease at *λ* < 4–5. As in the case of Li^+^ ions, this can be attributed to the formation of ionogenic group-cation contact pairs, which sharply reduces the sodium ion mobility.

Cs^+^ cation ^113^ Cs chemical shift humidity dependence is not so sharp in opposite to ^7^ Li and ^23^ Na ones for Li^+^ and Na^+^ cations (Figure 15b). These signals in carboxylic membranes shift in high field compare to sulfonate membranes (Figure 15).

The small influence of water content on ^133^ Cs NMR line shift and width is due to low ion hydration energy Cs^+^ ion in comparison with Li^+^ and Na^+^ hydration energies. Cesium ion hydration energy is less than water molecule hydrogen bond energy. Therefore, water molecules make own hydrogen bond network and Cs^+^ cations forming a contact ionic pair with charge groups even at high humidity. For this reason, cesium ions’ immediate surrounding does not depend on humidity which is followed by slight humidity dependence of ^133^ Cs chemical shift and line width.

Line width and chemical shift of Li^+^ and Na^+^ nuclei humidity behavior may be explained as follows. At high water content (*λ* >> *h*) a hydrate cation shell similar to a hydrate cation shell in salt aqueous solutions and cation-ionogenic group ionic pair is separated. At low water content when *λ < h* sulfonate or carboxylic group oxygen atoms may enter to cation sphere forming contact ionic pairs. In this case cation surroundings symmetry is disturbed which accompanied with the chemical shift and line width increasing owing to slowing-down cation mobility [20,21].

**Figure 16:**
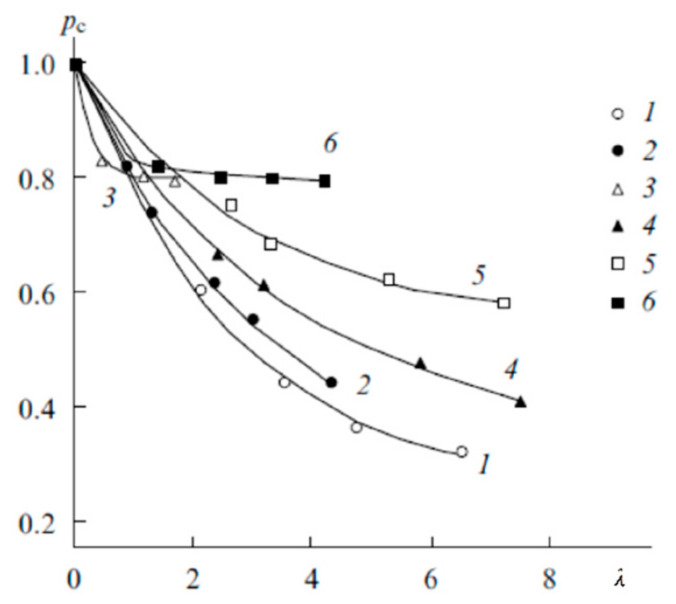
Relative parts of contact ion pairs vs. the moisture content for sulfonate cation-exchange (1–3) and carboxylic (4–6) membranes in various ionic forms. Membrane ionic form: (1, 4) Li^+^; (2, 5) Na^+^; (3, 6) Cs^+^. Reprinted with permission from [20]. Copyright 2011 Elsevier.

The chemical shift of alkali metal nuclei can be calculated by the formula:

(5) $$\delta_{i}=p_{c}\cdot \delta_{ic}+p_{b}\cdot \delta_{ib},$$

where *p_c_* and *p_b_* are the relative fractions of contact and solvent-separated ion pairs, respectively; *δ_ic_*, *δ_ib_* are the chemical shifts of ions forming either the contact or solvent-separated ion pairs [21] Using this equation, the dependences of the relative proportion of contact ion pairs on the moisture content were calculated (Figure 16).

The fraction of contact ion pairs was found to vary in the following series for both sulfonate cation-exchange and carboxylic membranes:

Cs^+^ > Na^+^ > Li^+^.

#### 1.2.2. Water Behavior at Temperature Lower 0 °C

**Figure 17:**
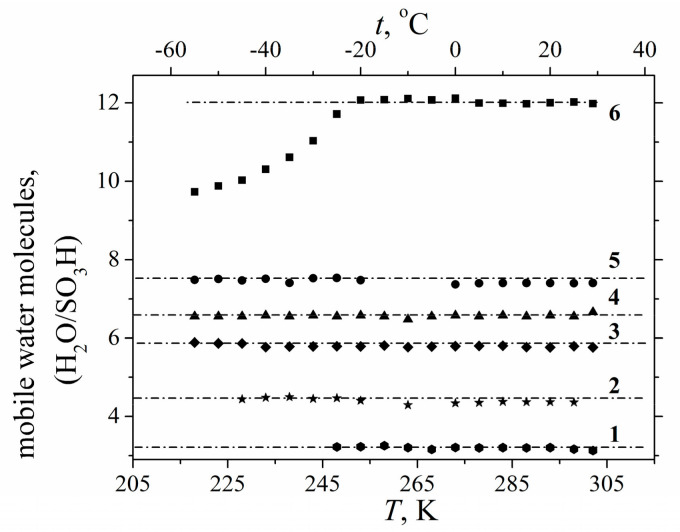
Dependences of mobile water molecule amount in acidic form of Nafion 117 membrane on temperature at different water content: (1) *λ* = 3.2 ± 0.4; (2) *λ* = 4.4 ± 0.4; (3) *λ* = 5.8 ± 0.4; (4) *λ* = 6.4 ± 0.4; (5) *λ* = 7.4 ± 0.4; (6) *λ* = 12 ± 0.4. Reprinted with permission from [25]. Copyright 2019 Springer Nature.

Hydration particularities control the structural and dynamic water behavior. On the basis of DSC measurements water in membrane nanochannels usually is divided on “unbounded” and “bounded” water. Unbounded water forms ice phase at temperature below 0 °C, while bounded water is mobile at freezing temperature [25,111,112]. On the ^1^H NMR data water hydrogen bond network in Nafion membrane is destroyed. Therefore, water molecules are not able to organize ice phase and their mobility is high at temperatures below 0 °C. This proposal explains the narrow proton NMR line width at freezing temperatures. Let us discuss this phenomenon in more details. The observed ^1^H NMR lines are caused by protons of H^+^ and mobile water molecules. The mobile water molecules amount proportional to NMR line area. The dependences of mobile water molecule amount on temperature at different water content are shown in Figure 17. The number of mobile water molecules is temperature independent until *λ* about 8 (curves 1–5 in Figure 17), the similar result was obtained by A. Guillermo and co-workers [74]. With *λ* increasing (*λ* > 9), the mobility of some water molecules at temperature below 0 °C is decreased, anyway about 8–9 water molecule per sulfonate group possess high mobility independently on temperature (curve 6 in Figure 17).

**Figure 18:**
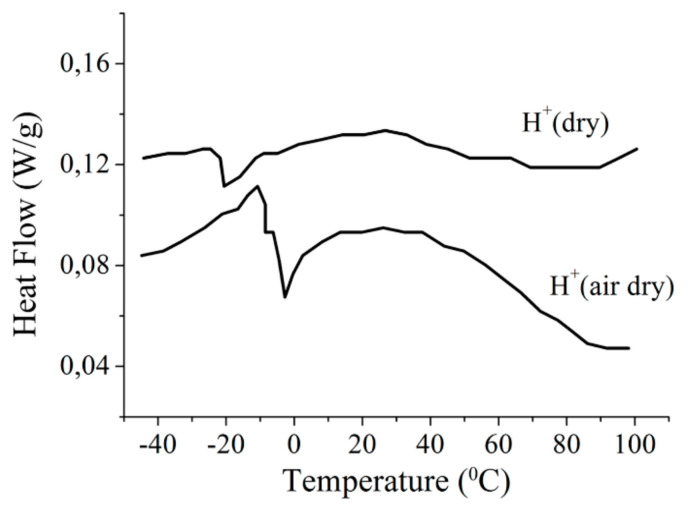
DSC thermograms in H^+^ ionic forms of perfluorinated sulfo cation exchange MF-4SC membrane, which is complete analog of Nafion. Dry–sample was dried to constant weight at 120 °C (*λ* is about 2 water molecules per sulfonate group), air dry–sample was dried to constant weight at room temperature (*λ* is about 5 water molecules per sulfonate group). Reprinted with permission from [38]. Copyright 2003 Springer Nature.

This effect may be explained, keeping in mind the existence of macroscopic dimension pores with low sulfonated group concentration in perfluorinated membranes [113]. The first water molecules are sorbed in nanochannels where ionogenic group concentration is high, after occupying these sites (about 9 molecules per sulfonated group) the other water molecules fill macropores. In macropores the water structure is similar to water structure in bulk, so this water is able to form ice phase at low temperature. Because of low ice molecule mobility their NMR line is very wide (about 22 kHz) it is impossible to observe ice molecules by high resolution NMR technique. It is also very important to mention that in spite of the fact that water does not freeze at temperatures below 0 °C the DSC thermogram peak is observed (as it is shown for example in Figure 18 [38]) which is usually interpreted as the freezing of free water [111,112,114,115].

The same water behavior is also observed for other ionic forms and type of membranes.

**Figure 19:**
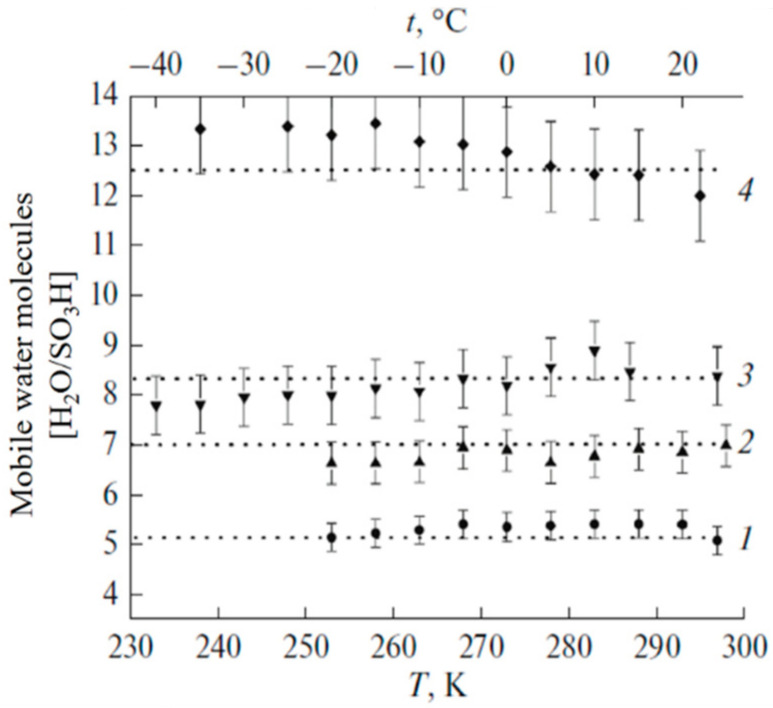
Dependence of the amount of mobile water in the H^+^ form of MSC membranes at different moisture contents in the temperature range from 25 to −40 °C. The number of water molecules per sulfo group is λ = 5.1 (1), 7 (2), 8.4 (3), 12.5 (4). Reprinted with permission from [16]. Copyright 2020 Springer Nature.

As it is shown in Figure 19, water in MSC membrane doesn’t frees until *λ* < 18 water molecules per sulfonate group [16].

The same phenomenon is observed for salt membrane forms.

**Figure 20:**
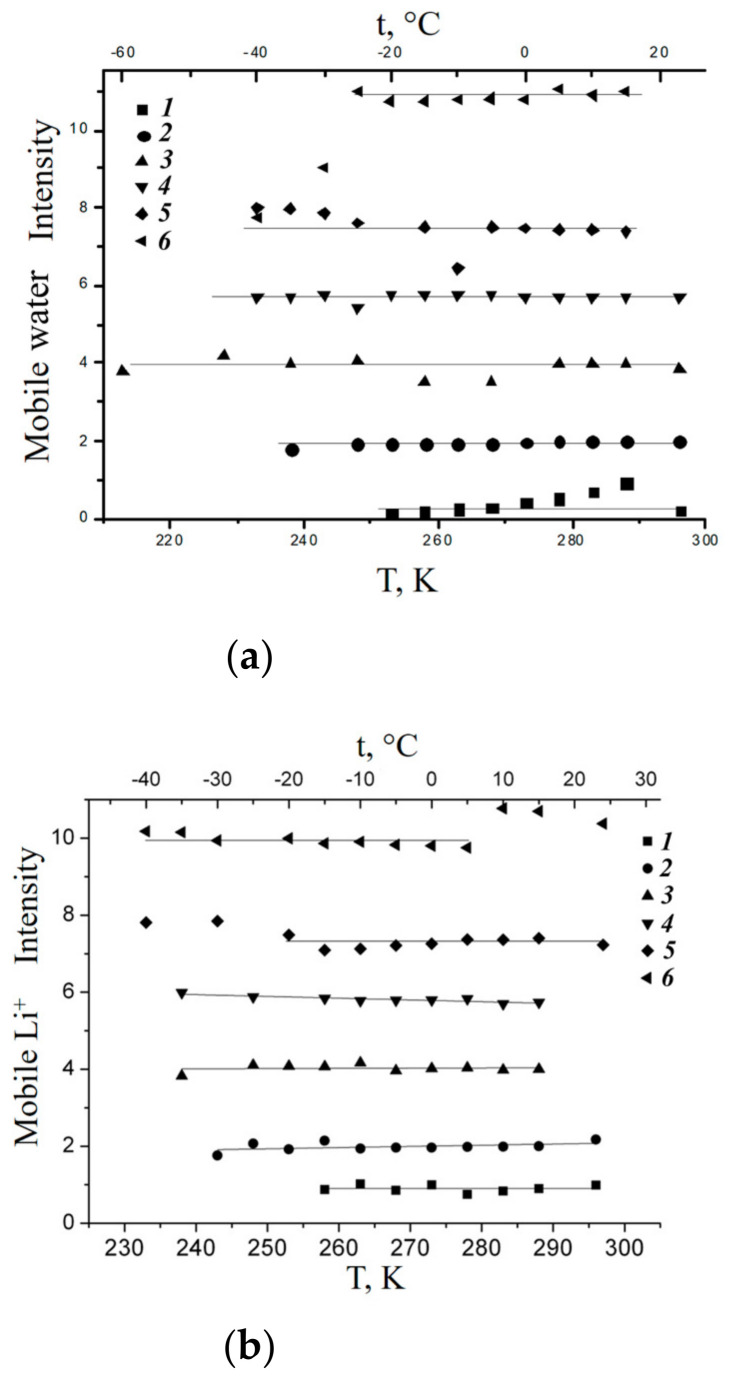
The dependences of mobile water molecules per sulfonated group *λ* (**a**) and mobile Li^+^ cations on temperature in lithium ionic form of Nafion 117 membrane (**b**); (**a**) the amount of mobile water molecules per sulfonate group: (1) *λ* = 0.9, (2) *λ* = 2.0, (3) *λ* = 4.0, (4) *λ* = 5.7, (5) *λ* = 7.4, (6) *λ* = 10.7. (**b**) a relative intensity of ^7^ Li^+^ signals: (1) *λ* = 0.9, (2) *λ* = 2.0, (3) *λ* = 4.0, (4) *λ* = 5.7, (5) *λ* = 7.4, (6) *λ* = 10.7.

In Figure 20 the independent of amount of mobile water molecules and Li^+^ cations on temperature is illustrated.

This disagreement between DSC and NMR results may be explained by the ^1^ H spin–relaxation data. Let us investigate water mobility at freezing condition in more details using NMR pulsed field gradient and NMR relaxation techniques.

### 1.3. NMR Relaxation. Local Mobility of Molecules and Ions

#### H NMR Relaxation, Local Proton Motion

Despite considerable progress in the research of ion and molecular transport in polymer electrolytes, the micro-scale transfer mechanisms are still unclear. As was stated above, the NMR methods furnish a lot of information on the local motion of ions, solvent molecules, and polymer matrices. The times of spin–lattice (*T*_1_) and spin–spin (*T*_2_) relaxation and the width of NMR line are very sensitive to local mobility. Local proton motion in Nafion membrane may be characterized by ^1^H spin relaxation data. The dependences of spin–lattice (*T*_1_) and spin–spin relaxation (*T*_2_) times on temperature and humidity are usually analyzed [35,36,37,38,41,105]. The detailed quantitative analysis based on Bloembergen, Purcell, Pound (BPP) magnetic dipole-dipole relaxation mechanism for distribution of correlation time in H^+^ and Li^+^ ionic form sulfo cationic perfluorinated membrane was carried out [31,37,38,105].

**Figure 21:**
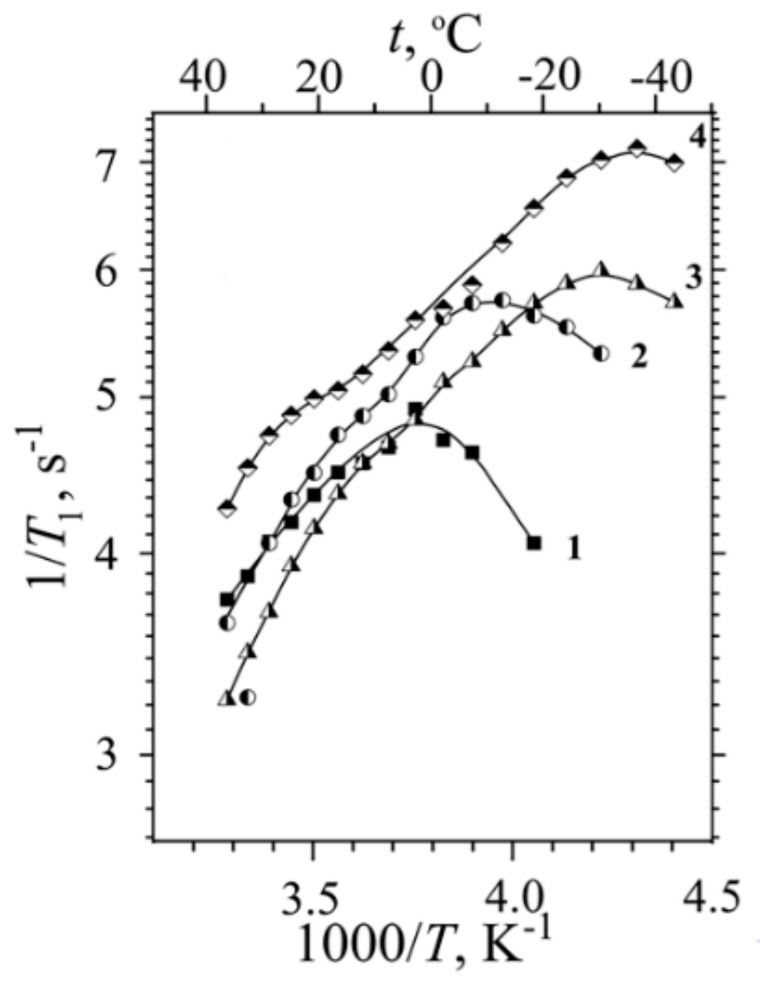
Temperature dependences of ^1^ H spin–lattice *R*_1_ (*T*_1_^−1^) relaxation rates in Nafion 117 membrane with different water content (1) *λ* = 3.2; (2) *λ* = 4.4; (3) *λ* = 5.8; (4) *λ* = 7.4. Reprinted with permission from [25]. Copyright 2019 Springer Nature.

**Figure 22:**
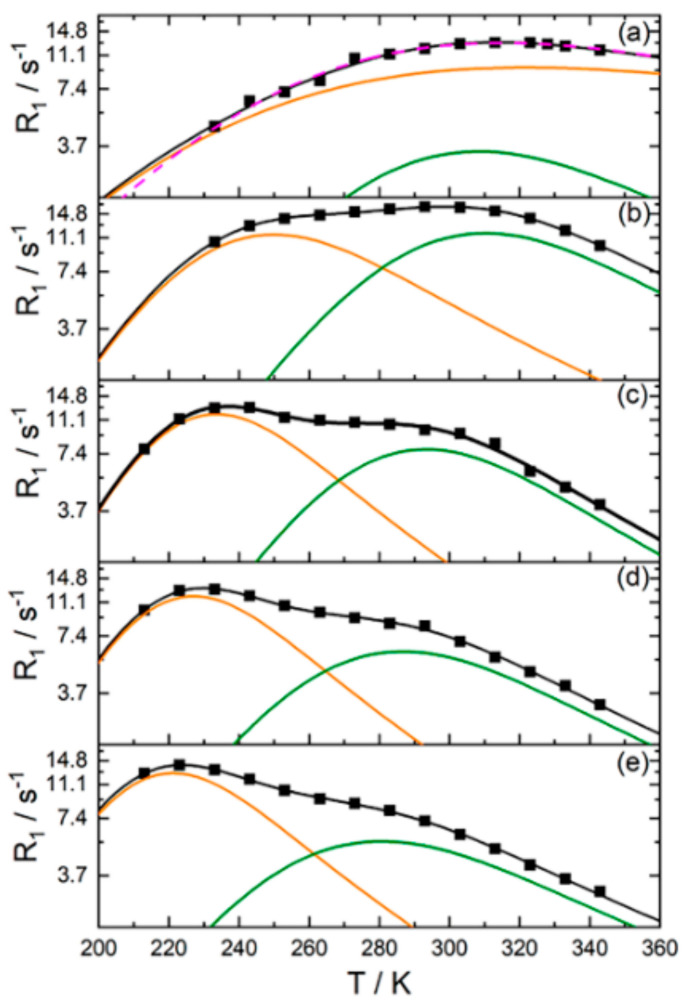
Temperature-dependent longitudinal relaxation rates (*R*_1_) at apparent hydration levels of (**a**) *λ*_water_ = 2, (**b**) *λ*_water_ = 3, (**c**), *λ*_water_ = 5, (**d**) *λ*_water_ = 7, and (**e**) *λ*_water_ = 8 for Nafion N117. Black squares represent the experimental data and the green and orange lines represent the BPP fit of the slow (mode I) and fast (mode II) motional modes, respectively; black lines show the superpositions of both contributions. The purple dashed line in (**a**) represents the best fit assuming a single BPP model Figure 4 from. Reprinted with permission from [26]. Copyright 2019 American Chemical Society.

The dependences of spin–lattice relaxation rate (*T*_1_^−1^) on temperature in H^+^ ionic form of Nafion 117 for different water content are shown in Figure 21 and Figure 22. These dependences show wide maximum in low temperature region and maximum or shoulder in high temperature region. Spin–lattice relaxation rate low temperature maximum is shifted to high temperature region with decrease of membrane humidity.

The authors of [26] interpreted the *T*_1_^−1^(*T*) shape by two phases proton relaxation as a result of two different types of water motion in Nafion membrane. This interpretation is not unequivocal. For more defined interpretation the mutual analysis of proton spin–lattice (*T*_1_) and spin–spin (*T*_2_) relaxation processes is necessary. This investigation has been carried out in Li^+^ ionic form of perfluorinated sulfo cation exchange membrane MF-4SC [31,37,38,105]. The temperature dependences of *T*_1_^−1^(*T*) and *T*_2_^−1^(*T*) in samples with different water content were measured.

To interpret the measured relaxation times, it is necessary to determine the types of interactions and molecular motions.

For nuclei with spin 1/2, the principal relaxation interactions are the magnetic homo- and heteronuclear dipole–dipole couplings. The NMR relaxation for nuclei with spin > 1/2, e.g., ^7^ Li, is mainly determined by two mechanisms:

- quadrupole relaxation due to interactions between the quadrupole moment of a nucleus with the fluctuating electric field gradient induced by the charge distribution around this nucleus;
- dipole–dipole relaxation caused by random fluctuations of magnetic moments of nuclei.

It was shown that the magnetic dipole–dipole coupling is the main interaction inducing relaxation of water protons. The magnetic relaxation of ^7^ Li nuclei is modulated by the ^7^ Li−^1^ H magnetic dipole–dipole coupling and the quadrupole coupling between lithium nuclei and the electric field gradient. The ^1^H relaxation was described in terms of the Bloembergen–Purcell–Pound model, which takes into account the distribution of correlation times in the low-temperature region. The distribution of correlation times are either Gaussian or rectangular [31,37,38]. The processes of spin–lattice relaxation of ^7^ Li nuclei were determined by rapid librations of water molecules within the cation hydration shell, which allowed their lifetime to be calculated [38]. In contrast to the kinetics of longitudinal magnetization decay, the kinetics of transverse magnetization decay was biexponential.

Detailed analysis of spin–spin relaxation processes was carried out. The spin–spin relaxation was assumed to be modulated by slower motions as compared with librations of water molecules within the hydration shell of a lithium cation. These motions are associated with variations of the electric field gradient on a ^7^ Li nucleus as the latter approaches (or moves away from) a sulfo group. Based on these data, the lifetime of lithium cations on sulfo groups was calculated. The algorithms of calculation of correlation times of water molecules, the lifetimes of lithium hydration shells, and lithium cations located on sulfonic groups for MF-4SC membranes can be found in several publications [31,37,38]. Below, we present the main conclusions made in these publications.

**Figure 23:**
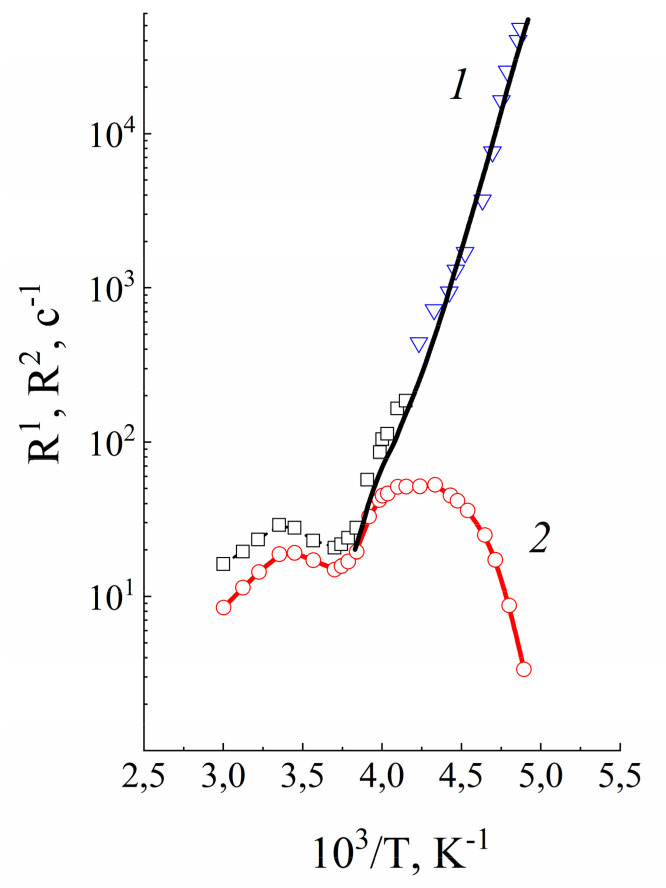
Temperature dependences of spin–lattice (*R*_1—_curve 2) and spin–spin (*R*_2—_curve 1) relaxation rates of ^1^ H water molecule nuclei in Li^+^ ionic form of MF-4SC membrane at water content *λ* = 20.5 water molecules per sulfonate group (adapted from [37,38]).

**Figure 24:**
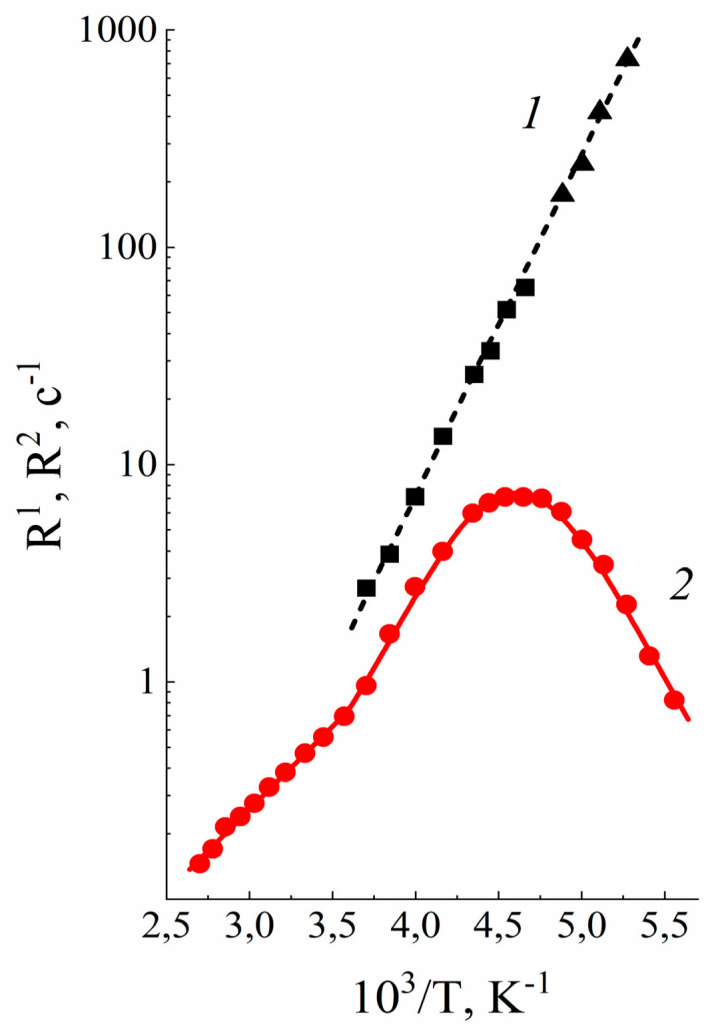
Temperature dependences of spin–lattice (R_1_—curve 2) and spin–spin (R_2_—curve 1) relaxation rates of ^7^ Li lithium cation nuclei in Li^+^ ionic form of MF-4SC membrane at water content *λ* = 20.5 water molecules per sulfonate group (adapted from [31,38]).

Figure 23 and Figure 24 show temperature dependences of the longitudinal and transverse relaxation rates of ^1^ H and ^7^ Li nuclei.

Water molecule ^1^ H relaxation rates show two maximums of *R*_1_ and minimum of *R*_2_ (Figure 23). The minimum *R*_2_ is an unusual shape because for two phase relaxation process (the phase populations are temperature independent) *R*_2_(*T*) increasing with decreasing temperature smoothly without extremum [105]. The only way to explain a spin–lattice and spin–spin relaxation together, it was proposed, that the patterns of motion of water molecules are different in the low- (*T* < 280 K) and high-temperature (*T* > 320 K) ranges. In this case the two phases relaxation is also taken place, but the phase populations *p_l_* and *p_h_* are temperature dependent.

(6) $$\frac{1}{T_{1}\left(T\right)}=\frac{p_{l}\left(T\right)}{T_{1l}\left(T\right)}+\frac{p_{h}\left(T\right)}{T_{1h}\left(T\right)}$$

**Figure 25:**
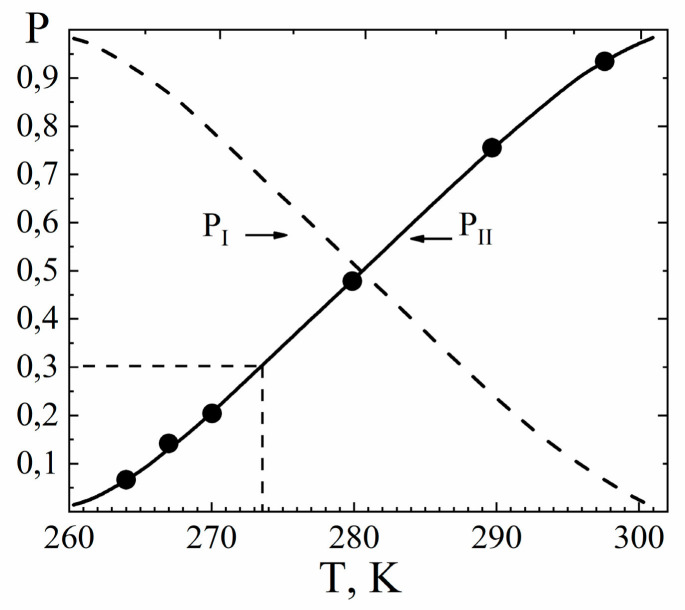
Temperature dependences of population *p_l_* (dashed line) and *p_h_* (solid line) for Li^+^ ionic form of MF-4SC membrane at *λ =* 20.5 (adapted from [37,38]).

This was connected with the fact that at low temperatures, water molecules form associates that are highly mobile at *T* < 0 °C because in membranes, water molecules interact with charged ionic groups and cannot form the solid phase (ice). In the high-temperature region, water molecules can move as in electrolyte solutions. In the intermediate temperature range, both phases coexist. Figure 25 shows the temperature dependence of the high-temperature *p_h_* (solid line) and low temperature *p_l_* (dashed line) phase populations. The transition from one phase to another is accompanied by absorption or released energy due to the formation (rupture) of hydrogen bonds. Note that the observed thermal DSC effect was not connected with a freezing (melting) of free water, because according to NMR data, the number of mobile water molecules did not change upon the phase transition [25,38]. Associate formation, but not water crystallization shows DSC peak.

As it will be shown, the assumption of water molecule association below 0 °C, have explained the shape of self-diffusion coefficients temperature dependences.

### 1.4. Pulsed Field Gradient NMR. Diffusion and Ionic Conductivity

Pulsed field gradient NMR is very attractive for ion and molecular diffusion investigation in ion-exchangers because it is a direct technique of partial self-diffusion coefficient and diffusant relative content characterization [14,15,16,17,20,21,24,25,44,45,46,47,48,49,50,51,52,53,54,55,56,57,58,59,60,61,62,63,64,65,66,67,68,69,70,71,72,73,74,75,76,77,78,79,80,81,105,109,115]. Stimulated echo sequences are generally used for measurements [42,43]. We have reviewed the water molecule and alkali metal ion diffusion data, which are compared with ionic conductivity results [16,17,21,25,55,56,57,58,59,60,61,105]. Some details of pulsed field gradient NMR (PFG NMR) technique and self-diffusion coefficients calculations are given in Appendix A.

#### 1.4.1. Pulsed Field Gradient ^1^ H NMR, Water Molecule Self-Diffusion and Ionic Conductivity

**Figure 26:**
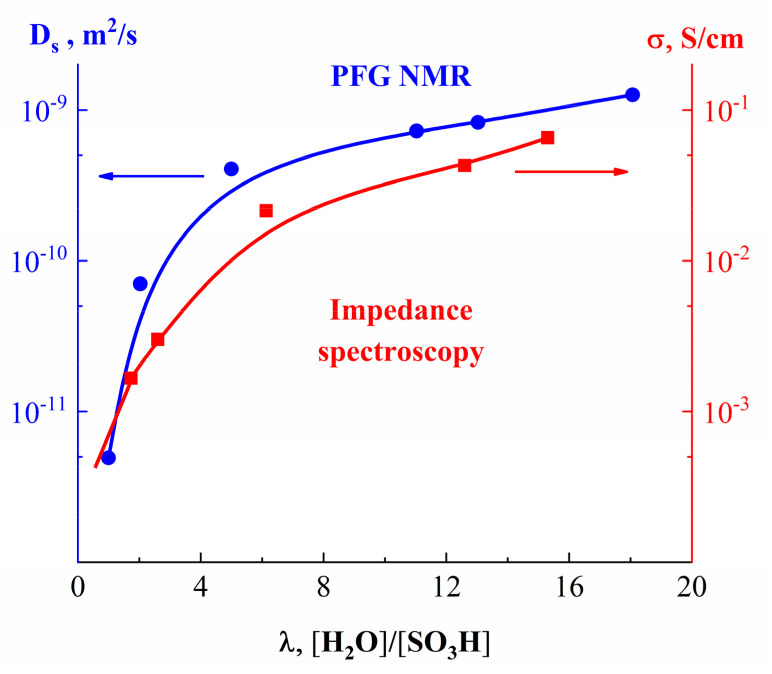
Dependences of water self-diffusion coefficient and proton conductivity on humidity (*λ*), where *λ* is water molecule amount per sulfonate group in acid form of MF-4SC membrane [57].

As it is shown in Figure 26, water molecule translational mobility controls an ionic conductivity.

**Figure 27:**
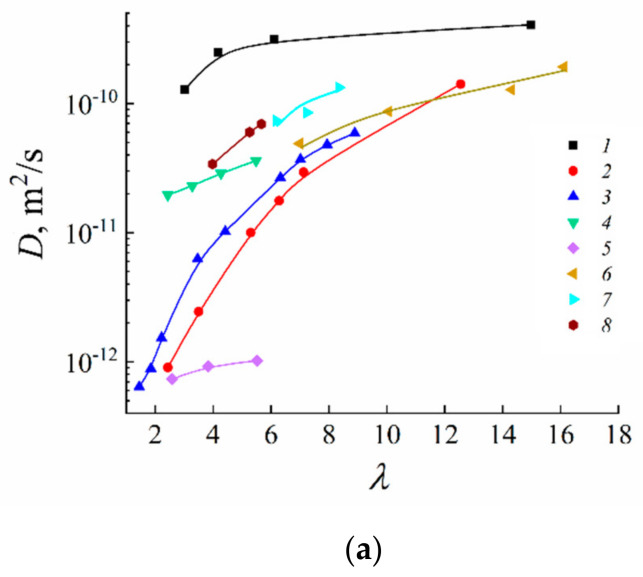

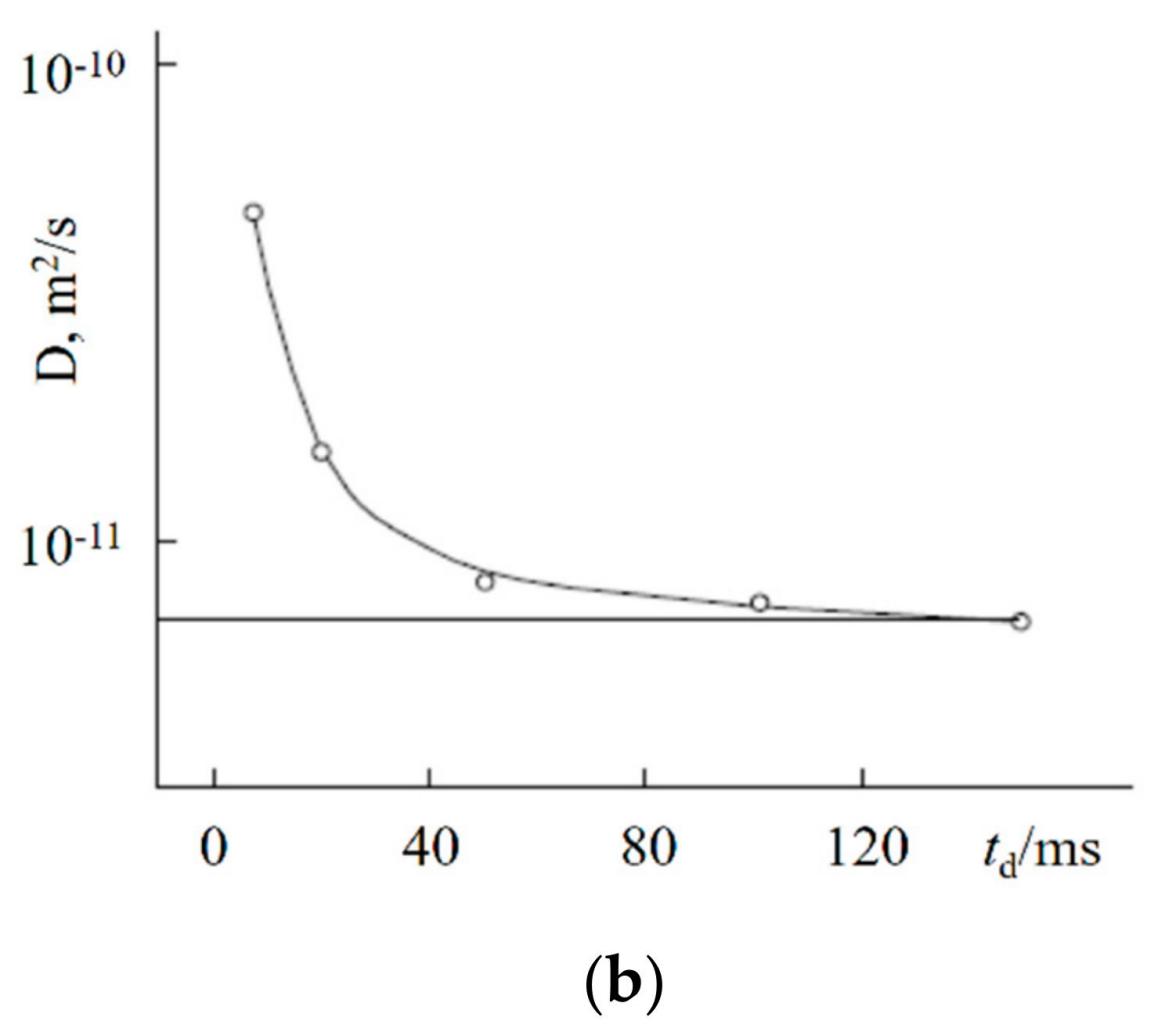
(**a**) Diffusion coefficients of water molecules dependence on the moisture content for sulfonate cation-exchange MF-4SC (1–4) and carboxylic F-4CF (5–8) perfluorinated membranes in various ionic forms. (**b**) Diffusion coefficients dependence of water molecules and hydrated H^+^ counterions in the acidic form of F-4CF membrane on the diffusion time, relative humidity is 95% (**b**). Membrane ionic forms: (1, 5) H^+^, (2, 6) Li^+^, (3, 7) Na^+^, (4, 8) Cs^+^. Reprinted with permission from [20]. Copyright 2011 Elsevier.

Figure 27 shows the dependence of diffusion coefficients of water molecules on the moisture content in perfluorinated MF-4SC and F-4CF membranes of various ionic forms. The diffusion coefficients of water molecules (and hydrated H^+^ counter-ions for a membrane in its acidic form) vary in the following sequence of ionic forms for sulfonate cation exchange membranes at the same water content:

H+ > Cs+ > Na+ > Li+

The identical series of water diffusion coefficients are typical for carboxylic membranes in the salt form. However, in contrast to MF-4SC membranes, in the acidic form of carboxylic membranes, water and hydrated H^+^ cations behave much differently. In carboxylic membranes, the diffusion coefficients are two orders of magnitude smaller than in sulfonate cation-exchange membranes (see curves 5 and 1 in Figure 27a). In carboxylic membranes, restricted diffusion occurs, which is manifested as a decrease in the diffusion coefficients of water and hydrated H^+^ cations with increase in the diffusion time (Figure 27b). The size of restriction regions is about one µm and decreases with a decrease in the moisture content. Presumably, these restrictions are induced by the formation of internal hydrogen bonds between carboxylic groups of neighboring macromolecules.

**Figure 28:**
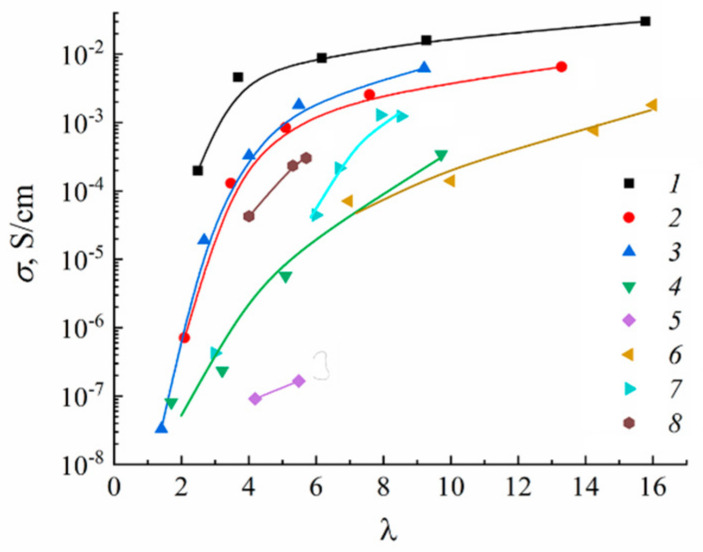
Conductivity dependence on the moisture content for sulfonate cation-exchange MF-4SC (1–4) and carboxylic F-4CF (5–8) perfluorinated membranes in various ionic forms. Membrane ionic forms: (1, 5) H^+^, (2, 6) Li+, (3, 7) Na+, (4, 8) Cs+. Reprinted with permission from [20]. Copyright 2011 Elsevier.

The dependences of ionic conductivity of membranes on the moisture content have a similar form as the analogous dependences for diffusion coefficients (Figure 28) [20,21]. In the region *λ* < *h*, the conductivity of samples sharply drops down as the moisture content decreases. In sulfonate cation-exchange membranes, the conductivity varies in the following series at maximum water content H^+^ >> Na^+^ ≈ Li^+^ > Cs^+^, whereas in carboxylic membranes, the inverse dependence is observed. Sulfonate cation-exchange membranes in their Li^+^ and Na^+^ forms exhibit higher conductivity as compared with carboxylic membranes. For their Cs^+^ form, the inverse dependence was observed. This probably can be explained by the larger radius of cesium ions. The interaction of Cs^+^ with the membrane matrix and water molecules is fairly weak, even for low degrees of hydration. Moreover, for low degrees of hydration, hopping of cesium cations between the oxygen atoms of ionogenic groups is rather limited by the distance between the oxygen atoms, which is similar for membranes of both types, and not by the negative charge on oxygen ions. For carboxyl membranes, this distance may be smaller, due to a larger negative charge on oxygen atoms.

The conductivity of carboxylic membranes in their acidic form is four orders of magnitude lower than the conductivity of sulfonate membranes in the same form.

The ionic conductivity was calculated based on the Nernst–Einstein equation:

(7) $$\sigma ={\mathrm{ne}}^{2}\frac{D}{\mathrm{kT}}$$

where *n* is the number of charge carriers, cm^3^; *D* is the self-diffusion coefficient, m^2^/s; *e* is the electron charge,1.9·10^–19^ C; *k* is the Boltzmann constant, 1.38·10^–23^ J/K; and *T* is the temperature.

The obtained results were compared with the results of direct measurements (Table 6).

For MF-4SC membranes in the Li^+^ form, the experimental value of conductivity and the value calculated from diffusion coefficients of lithium cations coincide to within the experimental error (6.2·10^−3^ and 6.5·10^−3^ S·cm^−1^, respectively). The ionic conductivity found from water diffusion coefficients is only several times higher than the conductivity measured experimentally. The similarity of these values suggests that the translational displacements of the cations and water molecules are correlated.

**Figure 29:**
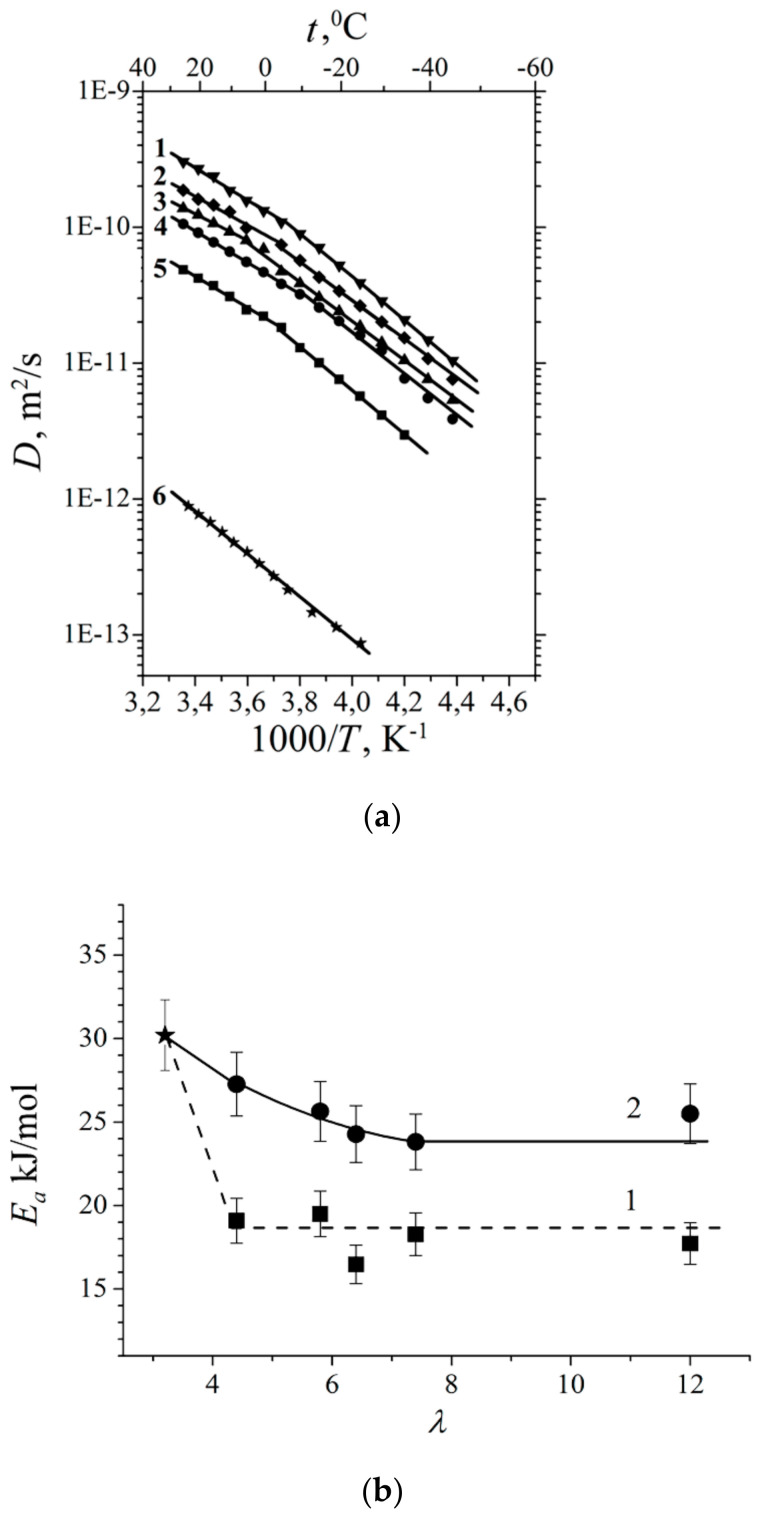
(**a**) The temperature dependences of average H+ and water-self diffusion coefficients for Nafion 117 membrane with different water content: curve (1) *λ* is 12; curve (2) *λ* is 7.4; curve (3) *λ* is 6.4; curve (4) *λ* is 5.8; curve (5) *λ* is 4.4; and curve (6) *λ* is 3.2; (**b**)water self-diffusion activation energies in high temperature region (squares—curve 1) and low temperature region (circles—curve 2). Reprinted with permission from [25]. Copyright 2019 Springer Nature.

Temperature dependences of self-diffusion coefficients at different water content are shown in Figure 29a. The main features of the curves are different slopes at high and at low temperature regions [16,19,25,38,74,105,109,115]. High and low temperature parts were approximated by the Arrhenius equitation:

(8) $$D=D_{0}\cdot e^{-\frac{E_{a}}{R\cdot T}}$$

where *D_o_* is temperature independent, *R* is gas constant, *T* is absolute temperature, *E_a_* is self-diffusion activation energy.

The corresponding activation energies for different humidities are shown in Figure 29b. At high humidity the activation energies do not depend on water content and are close to bulk water activation energy. At low water content, high and low temperature activation energies increase and become closer to each other. The changes of the slope in temperature dependences is not observed for *λ* less than four water molecules per sulfonate group (curve 6, Figure 29a, star in Figure 29b).

**Figure 30:**
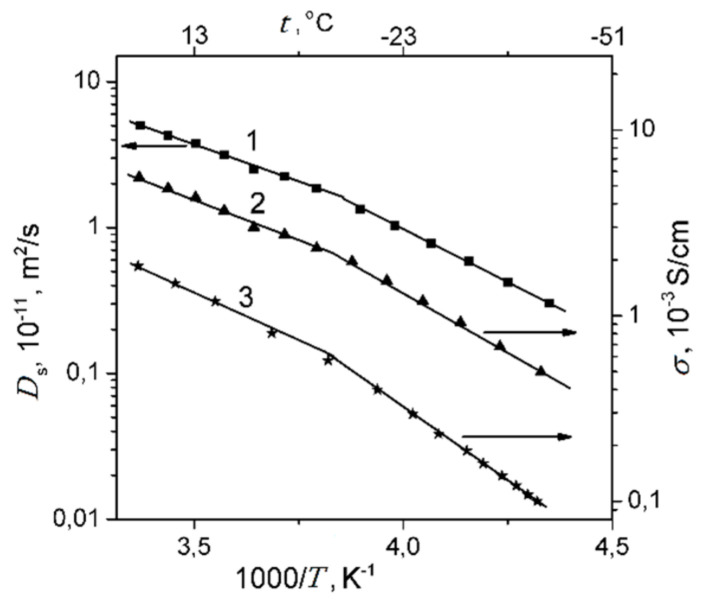
Temperature dependences of self-diffusion coefficients—curve 1 and calculated from Equation (7) protonic conductivity—curve 2, *λ* = 4.4 [25]. Curve 3—experimental temperature dependence of conductivity for *λ_g_* = 3.7 [112].

Proton conductivities temperature dependences [25,78,79,80,81,111,112] are very similar to the temperature dependences of self-diffusion coefficients. Temperature dependences of self-diffusion coefficients, protonic conductivities calculated from Nernst–Einstein Equation (7), and experimentally measured conductivities are shown in Figure 30 for *λ* ≈ 4–5 as an example.

The calculated conductivities are 3–4 times bigger compared to the experimental values. There are some reasons for this difference. On the one hand, the measured self-diffusion coefficient is characterized as average translation mobility of water molecules and hydrated H^+^ and it is impossible to distinguish H^+^ translational mobility, which controls a charge transfer. On the other hand, not every cation jump is accompanied by charge transfer. The changes in the conductivity and self-diffusion curve slopes are observed at the same temperatures. The increase in the slopes of the self-diffusion and proton conductivity curves at low temperatures is usually explained by the freezing of free (unbound) water at temperatures below 0 °C [81,111,112].

In our opinion this explanation contradicts to the temperature dependence of the mobile water amount. As it was found above water does not freeze until *λ* = 9–10 (Figure 17), nevertheless the change in the slope on temperature dependences is observed at *λ* less than this water content (Figure 30a). It is also very important to mention that in spite of the fact that water doesn’t freeze at temperatures below 0 °C the DSC thermogram peak is observed (as it is shown for example in Figure 18 [38]), which is usually interpreted as the freezing of free water [81,111,112,114].

**Figure 31:**
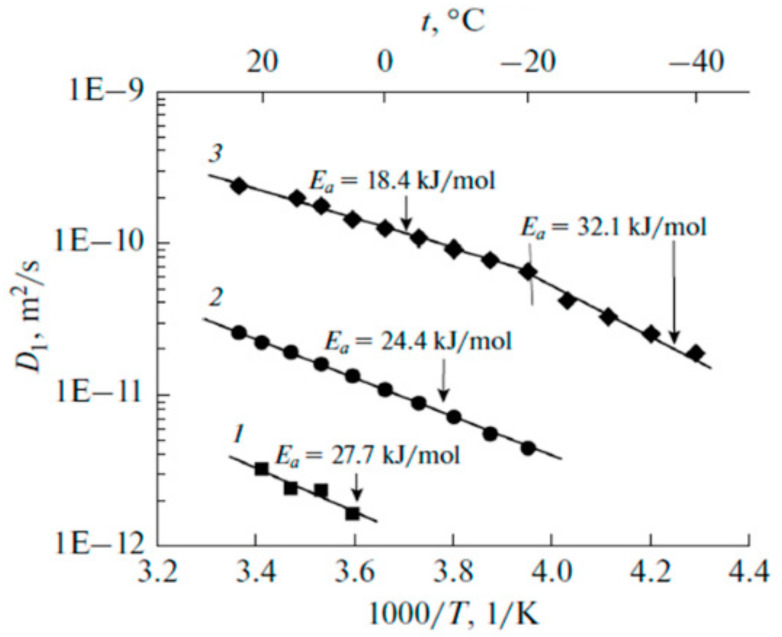
Temperature dependence of the diffusion coefficients *D* measured by pulsed field gradient NMR at different water contents in acid form of MSC membrane: *λ* = 5.1 (*1*), 7 (*2*), 8.4 (*3*). Reprinted with permission from [16]. Copyright 2020 Springer Nature.

This disagreement may be explained by the ^1^ H spin–relaxation data. As it was shown above at low temperature water molecules form associates, therefore the self-diffusion activation energy is higher compare to high temperature region, where the water structure is similar to bulk water one. The temperature dependence of the diffusion coefficients *D* in the MSC membrane held at different relative humidity is also linearized in the coordinates of the Arrhenius Equation (7) (Figure 31).

The main feature of the temperature dependences of the self-diffusion coefficients is an increase in the self-diffusion activation energy with a decrease in moisture content in the entire temperature range. This phenomenon is similar to Nafion self-diffusion coefficient temperature dependences. It should be also mentioned, that water in MSC membrane is mobile at freezing temperatures until *λ* = 18 [16].

**Figure 32:**
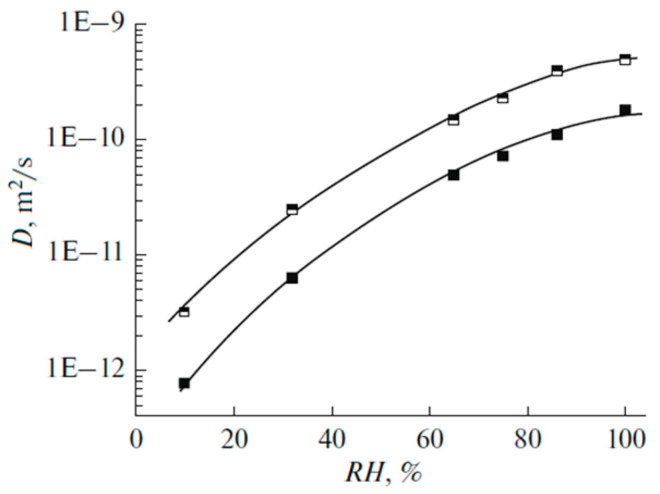
Diffusion coefficients of H^+^ cations calculated from the proton conductivity data according to Equation 1.7 (lower curve) and the average diffusion coefficients of water molecules and H^+^ cations measured by pulsed field gradient NMR (upper curve) in the H^+^ form of the MSC membrane at different moisture contents. *RH* = 100%, the membrane is in contact with water. Reprinted with permission from [16]. Copyright 2020 Springer Nature.

Experimental diffusion coefficients and those calculated from Nernst–Eistein Equation (7) at different moisture contents are shown in Figure 32.

It is noteworthy that the dependences of the diffusion coefficients of H^+^ cations calculated from the conductivity and measured by NMR on the moisture content are similar. The values of diffusion coefficients decrease with decreasing relative humidity. The values of the diffusion coefficients determined by NMR are higher than those calculated based on the data on conductivity. This is quite typical for ion-exchange membranes and arises because the conductivity is limited by proton transfer in narrow channels (Figure 1) of the membranes, while NMR characterizes the averaged mobility of protons and water in larger pores and channels [16,17].

#### 1.4.2. Pulsed Field Gradient ^7^ Li, ^23^ Na, ^133^ Cs NMR, Alkaline Metal Cation Self-Diffusion

Self-diffusion of water molecules and Li^+^, Na^+^, Cs^+^ cations were investigated in appropriate salt ionic form of Nafion 117 membranes.

Lithium cations and water molecules

**Figure 33:**
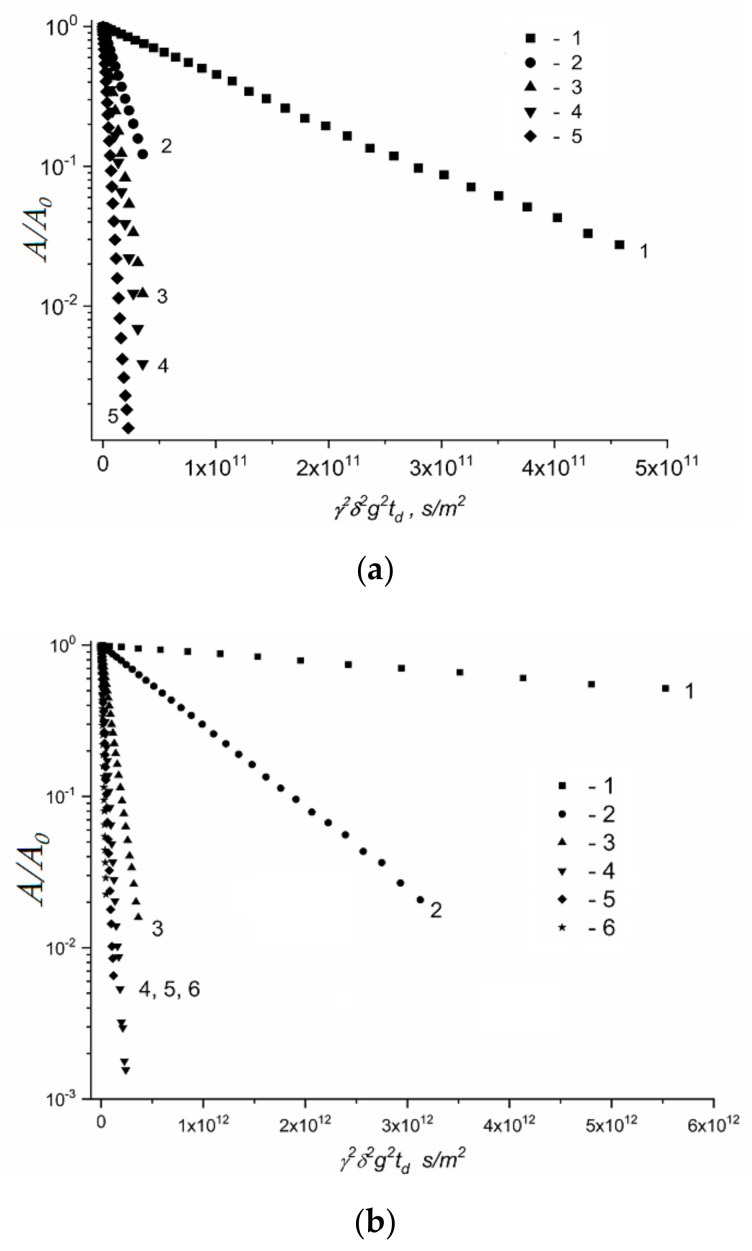
(**a**) Spin–echo signal attenuation of water molecule ^1^H nuclei dependences on gradient pulsed amplitude *A*(*g*) (diffusion decay) in Li^+^ ionic form of Nafion membrane at different water content: (1) *λ* = 2.0, (2) *λ* = 4.0, (3) *λ* = 5.7, (4) *λ* = 7.4, (5) *λ* = 10.7. (**b**) Spin-echo signal attenuation of ^7^ Li nuclei dependences on gradient pulsed amplitude *A*(*g*) (diffusion decay) in Li^+^ ionic form of Nafion membrane at different water content: (1) *λ* = 0.9, (2) *λ* = 2.0, (3) *λ* = 4.0, (4) *λ* = 5.7, (5) *λ* = 7.4, (6) *λ* = 10.7. Reprinted with permission from [109]. Copyright 2021 Elsevier.

Lithium cations and water molecules self-diffusion in Li^+^ Nafion ionic form were measured at different water contents. Spin–echo signal attenuations (diffusion decays) of ^7^ Li nuclei of Li^+^ cation and water molecule ^1^ H nuclei are shown in Figure 33.

**Figure 34:**
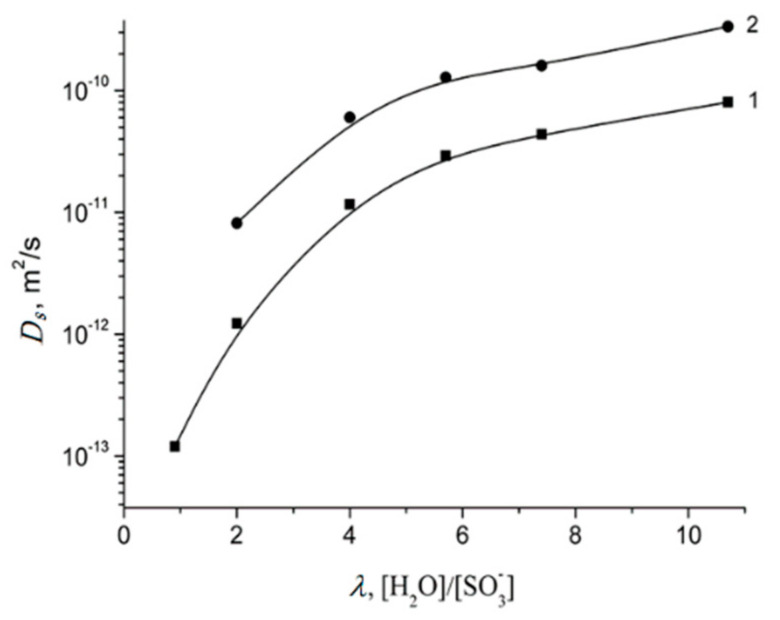
Self-diffusion coefficients of Li^+^ ions (curve 1) and water molecules (curve 2) dependences on water content *λ* in Nafion 117 membrane Li^+^ ionic form, where *λ* is water amount per sulfonate group. Reprinted with permission from [109]. Copyright 2021 Elsevier.

Spin–echo attenuations were exponential approximated by Equation (A2) in three orders of magnitudes in the whole range of water contents. It indicates that translation mobility of water molecules and lithium cations was characterized as the only self-diffusion coefficient. Self-diffusion coefficients of lithium cation and water molecule dependences on water content *λ* are given in Figure 34.

These dependences shapes are similar. It may be concluded that translational motions of Li^+^ cation and hydrated water molecules are correlated as it was indicated in [105].

**Figure 35:**
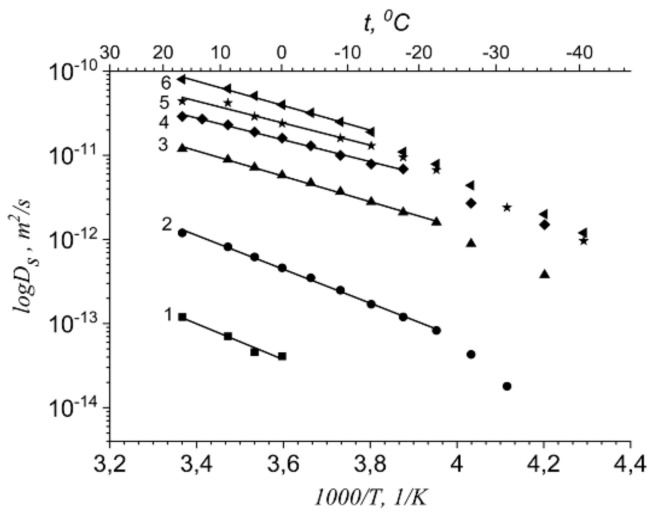
Li^+^ cation self-diffusion coefficient temperature dependences for Nafion membrane Li^+^ ionic form at different water content *λ*, where *λ* is water amount per sulfonate group. (1) *λ* = 0.9, (2) *λ* = 2.0, (3) *λ* = 4.0, (4) *λ* = 5.7, (5) *λ* = 7.4, (6) *λ* = 10.7. Reprinted with permission from [109]. Copyright 2021 Elsevier.

**Figure 36:**
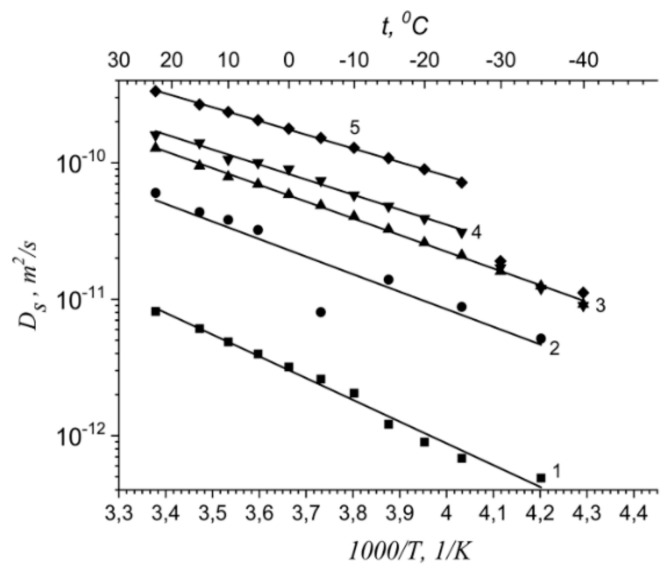
Water molecule self-diffusion coefficient temperature dependences for Nafion membrane Li^+^ ionic form at different water content *λ*, where *λ* is water amount per sulfonate group. (1) *λ* = 2.0, (2) *λ* = 4.0, (3) *λ* = 5.7, (4) *λ* = 7.4, (5) *λ* = 10.7. Reprinted with permission from [109]. Copyright 2021 Elsevier.

Temperature dependences of Li^+^ and water molecules self-diffusion coefficients at different moisture content are shown in Figure 35 and Figure 36.

The dependences *D_s_*(*T*) were approximated by Arrhenius Equation (8).

Equation (8) describes well Li^+^ self-diffusion in temperature region from −10 °C to +30 °C (Figure 35) and water molecule self-diffusion from −40 °C to +30 °C (Figure 36). Activation energies at different water content *λ* summarize in Table 7.

Activation energies *E_a_* increase with hydration degree decreasing. Lithium cation self-diffusion activation energies are essentially more compare to water molecule self-diffusion activation energies.

Sodium and cesium cation self-diffusion

**Figure 37:**
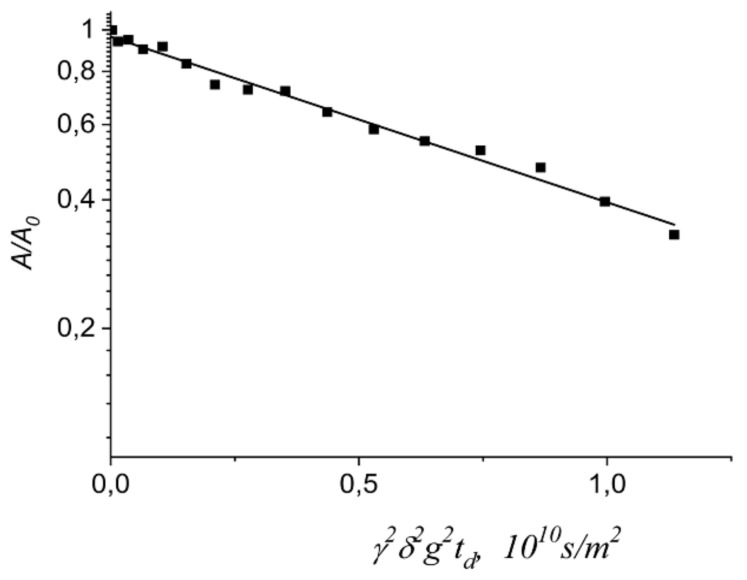
Diffusion decay of ^23^ Na nuclei in Nafion membrane Na^+^ ionic form, *RH* = 95%, *t* = 20 °C. Reprinted with permission from [109]. Copyright 2021 Elsevier.

**Figure 38:**
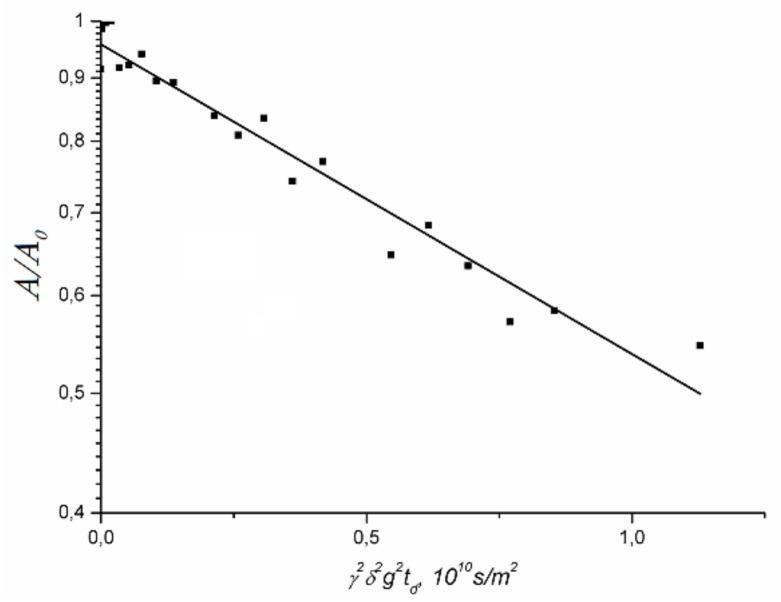
Diffusion decay of ^133^ Cs nuclei in Nafion membrane Cs^+^ ionic form, *RH* = 95%, *t* = 20 °C. Reprinted with permission from [109]. Copyright 2021 Elsevier.

The measurement of Na^+^ and Cs^+^ self-diffusion coefficients is more effortful compare to Li^+^ self-diffusion coefficient. Until now the sodium and cesium self-diffusion data in Nafion membrane are unknown. There are two reasons of these difficulties. First is low magnetic moment of ^23^ Na and ^133^ Cs nuclei. The second is the large quadrupole moment of these nuclei. The main spin relaxation mechanism of ^23^Na and ^133^Cs nuclei is quadrupole moment–gradient ligand electric field interaction. We have observed these nuclei diffusion decays in aqueous solution and in sulfocation-exchange membranes (MSC) based on polyethylene sulfonated grafted polystyrene [16,17], which maximum water content *λ* is more compared to Nafion membrane. At this condition cation water molecule shell is high-symmetric. Therefore, electric field gradient is low and nuclear spin–spin relaxation time is long enough to observe spin–echo signal. We also succeeded to get spin–echo attenuation in Nafion membrane at *RH* = 98%. The examples of ^23^ Na and ^133^ Cs decays of Na^+^ and Cs^+^ cations are given in Figure 37 and Figure 38.

**Figure 39:**
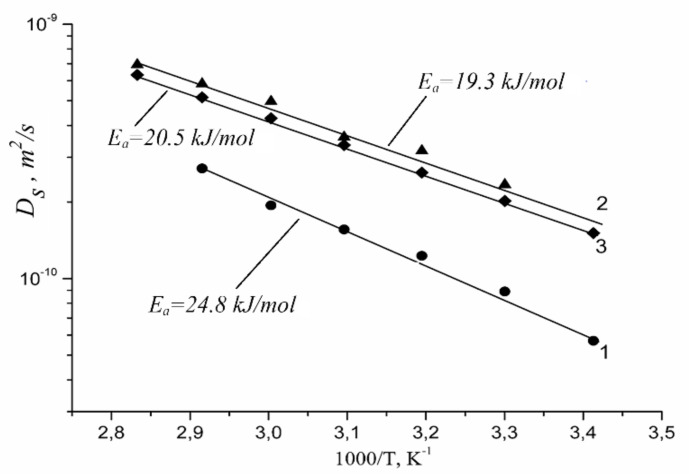
Temperature dependences of Li^+^ (curve 3), Na^+^ (curve 2), Cs^+^ (curve 1) self-diffusion coefficients in Li^+^, Na^+^, Cs^+^ Nafion 117 membrane ionic forms. *RH* = 98%. Reprinted with permission from [109]. Copyright 2021 Elsevier.

Lithium, sodium, and cesium cation self-diffusion coefficient temperature dependences in Nafion membrane at maximum humidity (*RH* = 95%) in the temperature range from 20 °C to 80 °C are shown in Figure 39.

**Figure 40:**
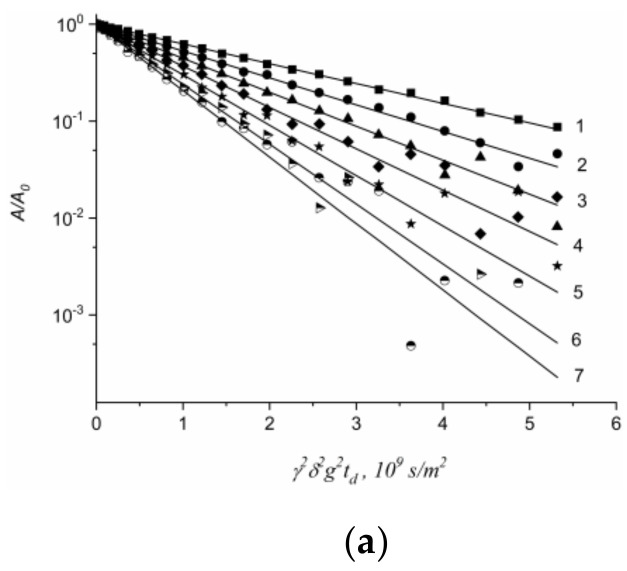

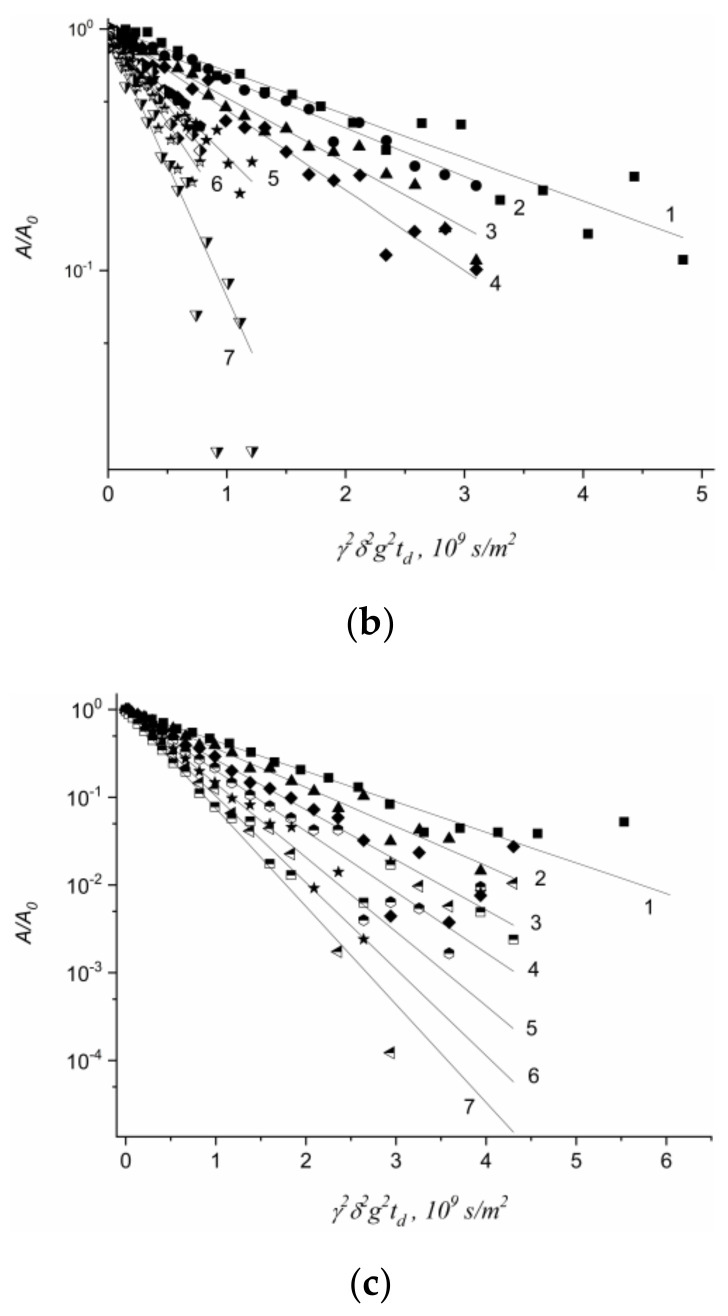
Diffusion decays of ^7^ Li (**a**), ^23^ Na (**b**), ^133^ Cs (**c**) nuclei NMR signals in appropriate ionic form of MSC membrane at *RH* = 95% and different temperatures (1) 20 °C, (2) 30 °C, (3) 40 °C, (4) 50 °C, (5) 60 °C, (6) 70 °C, (7) 80 °C [17].

The dependences *D_s_*(*T*) were approximated by Arrhenius Equation (8). Self-diffusion coefficients increase in the next sequence Li^+^ ≈ Na^+^ > Cs^+^. Let us consider cation self-diffusion behaviors in MSC membrane. Spin–echo attenuation (diffusion decay) of ^7^ Li^+^, ^23^ Na^+^, and ^133^ Cs^+^ is exponential in salt ionic form of MSC membrane. Diffusion decay is well approximated by Equation (A2)–Figure 40.

**Figure 41:**
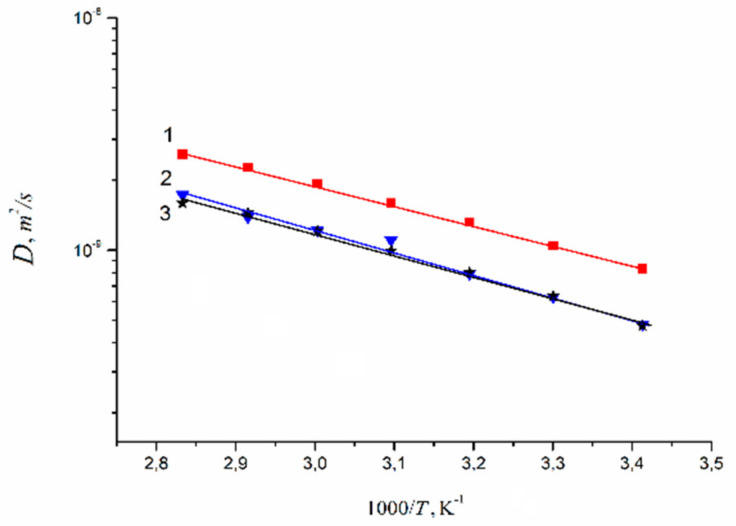
Temperature dependences of Cs^+^, Na^+^, Li^+^ diffusion coefficients in appropriate ionic form of MSC membrane at *RH* = 95%: (1) Cs^+^ ionic form, *E_a_* = 18.1 kJ/mol; (2) Na^+^ ionic form, *E_a_* = 16.5 kJ/mol; (3) Li^+^ ionic form, *E_a_* = 17.6 kJ/mol [17].

Diffusion coefficient temperature dependences are also linearized in the coordinates of the Arrhenius equation (Figure 41). Cation diffusion coefficients increase in a sequence Li^+^ ≈ Na^+^ < Cs^+^. This row is the same for cation diffusion coefficients of chloride aqueous solutions [17]. Cation diffusion activation energies are about 16–18 kJ/mol.

Self-diffusion coefficients at 20 °C, self-diffusion activation energies of Li^+^, Na^+^, and Cs^+^ cations in Nafion membrane are indicated in Table 8. The values of Li^+^, Na^+^, Cs^+^ self-diffusion coefficients and activation energies in MSC sulfonic cation-exchange membrane and dilute chloride aqueous solution are also given for comparison [17].

Self-diffusion activation energies of Li^+^ and Na^+^ cations in Nafion 117 membrane are about 20 kJ/mol which is close to water self-diffusion activation energy in these membranes [16,37,105], but for Cs^+^ activation energy is distinctly more compared with Li^+^ and Na^+^ cations. It should be mentioned that in MSC membrane cation self-diffusion activation energies are little less and Cs^+^ activation energy are even smaller than Li^+^ and Na^+^ compare with Nafion membrane. Cation self-diffusion coefficients are changed in the next rows Li^+^ ≤ Na^+^ > Cs^+^ in Nafion; Li^+^ < Na^+^ < Cs^+^ in MSC [17] and chloride aqueous solutions. Higher Cs^+^ activation energy and lower self-diffusion coefficient in Nafion compared to aqueous solution and MSC membrane may be explained in the following way. As it was mentioned above as opposed to Li^+^ and Na^+^, Cs^+^ cation interacts with Nafion SO_3_^−^ group directly and a contact ionic pair is forming. This is the reason of low mobility and high self-diffusion activation energy of cesium cation in Nafion.

#### 1.4.3. Lithium, Sodium, Cesium Cation Self-Diffusion and Ionic Conductivity

Ionic conductivities *σ_c_* of Li^+^, Na^+^, and Cs^+^ were calculated in Nafion membranes on the basis of Nernst–Einstein Equation (7). Calculated conductivities *σ_c_* was compared with experimental values *σ_e_* measured by impedance spectroscopy. The data are listed in Table 9.

Ionic conductivities calculated from cation self-diffusion coefficients are appreciably more compared with experimental meanings. The following discrepancy reason may be the next: ionic conductivity is controlled only by cation transfer along applied electric field, but PFG NMR fixes all translational jumping of cation, for instance, cation coming and leaving of sulfonate group.

**Figure 42:**
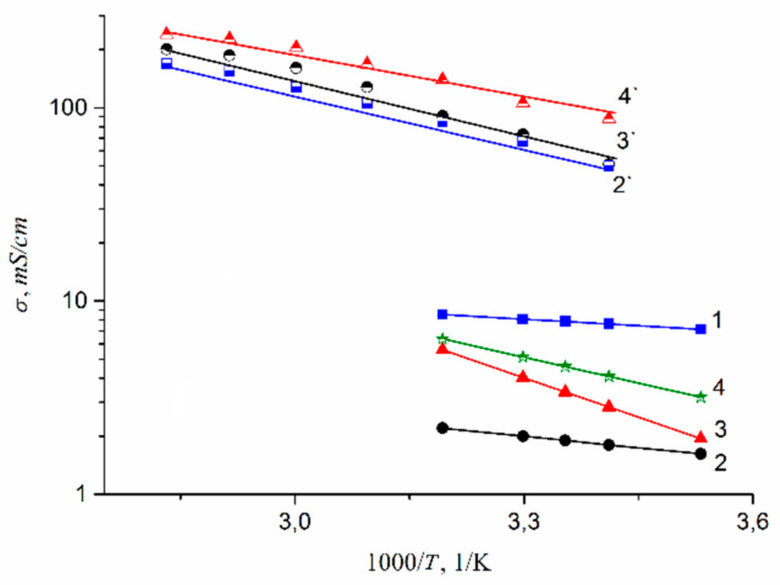
Temperature dependences of experimental *σ_exp_* (1–4) and calculated *σ_calc_* (2′–4′) ionic conductivities in H^+^ (1), Li^+^ (2) and (2′), Na^+^ (3) and (3′), Cs^+^ (4) and (4′) ionic forms of MSC membrane at *RH* 95%.

Temperature dependences of experimental and calculated from Nernst–Einstein equation ionic conductivities of different ionic forms of MSC membranes are shown in Figure 42.

The conductivity values at different humidity and activation energies calculated from Arrhenius equation are listed in Table 10.

Ionic conductivity of investigated membranes increases in the sequence Li^+^ < Na^+^ < Cs^+^ << H^+^. It should be noted that the diffusion coefficients of lithium, sodium, and cesium cations in MSC and in aqueous solutions change in the same sequence.

As it is shown in Figure 42, the calculated and experimental conductivity curve are similar. Conductivity activation energies of alkaline metal cations are close to each other. However, the calculated conductivity values are in one or two orders of magnitude more in comparison with experimental ones. This difference seems natural. Ionic transport in these membranes is realized through the system of channels and pores, which size is depended on polymeric matrix nature and hydration degree (Figure 1). Ionic conductivity is limited by the transport of ions in the narrow channels. They are usually called the “bottle neck” [116,117]. The diffusion coefficient measured by NMR in the first turn may be due to high mobility ions localized in wide pores [16].

#### 1.4.4. Li^+^, Na^+^, and Cs^+^ Hydration and Diffusion in Chloride Aqueous Solutions

**Figure 43:**
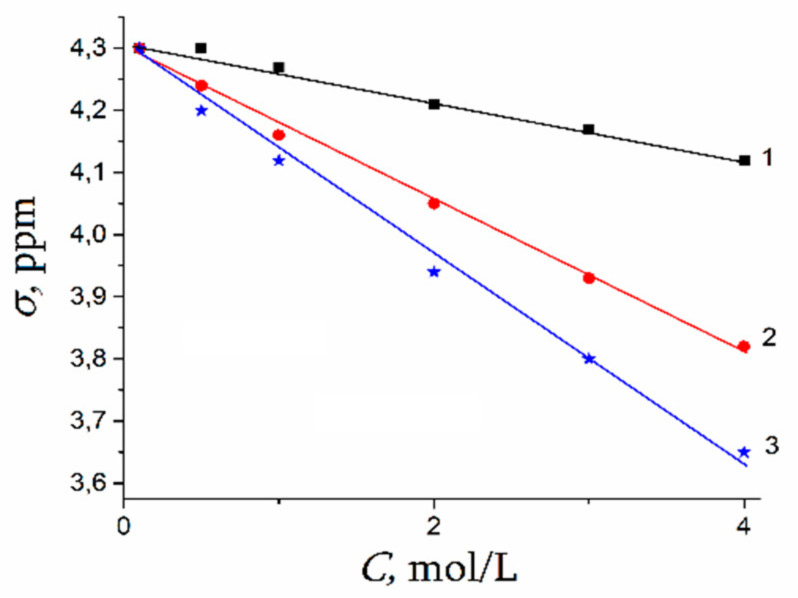
^1^ H chemical shift dependences on concentration of LiCl (1), NaCl (2), CsCl (3) aqueous solutions [17].

A dependence of ^1^H water molecule chemical shift on solution concentration is shown in Figure 43. Proton signal shifts to the high field with chloride concentration increasing. This fact is explained by destroying of hydrogen bonds between water molecules [58,103,104,105]. This phenomenon is stronger for CsCl solution since Cs^+^ possesses low polarizing properties (due to the large ion size), which causes a hydrogen bond system destroying [17].

**Figure 44:**
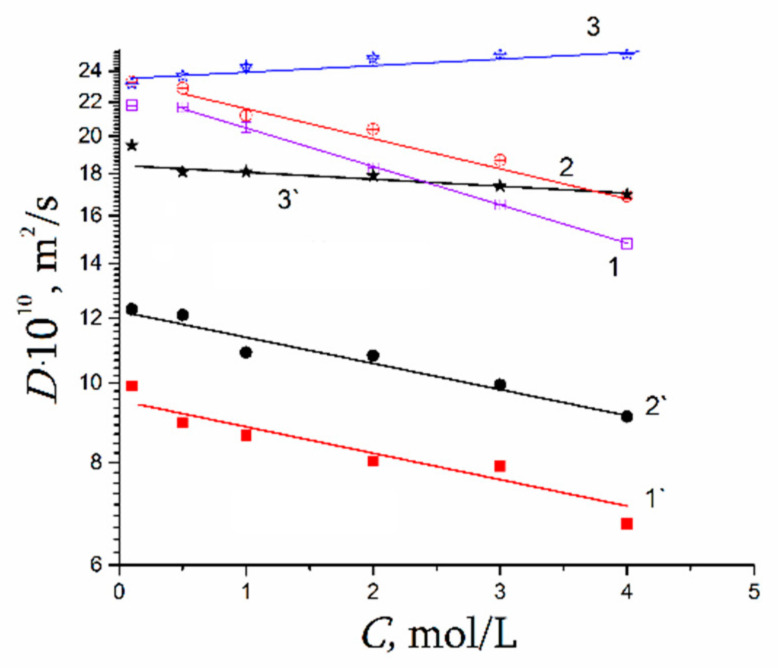
Water molecule and cation diffusion coefficient concentration dependences in lithium, sodium, cesium chloride aqueous solutions. (1) H_2_O in LiCl, (2) H_2_O in NaCl, (3) H_2_O in CsCl. (1′) Li^+^ in LiCl, (2′) Na^+^ in NaCl, (3′) Cs^+^ in CsCl [17].

As a result, water molecule translational mobility in CsCl aqueous solution should increase with increasing concentration, which is observed experimentally (curve 3 in Figure 44).

Water and alkaline metal cation self-diffusion coefficients increase in the row of cation atomic mass growing: Li^+^ < Na^+^ < Cs^+^. This increasing sequence credits with cation hydrated radius decreasing due to ion hydration energy reduction from Li^+^ to Cs^+^. In opposition to membrane, measured ionic self-diffusion coefficients in dilute salt aqueous solution are equal to diffusion coefficients calculated from ion conductivity data [118]. This indicates that conductivity and PFG NMR are measuring different kinds of ionic mobility in membranes. Self-diffusion coefficient obtained by PFG NMR is an average self-diffusion coefficient while ionic conductivity is restricted by ion motion in narrow channels.

It should be mentioned that Li^+^, Na^+^, and water diffusion coefficients are reduced greater compared to Cs^+^ ion for which water diffusion coefficient even increases with an increase in electrolyte concentration (curves 1′, 2′;1, 2 Figure 44 and curves 3′, 3 Figure 44). The water hydrogen bond network is destroying to a greater extent with increasing in Cs^+^ concentration. As contrasted to Cs^+^, hydration energy of Li^+^ and Na^+^ ions is more. Therefore, mobility of water molecules connected with these cations drops and water diffusion coefficient is reduced with salt concentration rising.

Now the question should be considered about the reason of the self-diffusion coefficient decreasing with solution concentration growing. Water molecules hydrate both cations and anions. Cation hydrate shell structure is rebuilt during ion motion. The neighboring ions obstruct to hydrated shell forming and consequently ionic mobility decreases. This obstruction factor rises with concentration growth or ion activity coefficient decreasing [17]. Therefore, the water and ion self-diffusion coefficients are reduced with solution concentration increasing.

**Figure 45:**
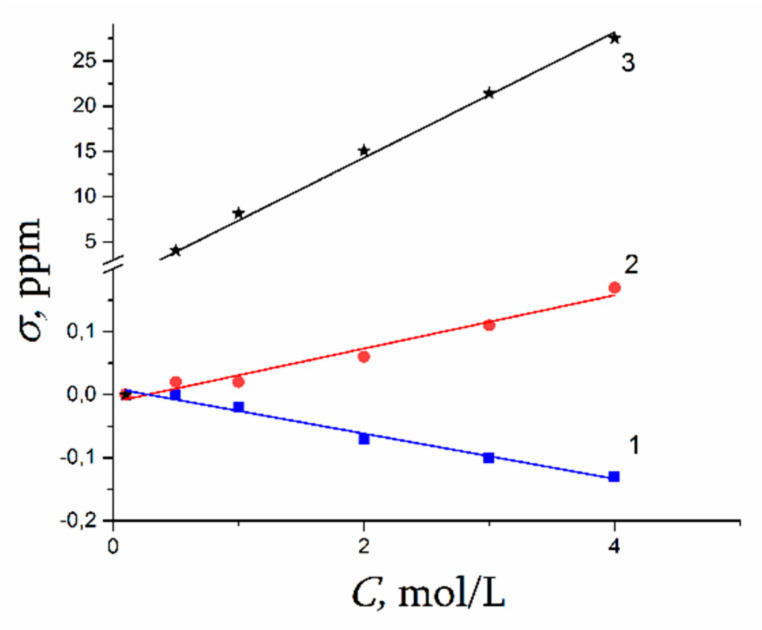
^7^ Li (1), ^23^ Na (2), ^133^ Cs (3) nuclear NMR chemical shift concentration dependences in lithium, sodium, cesium chloride aqueous solutions [17].

In Figure 45 ^7^ Li, ^23^ Na, ^133^ Cs nuclei chemical shift concentration dependences are shown. This dependence is stronger for ^133^Cs. Chemical shift increases sometimes (compared to ^7^ Li, ^23^ Na), while CsCl concentration is varied from 1 mol/L to 4 mol/L (curve 3, Figure 45).

The chemical shift of these nuclei is determined by nuclear quadrupole moment interaction with gradient of electric field induced by hydration molecules of water. Because high hydration energy lithium and sodium cation hydrated shells are steady and symmetric. The electric field symmetry and consequently a cation nuclear chemical shift only insignificantly varies with concentration changes (curves 1, 2 Figure 45). In contrast to lithium and sodium, cesium cation hydration shell is not steady therefore a symmetry of hydrated electric field on Cs^+^ decreases with cesium chloride solution concentration rising which accompanied by appreciable increasing of ^133^Cs chemical shift (curve 3, Figure 45).

#### 1.4.5. Specific Features of Translational Mobility of Water Molecules and Ions at a Low Moisture Content

For water molecule, cation self-diffusion, and conductivity dependence on humidity at low water content in MF-4SC membranes explanation, the percolation theory was applied. The equation for diffusion (conductivity) coefficient dependence on the amount of water molecule per sulfonated group (*λ*) derived by professor S.F. Timashev [5]:

(9) D=D0exppa−λ13μ

**Figure 46:**
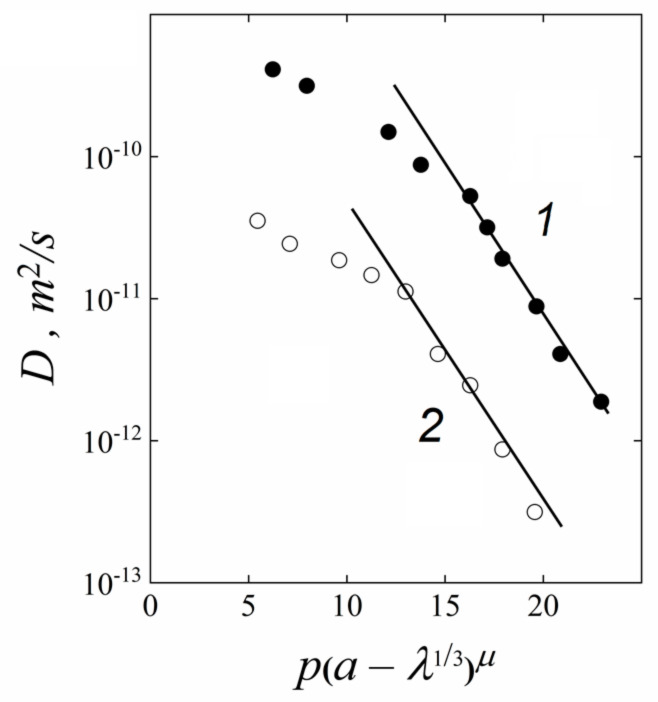
Self-diffusion coefficient dependences on humidity of water molecules (1) and lithium cations (2). Reprinted with permission from [39]. Copyright 2002 Springer Nature.

The physical sense of parameter *a, p,* and *µ* explained in [5]. This equation describes well self-diffusion coefficients of water molecule and lithium cation humidity dependences at low water content (Figure 46).

**Figure 47:**
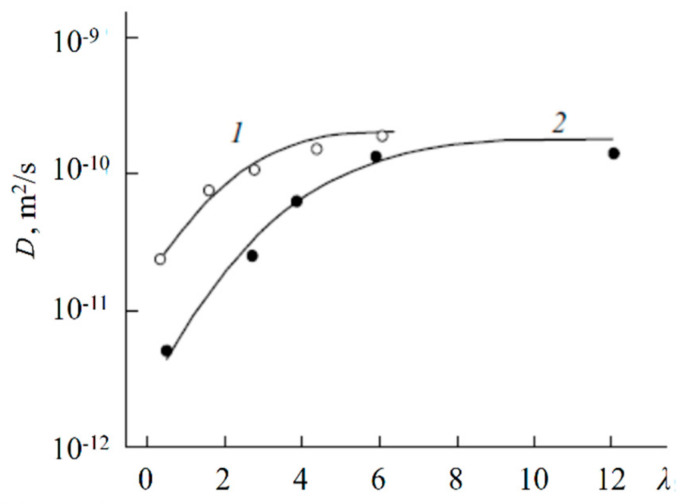
Diffusion coefficients of water on the moisture content in the acidic form of membranes MF-4SC with the exchange capacity of 0.86 (1) and 0.34 mg-equiv.∙g^−1^ (2) [39,105]. Reprinted with permission from [105]. Copyright 2013 Turpion Limited.

For effective membrane application, high water and appropriately cation high translation mobility is very important. As mentioned above, the high proton (ionic) conductivity is explained by a continuous network of hydrogen bonds. Hydrogen bond destruction is attended by threshold water and cation mobility reduction. The high ion mobility at low humidity may be achieved by reduction of charge group spacing because in this case for hydrogen bond network forming the smaller value of water molecules per sulfonate group *λ* is necessary. As it is shown in Figure 47, water self-diffusion coefficients at low *λ* rise in an order of magnitude, while SO_3_^−^ group concentration (ion-exchange capacity) increases in two times.

The next way to increase ionic mobility at low water content is to insert oxygen atoms in polymer matrix. This oxygen “bridge” forms additional hydrogen bonds for water molecule motions. For this goal achievement, Nafion membranes were modified by inorganic dopants. The membrane modification and membrane conductivity properties specify in [119].

**Figure 48:**
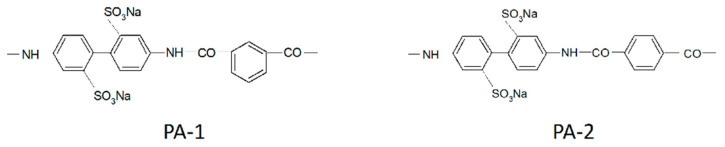
Chemical structure of sulfocontaining aromatic polyamides PA-1 and PA-2.

The next way is to synthesize ion exchange polymers containing oxygen atoms in its structure. This idea was realized in aromatic sulfo-containing polyamides, which fragments are shown in Figure 48.

**Figure 49:**
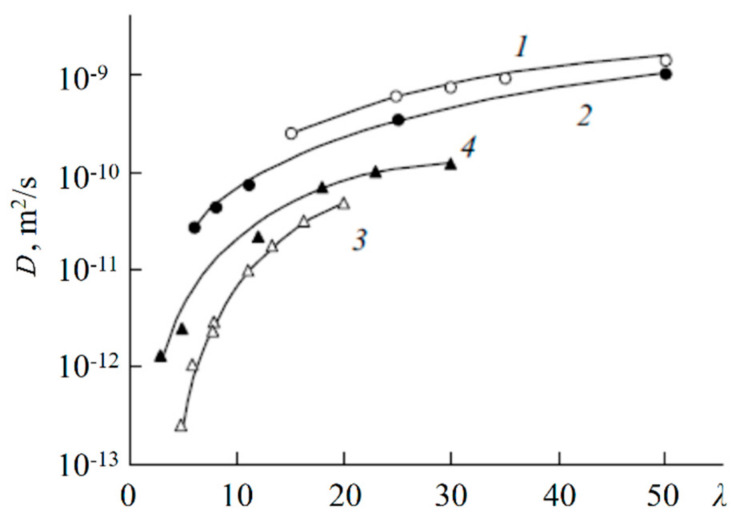
Diffusion coefficients of lithium cations in the system lithium salt-disulfophthalic acid-water ((1) is terephthalic, (2) is isophthalic) as a function of the moisture content in macroporous sulfonate cation-exchange CU-23 (4) and perfluorinated sulfonate cation-exchange membrane MF-4SC (3) [59]. Exchange capacity/mg-equiv∙g^−1^: 0.86 for MF-4SC, 5 for macroporous cation exchanger CU-23, 2 for aromatic disulfo-containing polyamides. Reprinted with permission from [59]. Copyright 2010 Springer Nature.

Figure 48 shows the diffusion coefficients of lithium cations in sulfonate membranes with different structures of transport channels and in isomeric disulfo-containing aromatic polyamides as a function of the moisture content [20,39,62]. In systems with amide groups, which serve as bridges, reinforcing the network of hydrogen bonds between the sulfo groups, the diffusion coefficients of Li^+^ ions are two orders of magnitude higher than in systems containing only sulfo groups: MF-4SC and macro pore sulfocation exchange resin on the basis of sulfonated polystyrene CU-23. The structure of ionogenic transport channels substantially affects the translational mobility of ions and molecules. Thus, although the exchange capacity of CU-23 is five times higher compared with MF-4SC, the diffusion coefficients of lithium cations in MF-4SC membranes with their uniform distribution of sulfo groups and regular structure of channels were found to be comparable with Li^+^ diffusion coefficients in CU-23 in which the transport routes are formed by ionogenic clusters connected by narrow channels (see curves 3, 4 in Figure 49). The translational mobility of Li^+^ in terephthalic salt (PA-2) having a regular rod-like structure of channels was shown to be higher than in isophthalic salt (PA-1) with a globular packing of polymer chains (see curves 1, 2 in Figure 49).

#### 1.4.6. Diffusion of Saturated Monatomic Alcohols and Water–Alcohol Mixtures

Alcohol molecules’ transport through ion-exchange membranes is important for a water-alcohol separation processes development. Investigation of alcohol membrane transfer mechanism is also topical for methanol and ethanol fuel cell operation understanding. A lot of publications were devoted to diffusion of saturated monoatomic alcohols and their mixtures in Nafion [64,67,68,72] and MF-4SC membranes [47,51,73]. Diffusion of water-ethanol solutions through membranes based on mono- and disulfo-containing aromatic polyamides [51] and through composite polyacrylic acid-polysulfonate membranes [48] is studied.

**Figure 50:**
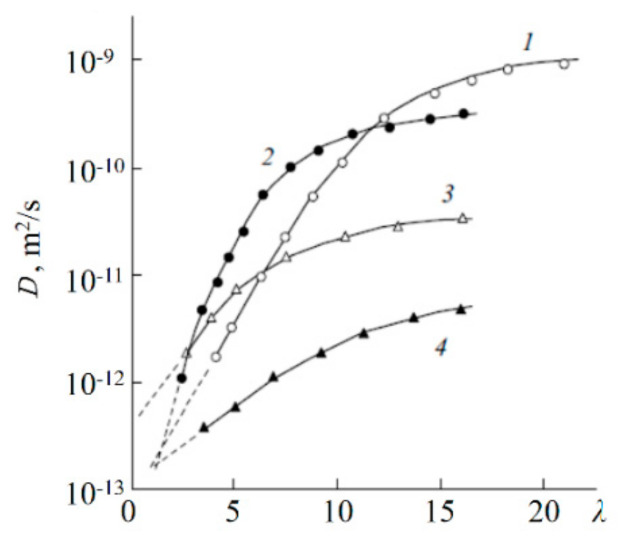
Effect of the diffusant content on the diffusion coefficients of water (1), methanol (2), ethanol (3), propanol (4) in the Li+-form of perfluorinated membrane MF-4SC m is the number of diffusant molecules per sulfonate group (adapted from [47]).

Alcohols’ molecules sorbing take place mainly in ionogenic channels of sulfonate MF-4SC membranes. Water, methanol, ethanol, and propanol self-diffusion coefficient dependences on diffusant content are shown in Figure 50. At maximum water and alcohol contents, self-diffusion coefficient of water molecules is more compared to alcohol molecules, but at low membrane diffusant occupation, water self-diffusion coefficient became less than alcohol ones.

**Figure 51:**
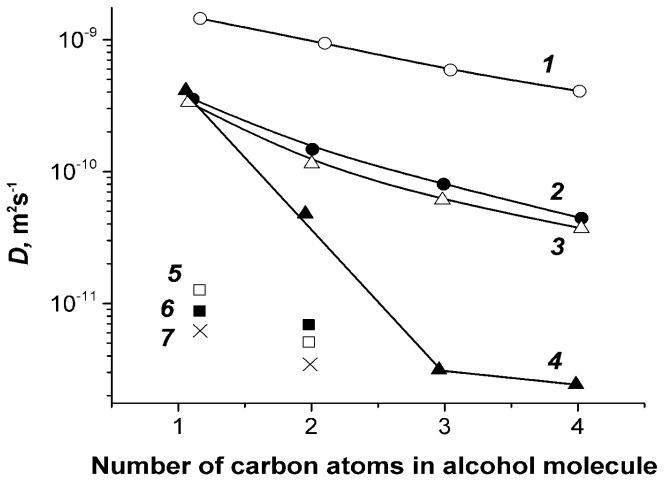
Diffusion coefficients of methanol, ethanol, propanol, and butanol in bulk liquids (1) and MF-4SC membranes in various ionic forms. Membrane ionic form: (2) Li^+^, (3) H^+^, (4) Na^+^, (5) Cs^+^, (6) Rb^+^, (7) K^+^ (adapted from [47]).

The diffusion coefficients and diffusion activation energies sharply depend on the ionic form of the membrane and the concentration dependences of diffusion coefficients of alcohols follow a threshold pattern (Figure 50 and Figure 51). Hence, it can be concluded that diffusion of alcohols in MF-4SC membranes is similar to water diffusion.

Translational transfer of alcohols and water molecules in MF-4SC membranes and monosulfo-containing polyamide films proceeds via the same ionogenic channels. For this reason, in these systems, the separation factors of water–alcohol mixtures are low [51]. Table 11 shows the partial diffusion coefficients of water and ethanol and the separation factors of their mixture in MF-4SC membranes, films of monosulfo-containing polyamide (PA-1) and disulfo-containing polyamide (PA-2), composite membranes based on polyacrylic acid and polysulfone (PAA-PSF). Apparently, for PA-2 and PAA-PSF, the separation factors are higher by several orders of magnitude, as compared with MF-4SC and PA-1. This is connected with the fact that in contrast to MF-4SC and PA-1, the water and ethanol molecules in PA-2 and PAA-PSF are located in different transport channels [48,51].

## 2. Conclusions

Nowadays, chemical power sources, based on sulfocation exchange membranes find wide application. The principal part of these systems is formed by a polymer ion exchange membrane that should have high ionic conductivity. The problem of revealing the mechanism of ion transport in these membranes becomes quite challenging. The last decade was characterized by active studies of the state and mobility of cations and molecules in ion-exchange membranes of various types by using the methods of NMR and pulsed field gradient NMR on various nuclei.

Therefore, the review of NMR technique applications is a problem of today. In this review paper the results of cation-exchange membrane investigation obtained by hetero nuclear high resolution NMR spectroscopy, NMR relaxation, pulsed field gradient NMR are discussed.

The main attention is given to interconnection of membrane diffusion channel nanostructure, cation hydration and water molecule and cation mobility in different spatial scales. NMR self-diffusion data are compared with ionic conductivity measurements.

The main parts of NMR investigations were carried out in Nafion (or Russian Nafion analog) MF-4SC sulfonate perfluorinated membranes. These membranes are most studied by different physical techniques and could be a model system for a wide set of ion-exchangers.

The comparison of local water molecule and Li^+^ cation mobility calculated from ^1^ H and ^7^ Li spin relaxation data with water and lithium cation self-diffusion coefficients measured by PFG NMR shows that macroscopic transfer is controlled by ion and molecular jumping near sulfonate groups. This result is conformed to Nafion channel structure model in Figure 3. Therefore, a cation hydration governs by ionic motion.

Hydration numbers of alkaline and alkaline–earth metal cations were calculated from water molecule ^1^H chemical shift temperature dependences. For Li^+^, Na^+^, Cs^+^ counter ions, the relative part of contact pairs cation-charge group dependently on humidity was measured by ^7^ Li, ^23^ Na,^133^ Cs NMR. Some conclusions about membrane selectivity mechanism to these ions were proposed.

It was definitely shown that in sulfonate cation–exchangers in acid ionic form the least hydration number is equal two and at low water content hydrated cation [H_5_O_2_]^+^ is formed.

In opposite to conception based on DSC data about water freezing in membranes below 0 °C it was shown that amount of mobile water molecules does not change at temperature variation in spite of DSC peak observing. On the basic of ^1^ H spin–relaxation data it was supposed that at freezing temperature water molecules form mobile associates, but not ice phase. This assumption explains water and cation self-diffusion and ion conductivity temperature dependences.

A comparison of ion conductivity calculated from cation self-diffusion coefficients with experimental values confirms the cluster-channel structural model for membranes based on sulfonated polystyrene (Figure 1) and channel structural model for Nafion membranes (Figure 3).

From our opinion these NMR results give opportunity to understand mechanism of ionic and molecular transport in ion-exchange membranes more deeply.

## 3. Biological Systems: Protein and Cell Membranes

**Figure 52:**
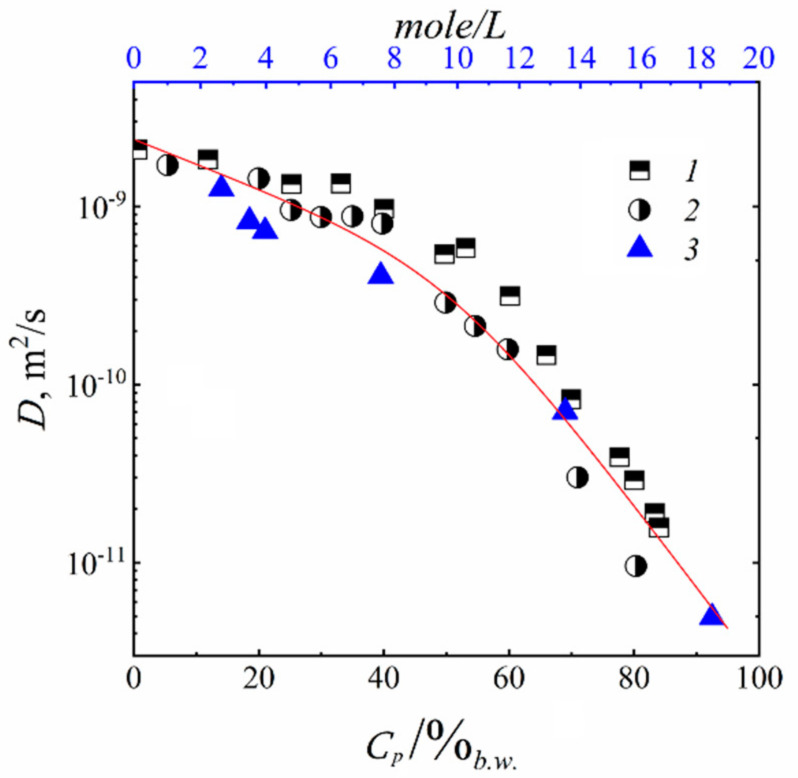
Water self-diffusion coefficients dependences on concentration (water content) in (1) bovine serum albumin, (2) gelatin, and (3) perfluorinated MS-4SC membrane in acidic form (adapted from [55]).

Another subject of our paper is biological systems: protein and red blood cells. In Figure 52 the dependence of water self-diffusion coefficients on gelatin and bovine serum albumin concentration in aqueous solution is shown (black). This dependence is similar to the dependence of water self-diffusion coefficient on water content in ion exchangers (blue in Figure 52). It may be proposed that water behavior in ion exchange membrane ionogenic channels and in protein ionic channels has some analogy. The forming polymer structure from macromolecules also may be similar to synthetic and biological polymers.

### 3.1. Protein Association

At the present time, the method of nuclear magnetic resonance with a pulsed field gradient magnetic (PFG NMR) is applied to study the features of translational mobility of a wide variety of complex molecular systems. Particular attention is paid to study the structure, dynamics, and functional (protein–protein) interactions of biomolecules. Note that determination of the geometric properties of molecules from the measured values of the self-diffusion coefficients using the Stokes–Einstein relation is only a part of the capabilities of the NMR method.

The interaction of proteins with other macromolecules or small partner molecules plays an important role in most biological processes [120]. Often, such interactions are manifested in a conformational exchange that occurs on a much faster time scale than most experimental biophysical methods [121]. NMR spectroscopy techniques in combination with pulsed magnetic field gradient NMR have a unique ability to extract information about these interactions and are used more often to investigate protein systems of increasing complexity, including proteins with an internally disordered structure (IDP). A characteristic feature of such proteins is that sample an ensemble of rapidly interconverting alternative conformations ranging from random coils to more structured conformations with secondary structure and residual tertiary structure elements. [122]. At the same time, IDP form well-defined complexes when interacting with a partner [123].

Another feature of PGF NMR method is that the translational mobility of the molecule under interest is registered directly existing at its native conditions without a need for any labeling. In addition, there is also no need for continuous calibrating and comparing with reference samples, when one analyzes PFG NMR data.

Besides the structural and geometric parameters of the molecules, PFG NMR investigations can afford fundamental insights into molecular mechanisms underlying the protein functionality, including conductivity of ion channels of membrane proteins [124,125], dynamics, and binding of intact ligands [126,127]. The effect of the bicells association with molecules such as proteins, peptides, detergents, or drugs can not only be detected with PFG NMR, but also quantitatively characterized based on its results, since the method provides information on the spectrum of self-diffusion coefficients for all components of the molecular system.

The topology features of the macromolecule, which determine its ability to change its conformation to a greater or lesser extent in a particular solvent [128,129], significantly affect the nature of the translational mobility of macromolecules. This fact was confirmed by the results of studying the self-diffusion of globular proteins in aqueous suspensions [130].

**Figure 53:**
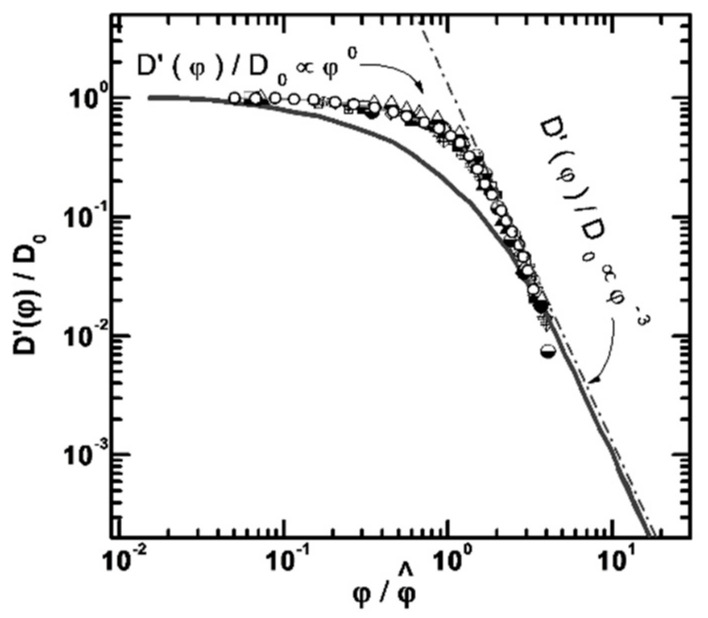
Generalized concentration dependence on concentration of the self-diffusion coefficients of globular proteins molecules [130]. The figure shows the experimental values for BSA (pH = 4.8–5.2), myoglobin (pH = 6.8–7.2), lysozyme (pH = 2.9–3.0 and pH = 7.4–7.8), barstar (pH = 8.0–8.2). The solid line is the generalized self-diffusion coefficients concentration dependence of the flexible-chain polymers [132]. $\hat{\varphi }$–critical concentration, found as the intersection of the asymptote with zero slope *ϕ*^−3^, drawn to the experimental concentration dependences of the self-diffusion coefficients of proteins.

When immersed in water, some protein chains assume a rigid globular conformation [131]. Self-diffusion of globular proteins in aqueous solutions in [130] was studied by PFG NMR in a fairly wide range of concentrations of macromolecules at different pH-values of the medium and at different temperatures. In particular, aqueous suspensions of myoglobin, bovine serum albumin, barstar, and lysozyme were studied. Using the method of constructing generalized concentration dependences of self-diffusion coefficient of macromolecules [132], authors of the paper [130] managed to obtain a generalized concentration dependence for proteins, which, in turn, gave certain grounds to speculate about the existence of some general regularities of self-diffusion of globular proteins in aqueous suspensions (Figure 53).

It was shown [130] that the generalized concentration dependence of the self-diffusion coefficients of proteins, like the universal concentration dependence of the self-diffusion coefficients of linear flexible-chain polymers, approaches the characteristic asymptotes with *ϕ*^−0^ in the limit of dilute solutions and with *ϕ*^−3^ in the region of concentrated solutions.

However, in the intermediate concentration range, the obtained generalized dependence of self-diffusion coefficients for proteins differs significantly from the analogous curve for polymers. In [130], this difference is explained by the peculiarities of the dynamic behavior of proteins, which are due to the specific globular conformation of polypeptide chains. At the same time, a comparison of the diffusion behavior of globular proteins and rigid Brownian particles (see, for example, [133,134]) showed that some differences are observed only in the region of concentrated solutions, at $\varphi \gg \hat{\varphi }$ [130].

Thus, in the case of aqueous solutions of globular proteins, the study of self-diffusion of macromolecules by PFG NMR, the construction of a generalized concentration dependence of the self-diffusion coefficients of proteins, and a comparison of this dependence with a similar curve for flexible-chain polymers made it possible to qualitatively describe the features of the translational mobility of polypeptide chains and to characterize the structure of globules in the studied solutions.

At this point in time, in addition to structured globular proteins, studies of proteins with an internally disordered structure, which are characterized by high flexibility and the presence of disordered regions of the polypeptide chain, are relevant, and at the same time retain their functionally active state.

Many studies have shown [135,136] that IDP is capable of forming ordered aggregates—amyloid fibrils. Such formations can be the cause of many serious diseases, such as neurodegenerative diseases of Alzheimer’s and Parkinson’s [137,138].

It should be noted that it is necessary to be able to adequately describe this “family” of proteins and to correctly correlate the physical properties of IDP with their function in order to better understand the structural and dynamic features of such proteins as a whole. For the study of such systems, the NMR method has a wide range of possibilities and is, in fact, the most informative physicochemical method for studying protein molecules.

**Figure 54:**
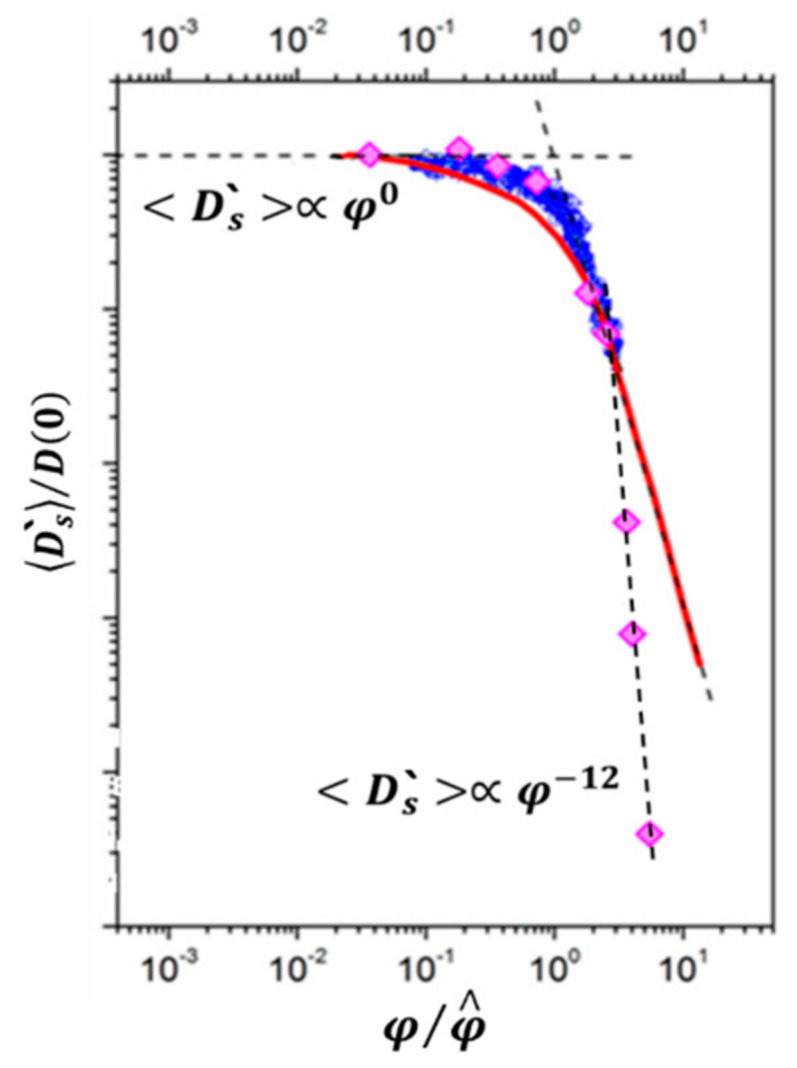
Concentration dependence of the diffusion coefficients of α-casein [139], globular proteins [130], and linear flexible polymers [132]. The master curves for globular proteins (blue experimental points) and flexible linear polymers (red solid line) are shown. The asymptotes with the slopes of *ϕ*^−0^, *ϕ*^−3^, and *ϕ*^−12^ are indicated. Solid magenta diamonds represent the concentration dependence of the diffusion coefficient of α-casein *< D >*/*D*_0_ normalized by $\hat{\varphi }$.

Melnikova et al. [139] investigated the dependence of the translational diffusion of α-casein, a typical representative of intrinsically disordered proteins, on the protein concentration, diffusion time, and storage time of the sample using the PFG NMR method. In this work, the authors compared the concentration dependence of the self-diffusion coefficient of a representative of IDP with generalized curves as a first step towards understanding the features of IDP diffusion in a wide range of experimental conditions. The dependence of α-casein self-diffusion coefficient *< D >* on protein concentration, expressed as a volume fraction *φ*, is shown in Figure 54 (purple diamonds).

As can be seen from the Figure 54, the concentration dependence of the self-diffusion coefficients of α-casein from the side of low concentrations shows, at first, the same tendency as globular proteins. However, in concentrated solutions it crosses the curve for linear flexible-chain macromolecules and reaches the asymptotic $<D_{s}^{’}>\propto \varphi^{-12}$.

The authors of the work [139], found that it is the self-organization effect, which manifests itself in the α-casein/water system above a certain critical concentration, that is the reason for the anomalously strong concentration dependence of the average values of the self-diffusion coefficients of α-casein molecules in the region of high concentrations. Moreover, the obtained concentration dependence does not coincide with any of the known generalized concentration dependences of the average self-diffusion coefficients previously established for linear flexible-chain polymers, as well as for dendrimers and globular proteins.

**Figure 55:**
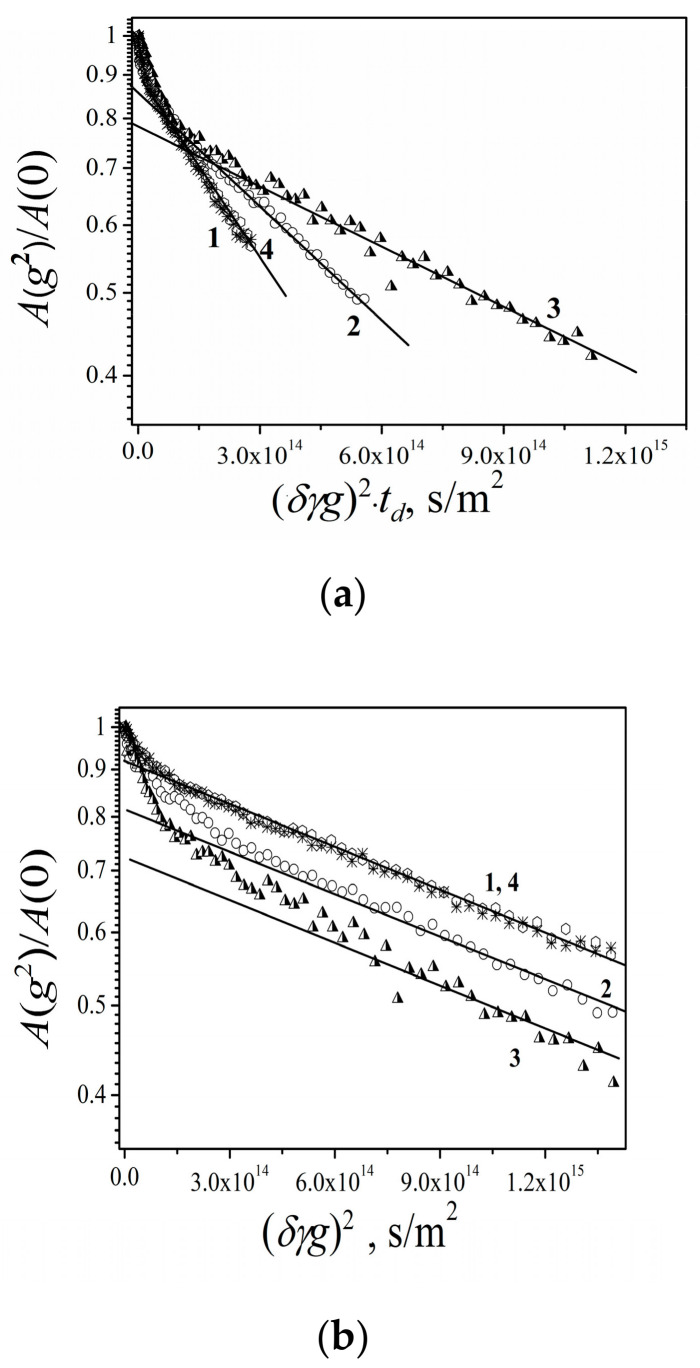
Dependence of the diffusion attenuation of spin–echo signal in solutions of α-casein on diffusion time [139]. Same diffusion attenuations are plotted using different coordinates: [43,140] (**a**) as a function of (*γδg*) ^2^*t_d_* and (**b**) as a funcTable 2. Diffusion attenuations are shown for 15% α-casein solution. Curves 1–3 correspond to diffusion attenuations collected at diffusion times 200, 400, and 800 ms, respectively. Curve 4 is a control experiment. The diffusion attenuation shown by curve 4 was collected at 200 ms after the completion of the experiments carried out at different values of *t_d_*.

To establish the effect of self-organization, the authors of the investigation analyzed the dependences of the forms of diffusion decays of the spin echo signal on the diffusion time. In regions of relatively high concentrations, anomalous behavior of the self-diffusion coefficient was found. Figure 55 shows diffusion attenuations for the 15% α-casein solution, acquired at *t_d_* values of 200, 400, and 800 ms.

The graphs are presented in conventional coordinates (Figure 55a) and in coordinates corresponding to testing the fully limited diffusion mode [140,141] (Figure 55b). As can be seen from Figure 55a, with an increase in the diffusion time for a part of protein molecules characterized by small values of self-diffusion coefficient, the slope of diffusion attenuations, which corresponds to a decrease in the values of the self-diffusion coefficients for this part of molecules with an increase in the diffusion time *t_d_*. Figure 55b shows the same diffusion attenuations, but in coordinates in which fully limited diffusion mode can be easily tested. In these coordinates, it is clearly seen that the slope of the diffusion attenuation component with the lowest self-diffusion coefficient remains unchanged for all *t_d_* values, as shown by the dashed lines in Figure 55b. This proves that the self-diffusion coefficient *D*_min_ is inversely proportional to the diffusion time, and the root-mean-square displacement of α-casein molecules remains constant < *r*^2^ > ~ *t_d_*_0_ as follows from the Equation (A4).

The presence of such a dependence allows us to assert that in concentrated solutions of α-casein, processes are observed that are very similar to the processes of gelation. Similar effects were observed in [43], especially in gelatin gels [140], as well as in radiation cross-linked polybutadienes [142].

The size of constraints calculated by (A4) is equal to $\sqrt{r^{2}}\approx 50\;\pm \;5\;\mathrm{nm}$. The obtained value significantly exceeds the size of the α-casein molecule itself, the hydrodynamic radius of which is in the range of 2.2–3.2 nm [143]. Thus, the effect of limited diffusion of α-casein can only be associated with the fact that several α-casein molecules interact with each other to form a supramolecular structure similar to a three-dimensional gel network. In this case, interactions through the hydrophobic regions of the protein, hydrogen, ionic and other non-covalent bonds can act as intermolecular bonds [144,145,146].

In addition, the authors of the article [139] demonstrated that in the studied concentrated solution there are two sub-assemblies of α-casein molecules with different characteristics of translational mobility: by the values of the self-diffusion coefficients and by their dependence on the diffusion time. Moreover, it was possible to show that a state of dynamic equilibrium is realized between the indicated sub-assemblies of α-casein molecules. In other words, molecular exchange occurs between α-casein molecules in the gel state and free molecules with a characteristic lifetime of α-casein molecules in the gel phase 3.5 ± 0.4 s. In this case, the stationary value of the fraction of α-casein molecules in the gel state is estimated at 0.93 ± 0.01.

It should be noted that as we know in the studies of systems with gelation by the PFG NMR (see, for example, [43]), no signs of exchange between free polymer molecules and in a gel state were found.

It should be recognized that the amount of experimental material concerning the transport properties of IDP in solutions and mixtures remains insufficient. As shown in a review of experimental results of studying the self-diffusion of polymers and globular proteins in solutions, NMR diffusion (PFG NMR) is one of the most effective methods for studying the structural and dynamic properties of molecules in condensed systems.

### 3.2. Water and Biological Active Substances Self-Diffusion in Red Blood Cells

The transport of substances into biological cells is carried out in two main ways: active and passive. Active transport is the transfer of a substance through a biomembrane, flowing against a concentration gradient (from a region of low concentration to a region of high concentration), i.e., with the expenditure of free energy.

Due to passive transport, substances are transferred along a concentration gradient from a high concentration region to a low concentration region without energy consumption (diffusion, osmosis). There is a wide variety of diffusion transfer methods: diffusion of fat-soluble substances through the lipid part of the membrane; transport of hydrophilic substances through the pores formed by membrane lipids and proteins; facilitated diffusion with the participation of special carrier molecules. In the lipid phase of the membrane, non-polar substances are readily soluble: organic and fatty acids, esters, since they have an increased affinity for the lipid bilayer.

**Figure 56:**
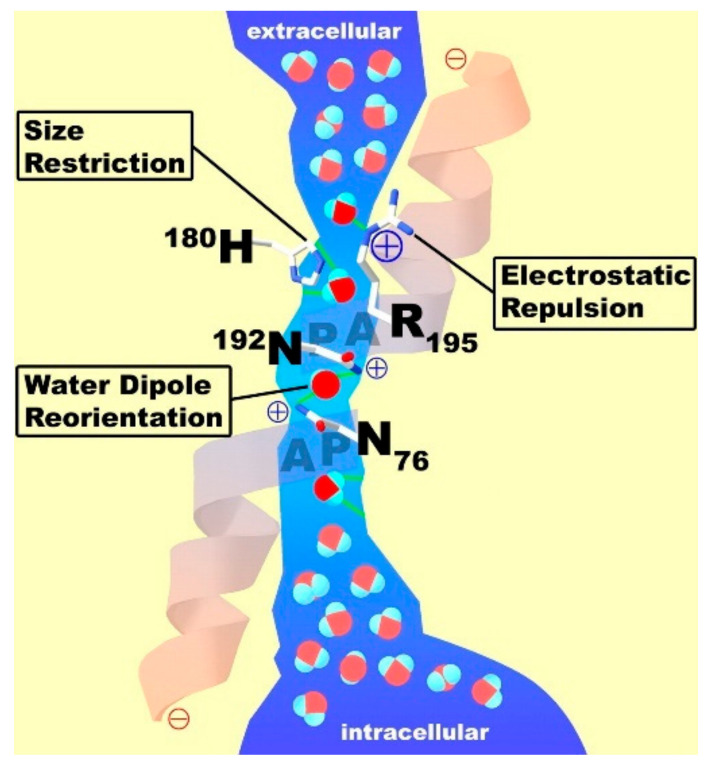
Schematic structure of the aquaporin channel. The fixed positive charge on the side chains of Arg-195 (R195) and His-180 (H180) create a barrier to cations, including the protonated water (H_3_O^+^). The hydrogen bonds of the water chain adjacent to Arg-195 are broken, thereby preventing the formation of proton conductivity [150]. Reprinted with permission from [150]. Copyright 2002 American Society for Clinical Investigation.

At the same time, polar substances (inorganic salts, sugars) poorly pass through the lipid bilayer. However, for water, the value of permeability is about 10^−6^ m/s, which is quite large for a polar substance insoluble in lipids [147]. An explanation for this phenomenon was found with the discovery of aquaporins—integral membrane proteins that form pores in cell membranes, through which water molecules are selectively passed, allowing it to enter and leave the cell, while at the same time ions and other soluble substances are not allowed through (Figure 56) [148,149]. That is, there are two ways for water to enter the cell: through the lipid bilayer and through aquaporins. The schematic movement of water through the channel formed by aquaporin is shown in Figure 56 [150]. The aquaporin channel is shaped like an hourglass. Bulk water is located in the extracellular vestibule and the intracellular vestibule of the canal. Further, the vestibule turns into a narrow tunnel with a width of up to 2.8 Å, which is enough for the passage of one water molecule only, while big molecules or hydrated ions are unable to pass through it. It has now been ascertained that some aquaporins also allow other solutes to pass through (glycerin, urea, ammonia, etc.) [151].

Water molecules are very important in the functioning of biological cells. Water is a solvent for many substances; acts as a medium for chemical reactions and participates in them (hydrolysis); maintains the shape of the cell and participates in its thermoregulation. Therefore, it is necessary to study the characteristics of water exchange in biological cells. Red blood cells (RBCs, erythrocyte) are of interest for such studies, since they are the main component of blood and play a key role in the formation of rheological parameters of blood. The erythrocyte membrane also has general principles of the organization of biological membranes.

#### 3.2.1. RBC Water Permeability Studied by Paramagnetic Doping Technique

A number of papers are devoted to the permeability study of humans, birds and animals RBCs membranes for water molecules using various techniques [152,153,154,155,156]. Studies of water exchange in RBCs have not lost their relevance for more than 50 years. So, at the beginning, the look of the researchers was aimed at establishing the pathways for transporting water into the cell, and the presence of water channels was only assumed [152,153]. The discovery of aquaporins has brought some clarity to this issue. The search for methods for assessing the permeability of cell walls is still important [154,155,156]. This is due to the peculiarities of obtaining RBCs, the short time of their “life” outside the organism, which is due to their rapid response to changes in the environment. The most methods provide only indirect information about metabolic processes (including the water permeability of membranes).

In the field of studying the permeability of RBCs membranes for water molecules, NMR techniques are widely used [156,157,158,159,160,161]. In practice, two methods are used to assess the permeability of biological membranes: paramagnetic doping NMR technique and pulsed field gradient NMR technique.

In the first case, paramagnetic salt solutions are introduced into the extracellular region of the cell suspension, which leads to a decrease in the relaxation times of the protons of water molecules. The exchange rate *k* = 1/*τ* is calculated as the difference between the rates of spin–spin relaxation of protons in intra- and extracellular water, where *τ* is intracellular water lifetime. Permeability is calculated by the formula: $P=k\frac{V}{S}$, where *V/S* is the ratio of cell volume to its surface area [157].

**Figure 57:**
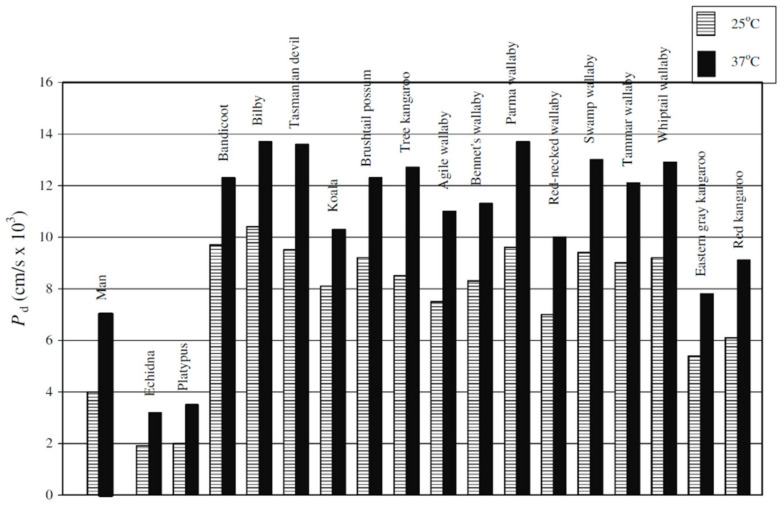
Values of the permeability *P* of RBCs of humans and some laboratory animals and pets for water at 25 and 37 °C. Reprinted with permission from [156]. Copyright 2012 Springer Nature.

This method is widely used in the works of Benga and Kuchel with colleagues [156]. The authors have evaluated the permeability of RBCs membranes for water in humans and more than 30 animal species. In Figure 57a, diagram of the values of the permeability *P* of RBCs for water in humans and some animal species at temperatures of 25 and 37 °C is shown. The authors put forward an interesting hypothesis about the relationship between the permeability of the RBCs membrane and the physical activity of the species. As shown by the results of NMR experiments, the permeability of RBCs to water is higher in species with greater physical activity, with a higher metabolic rate, or a higher average blood circulation rate.

**Figure 58:**
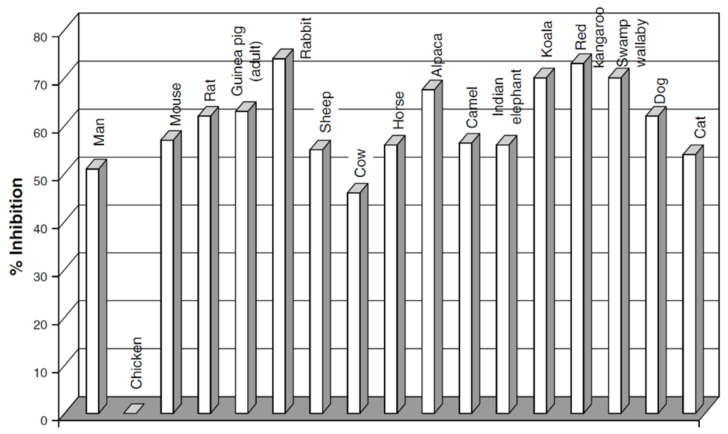
Inhibition of the permeability of RBCs in humans and some laboratory animals and pets for water by PCMBS. Reprinted with permission from [156]. Copyright 2012 Springer Nature.

In order to establish the mechanism of permeability, the permeability of erythrocytes was measured when protein channels in membranes are blocked. The inhibition of the permeability of the RBCs membrane for water molecules caused by insertion of p-Chloromercuribenzene sulfonate (PCMBS), which blocks the transfer of water through water protein channels, was calculated. A diagram showing the percentage of inhibition of permeability for different types of RBC is shown in Figure 58. The main result of the work is the correlation of the RBCs water permeability with the molecular basis of the process. It has been shown that the permeability of erythrocytes for water, inhibited by PCMBS, corresponds to the water transported through the membrane by water protein channels. A low percentage of inhibition indicates the absence of water protein channels in the RBCs membrane. This is observed for chicken red blood cells, for example. For human RBCs ~50% of the membrane’s permeability to water is due to water protein channels. Thus, the percentage inhibition of permeability is related to the number of blocked water protein channels and differs from species to species.

Despite the elegance of the paramagnetic doping NMR method for estimating the water permeability of RBCs, the presence of paramagnetic ions can affect the actual metabolic process, that was discussed in the works [161,162,163]. In addition, the obtained values of the permeability of RBCs are calculated from the indirect parameters of molecular diffusion.

From this point of view, pulsed field gradient NMR (PFG NMR) is preferable. This technique allows a direct quantitative estimate of molecular diffusion without a destructive effect on biological systems [160,164,165,166,167]. The originality of the method lies in the ability to measure the partial self-diffusion coefficients and the relative amounts (populations) of diffusant molecules in heterogeneous systems, which include biological cells [82,83,168,169,170].

Below we have discussed the self-diffusion of water molecules, lateral diffusion and self-diffusion of pentasubstituted fullerene derivatives C_60_ (with adducts of 3-mercaptopropanesulfonic acid C_60_[S(CH_2_)_3_SO_3_Na]_5_H (**1**), mercaptopropionic acid C_60_[S(CH_2_)_2_COOK]_5_H (**2**), proline C_60_[N(CH_2_)_3_CHCOOK]_5_Cl (**3**)) in a RBCs suspensions investigated by PFG NMR [171,172,173].

To measure the self-diffusion coefficients of diffusant molecules in a suspension of RBCs, the “stimulated echo” pulsed sequence was applied. The details of PFG NMR technique are given in supplementary.

#### 3.2.2. Self-Diffusion of Water Molecules in a RBCs Suspension

**Figure 59:**
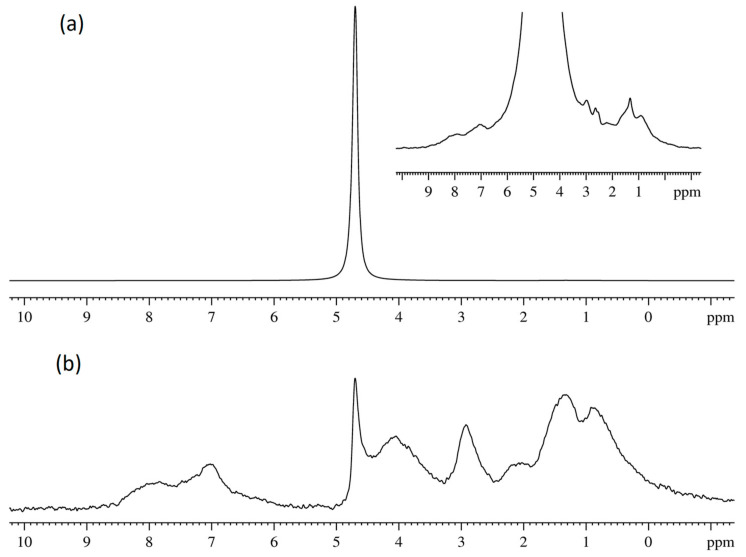
The RBCs suspension ^1^ H spectra with suppression of water signal—pulsed field gradient amplitude g = 0.375 T/m (**a**) and g = 10.5 T/m (**b**). 0–1.70 ppm: (CH2)*n* and CH3 groups of lipids; 6–9 ppm: protein component (protons of aromatic rings and NH and OH groups. Reprinted with permission from [171]. Copyright 2016 Springer Nature.

Figure 59 shows the proton spectra of a suspension of RBCs at different values of pulsed field gradient amplitudes.

**Figure 60:**
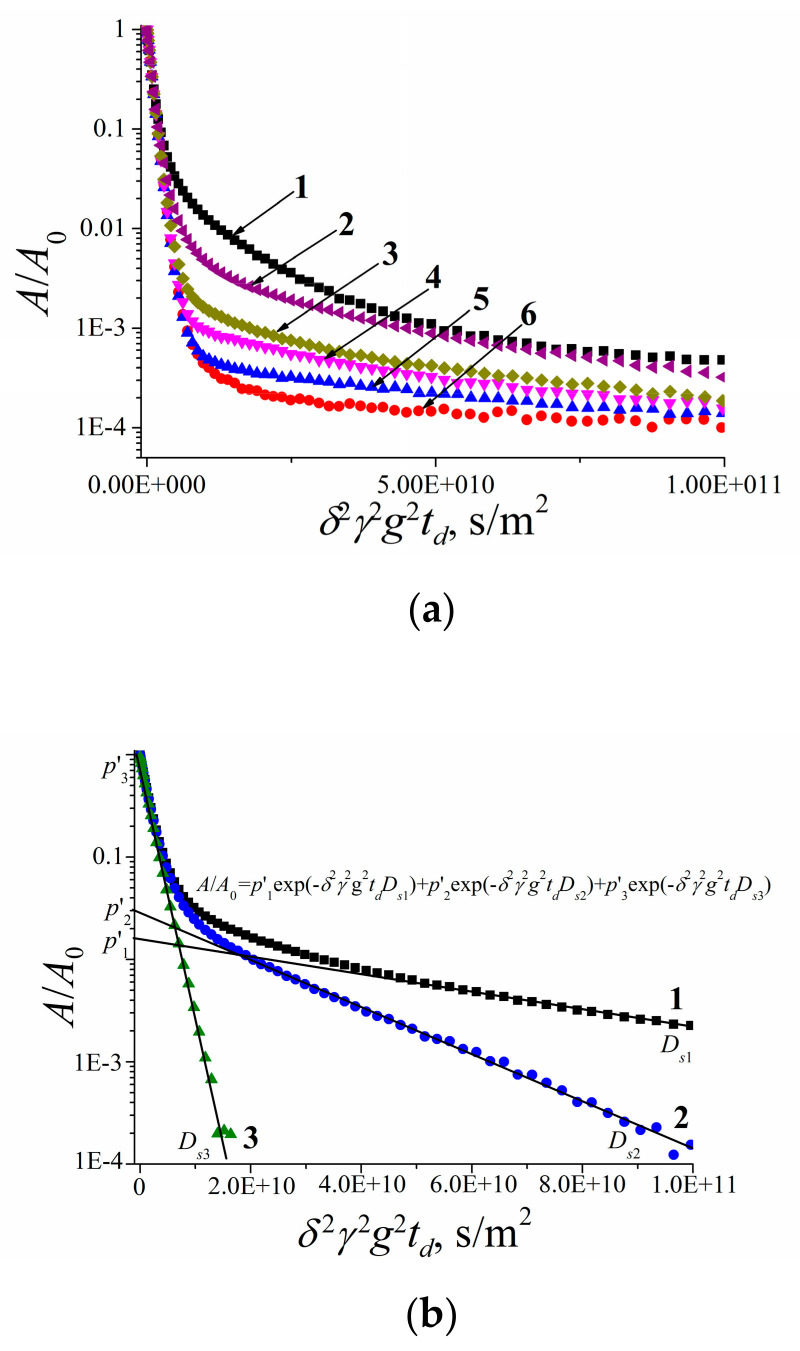
(**a**) The diffusion decays of water molecules in RBCs measured at various *t_d_* (1—10 ms, 2—20 ms, 3—50 ms, 4—100 ms, 5—250 ms, 6—500 ms) and *t* = 35 °C; (**b**) the procedure of decomposition of the original diffusion decay on three exponential components according to Equation A1 is shown: *D_s_*_3_ = 0.60·10^−9^ m^2^/s, *p*_3_′ = 0.947; *D_s_*_2_ = 0.76·10^−10^ m^2^/s, *p*_2_′ = 0.045; m^2^/s; *D_s_*_1_ = 1.37·10^−11^ m^2^/s, *p*_1_′ = 0.008 (1–original diffusion decay, 2–1st residual, 3–2nd residual). Reprinted with permission from [171]. Copyright 2016 Springer Nature.

Figure 60a shows the diffusion decays of protons of water molecules in RBCs suspension at different diffusion times *t_d_* [26]. This diffusion decays are decomposed into three exponential components approximated well (in four orders of magnitudes) by Equation (A1), as it is illustrated in Figure 60b. In Figure 60b capture the partial self-diffusion coefficients *D_s_*_1_, *D_s_*_2_, *D_s_*_3_ and populations *p*_1_, *p*_2_, *p*_3_ are indicated.

**Figure 61:**
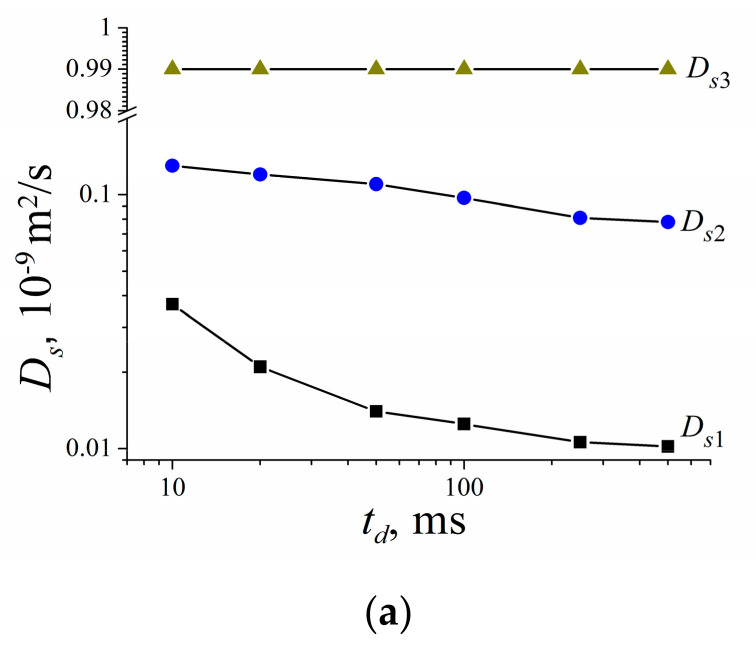

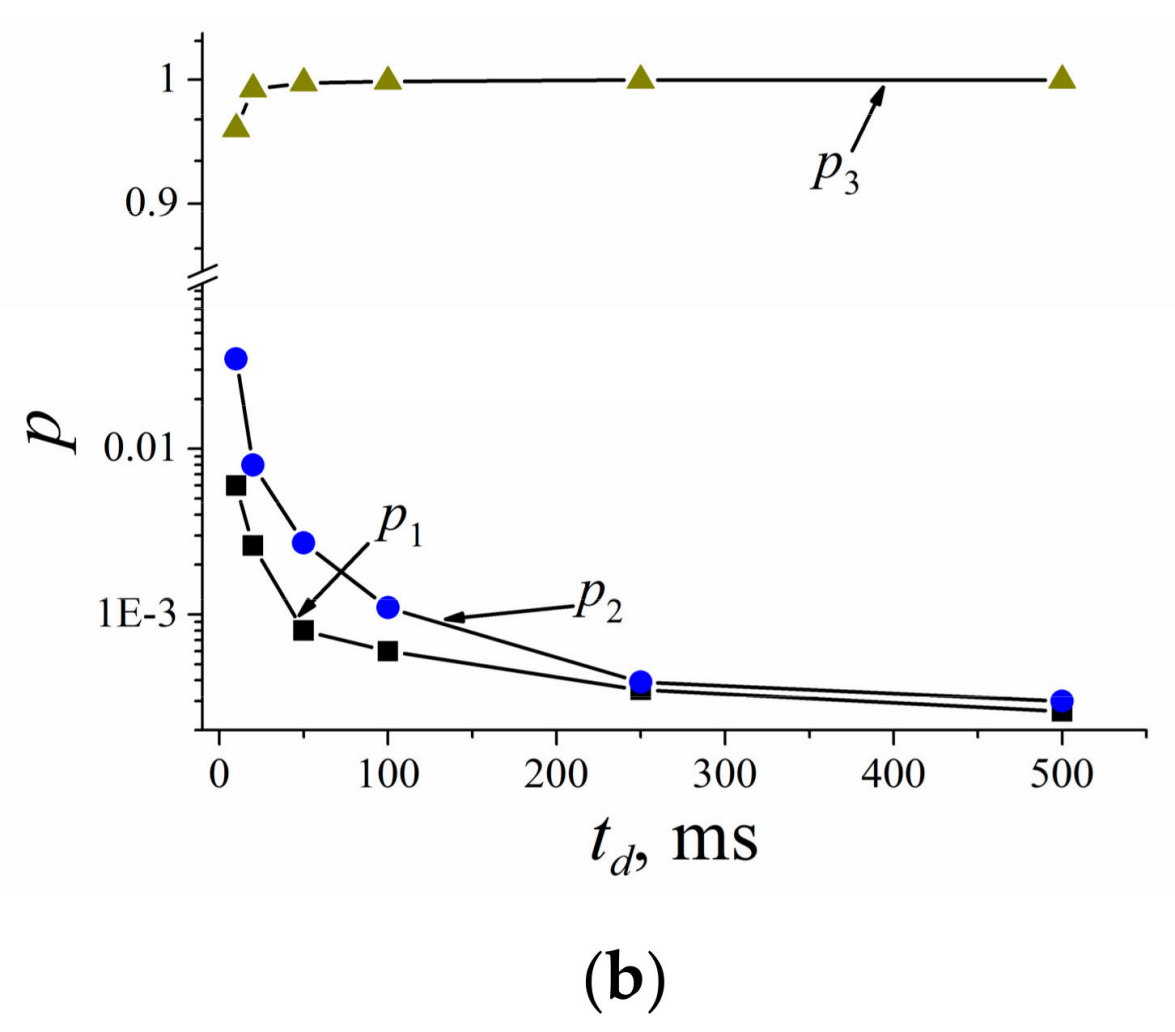
The water self-diffusion coefficient *D_s1_, D_s2_*, and *D_s3_* dependences on the diffusion time *t_d_* at 35 °C (**a**). The population *p_1_, p_2_,* and *p_3_* dependences on the diffusion time *t_d_* at 35 °C (**b**) Reprinted with permission from [171]. Copyright 2016 Springer Nature.

Partial self-diffusion coefficients and populations dependences on the diffusion time *t_d_* are given in Figure 61. With *t_d_* increasing self-diffusion coefficients *D_s_*_1_ and *D_s_*_2_ are decreased, while a *D_s_*_3_ is diffusion time independent. The *D_s3_* value closes to the self-diffusion coefficient of bulk water. The population *p_1_* increases but *p*_2_*, p*_3_ decrease with increasing of *t_d_*.

These three types of water in the RBCs suspension are intracellular, extracellular, and bulk water. Similar conclusions were obtained for water in other biological systems (chlorella and yeast) [82,83,170]. In the following discussion, the main attention is paid to intracellular water, which behavior is characterized by *D_s_*_1_ and *p*_1_.

**Figure A2:**
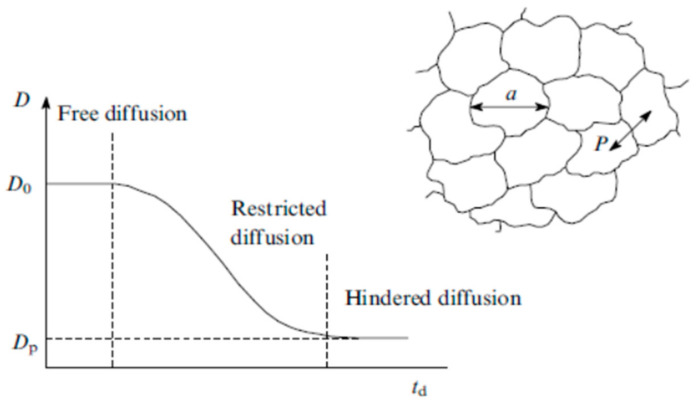
Illustration of the dependence of the apparent diffusion coefficient *D* on diffusion time *t_d_* for the case of restricted diffusion. *D*_0_ is the free diffusion coefficient, *D_p_* is the restricted diffusion coefficient, *a* is the size of restriction region (pore or cell diameter), *a = (6Dt_d_)*^1/2^; *P* is the permeability of pore (cell) [174].

The *D_s_*_1_(*t_d_*) decreasing with increasing of diffusion time indicates the limited nature of water self-diffusion in erythrocytes, which corresponds to region of restricted diffusion in Figure A2 [174,175].

**Figure 62:**
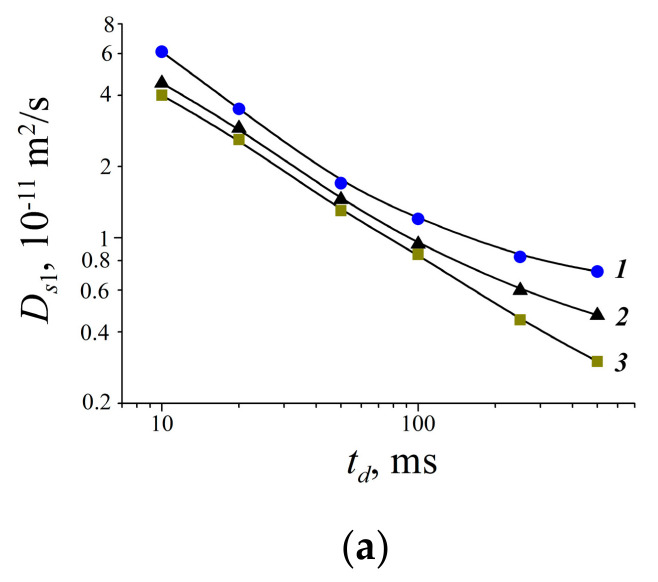

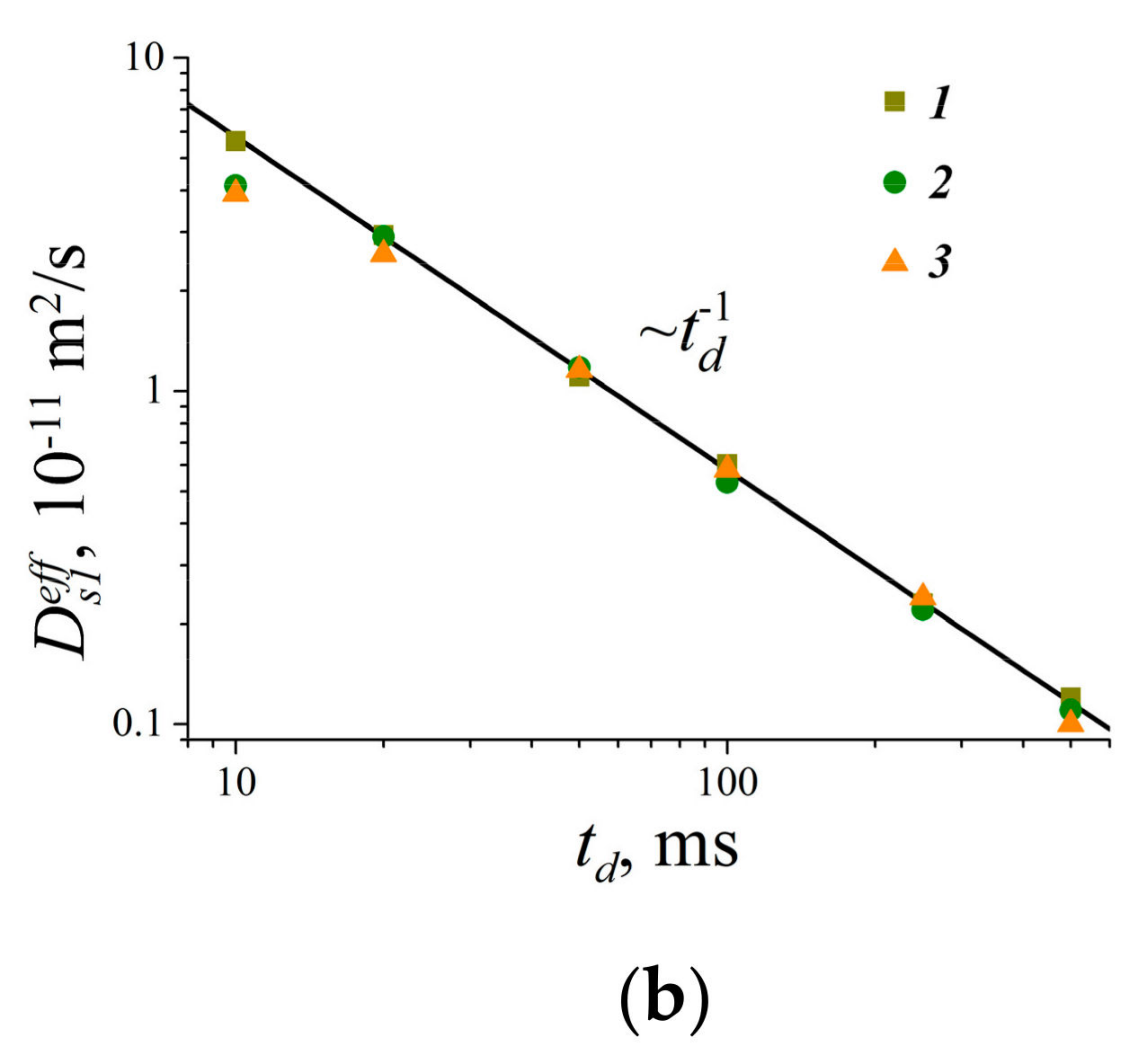
The experimental dependence of self-diffusion coefficients *D_s_*(*t_d_*) (**a**) *T* = 35 (1), 20 (2), and 5 (3) °C and effective self-diffusion coefficients *D_s_^eff^*(*t_d_*) calculated from Equation (A5) (**b**) *T* = 35 (1), 25 (2), and 10 (3) °C on the diffusion time *t_d_* for intracellular water molecules in the RBCs. Reprinted with permission from [171]. Copyright 2016 Springer Nature.

Figure 62a shows the dependences of the self-diffusion coefficient of intracellular water molecules *D_s_*_1_ on the diffusion time at temperatures of 5, 20, and 35 °C. As it can be seen from the figure, the dependence *D_s_*_1_(*t_d_*) is flatter compared to *t_d_*^−1^. The effective self-diffusion coefficients calculated from Equation A6 are shown in Figure 62b. Parameters *D*_0_ and *D_p_* were: at 10 °C *D*_0_ = 2.7∙10^−9^ m^2^/s, *D_p_* = 2.5∙10^−12^ m^2^/s; at 25 °C *D*_0_ = 2.7∙10^−9^ m^2^/s, *D_p_* = 4.5∙10^−12^ m^2^/s; at 35 °C *D*_0_ = 2.7∙10^−9^ m^2^/s, *D_p_* = 6.0∙10^−12^ m^2^/s.

If the values of *D*_0_*, D_p_*, and the pore size *a* are known the value of the permeability *P* of the pore wall can be calculated using the Equation (A5) [176], where *P* is the permeability of the pore wall, *a* is the pore size. This equation was used to assess the permeability of the cell walls of the chlorella [83], yeast [82], and roots of corn [175].

For erythrocytes, the diffusion restriction size *a*, calculated from Equation (A4), does not depend on temperature, and its value was 2.1 μm. The value of the permeability *P* of the cell membrane of mouse erythrocytes, calculated in accordance with Equation (A5), varies from 0.3·10^−5^ m/s to 0.5·10^−5^ m/s with an increasing temperature from 5 to 35 °C

**Figure 63:**
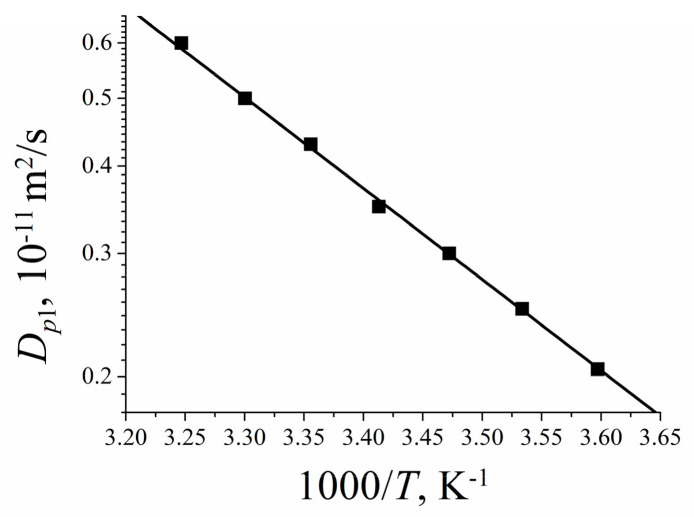
A temperature dependence of the hindered water self-diffusion coefficient *D_p_*. The solid straight line is the Arrhenius equation approximation. The activation energy is 24.1 ± 1.9 kJ/mol. Reprinted with permission from [171]. Copyright 2016 Springer Nature.

The hindered water self-diffusion coefficient *D_p_*, which characterized permeability was calculated from Equation (A5). The temperature dependence of *D_p_* is Arrhenius type (Figure 63).

As it follows from Equation (A5), under the condition *D*_0_ *>> D_p_*, this dependence actually reflects the dependence of the cell wall permeability *P* on the temperature *t*. The activation energy of self-diffusion was *E_D_* = 24.1 ± 1.9 kJ/mol.

Due to the cell wall permeability, a molecular exchange between intracellular and extracellular water takes place. To estimate the residence time of water molecule inside the cell and the corresponding exchange constant k, a two-component model of molecular exchange is used, which implies an exponential distribution function of the residence time. As it follows from this model, the relative part of water molecules in the cell (population *p*_1_) dependence on diffusion time is (10) [43,176,177]:

(10) $$p_{1}\left(t_{d}\right)\;=\;p_{1}\left(0\right)\;exp\;\left(-\frac{t_{d}}{\tau }\right)$$

where *τ* is the water molecules residence time in the cell.

**Figure 64:**
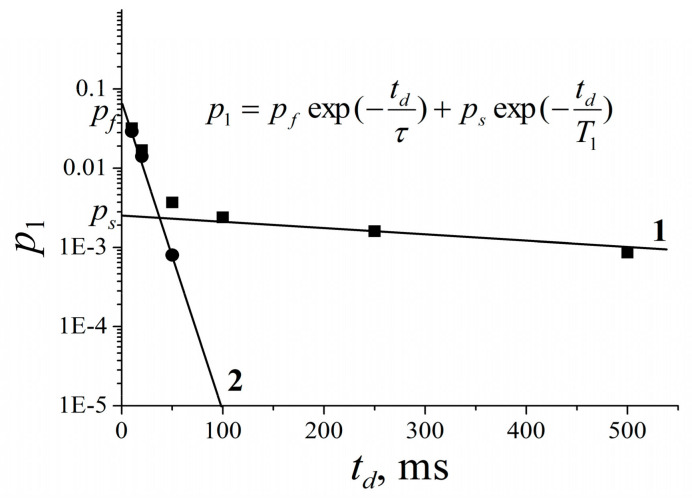
(1) the dependence of intracellular water population *p*_1_(*t_d_*) on diffusion time *t_d_*. (2) The dependence *p*_1_(*t_d_*) after subtraction of spin–lattice relaxation part. Temperature is 35 °C. Reprinted with permission from [171]. Copyright 2016 Springer Nature.

Another reason of population *p*_1_ reducing is a spin–lattice relaxation. Therefore, the *p*_1_(*t_d_*) is a biexpontial shape [178]:

(11) $$p_{1}\;=\;p_{f}\;exp\left(-\frac{t_{d}}{\tau }\right)+p_{s}\;exp\left(-\frac{t_{d}}{T_{1}}\right)$$

where *τ* is the lifetime of the molecule; *T*_1_ is a spin–lattice relaxation time, which is ≈600 ms; *p_s_, p_f_* are the weights of a slow and fast components, respectively. Figure 64 shows the dependence *p_1_(t_d_)* and its decomposition into components in accordance with Equation (11). The value of the lifetime τ of the molecule inside the cell was 20 ± 2 ms at 30 °C.

#### 3.2.3. Lateral Diffusion in the RBCc Membrane

To study the self-diffusion of lipids in the RBCs membrane, we analyzed the diffusion decay of ^1^ H spin–echo signal of (CH_2_) n and CH_3_ lipid groups in the range of 0–3.7 ppm. Diffusion decay was approximated by the sum of two exponentials in accordance with Equation (A1). The main part of the diffusion decay belongs to the tail, which is characterized by the self-diffusion coefficient *D_L_*, which is varied from 3∙10^−12^ m^2^/s to 10^−11^ m^2^/s depending on temperature and diffusion time.

**Figure 65:**
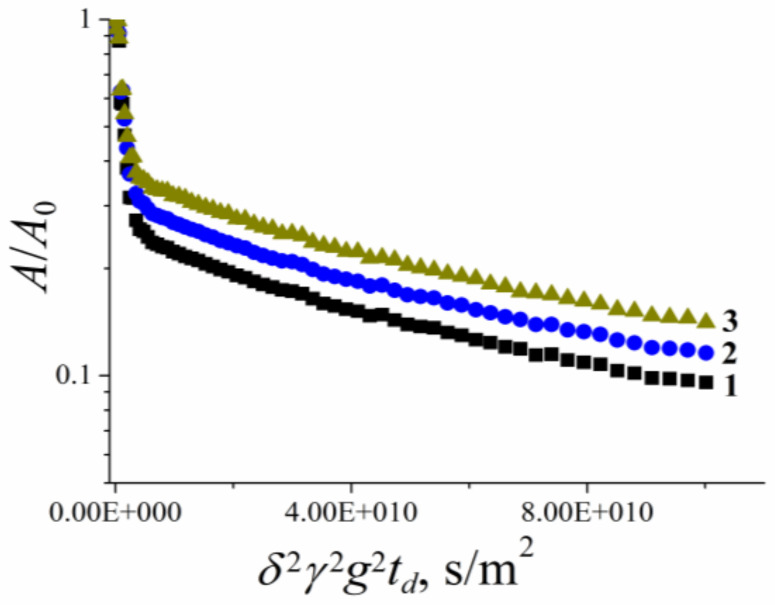
Diffusion decays of the ^1^ H spin echo–signal from different regions of integration: (1) 0–3.7 ppm, (2) 0–3 ppm, (3) 0–2 ppm. Diffusion time *t_d_* = 50 ms. Temperature is 35 °C.

Figure 65 shows the diffusion decays of ^1^ H spin–echo signals for different regions of integration. The relative part (population *p_L_*) of the smaller self-diffusion coefficient *D_L_* increases with the distance of the integration region of the spin–echo signal from the position of the water signal (Figure 65). This allows us to conclude that the self-diffusion coefficient *D_L_* characterizes the lateral diffusion of lipids. The components with large values of the self-diffusion coefficients, which determine the initial part of the diffusion decay, belong to the wings of the water signal.

**Figure 66:**
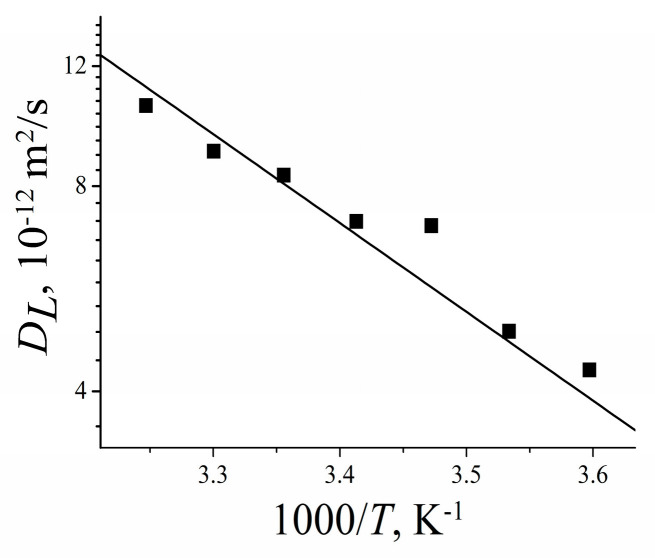
The temperature dependence of lipid self-diffusion coefficients *D_L_*. The straight line is the Arrhenius equation approximation. The activation energy is 25 ± 2.9 kJ/mol. The diffusion time *t_d_* is 10 ms. Reprinted with permission from [171]. Copyright 2016 Springer Nature.

The temperature dependence of *D_L_* obtained at a diffusion time *t_d_* = 10 ms in Arrhenius coordinates is shown in Figure 66. The activation energy of lateral diffusion of lipid molecules *E_D_* was 25 ± 2.9 kJ/mol. This value is in a good agreement with the activation energy of lipid diffusion in model systems (bilayer phospholipid membranes) *E_D_* = 25 kJ/mol [179].

**Figure 67:**
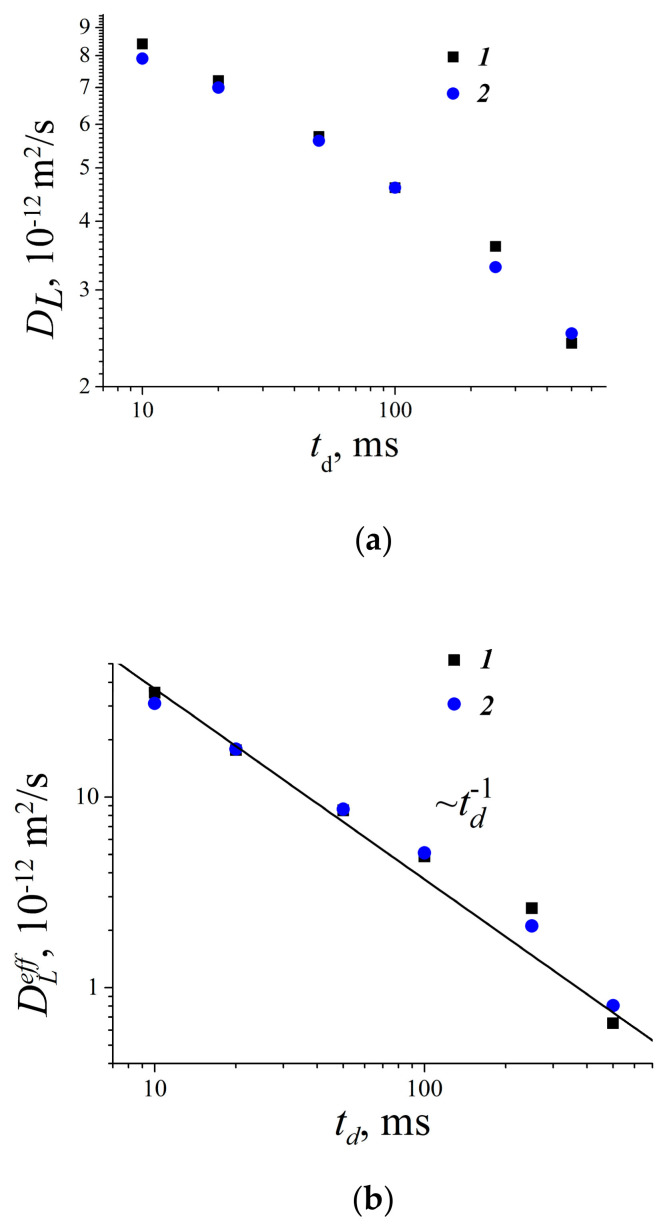
The dependence of the blood lipid self-diffusion coefficient *D_L_*(*t_d_*) on diffusion time *t_d_* at 20 °C (1) and 25 °C (2) (**a**). The dependence of effective blood lipid self-diffusion coefficient *D^eff^_L_*(*t_d_*) on diffusion time *t_d_* at 20 °C (1) and 25 °C (2), *D_p_* = 1.9·10^−12^ m^2^/s (**b**). Reprinted with permission from [171]. Copyright 2016 Springer Nature.

The lateral self-diffusion coefficient *D_L_* is decreased with increasing of diffusion time *t_d_*. The dependence *D_L_* (*t_d_*) is shown in Figure 67a. The effective self-diffusion coefficients *D_L_^eff^*, calculated from Equation (A5) is shown in Figure 67b. The estimated restriction size *a_L_* is about 1.4 μm.

#### 3.2.4. Self-Diffusion of Fullerene C_60_ Derivative Molecules in a RBCs Suspension

The study of self-diffusion of fullerene C_60_ derivative molecules in a suspension of erythrocytes was carried out [172,173]. Water-soluble fullerene derivatives (WSFD) are promising materials for biomedical research and further pharmacological applications [180,181,182]. We have registered and analyzed the diffusion decays of molecules of the following pentasubstituted derivatives of fullerene C60 in a RBCs suspension: with adducts of 3-mercaptopropanesulfonic acid C_60_[S(CH_2_)_3_SO_3_Na]_5_H (**1**), mercaptopropionic acid C_60_[S(CH_2_)_2_COOK]_5_H (**2**), proline C_60_[N(CH_2_)_3_CHCOOK]_5_Cl (**3**).

**Figure 68:**
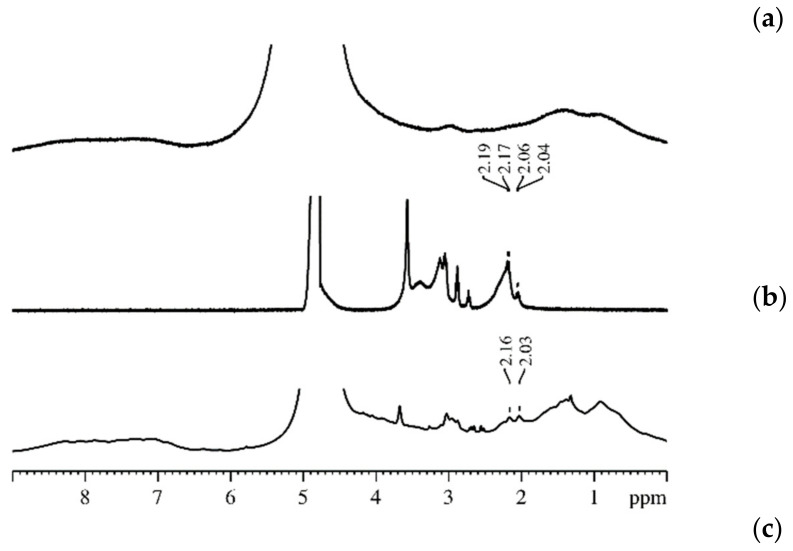
^1^ H spin–echo NMR spectra at low gradient amplitude pulses *g*: (**a**) RBCs suspension, *g* = 50 G/cm (0–1.70 ppm: (CH_2_)*n* and CH_3_ groups of lipids; 6–9 ppm: protein component (protons of aromatic rings and NH and OH groups)); (**b**) fullerene derivative **1** in aqueous solution, *g* = 55 G/cm (1.5–2.5 ppm: 10H, m, -CH_2_-CH_2_-CH_2_; 2.6–3.7 ppm: 20H, m, -CH_2_-S and -CH_2_-SO_3_Na); (**c**) RBCs suspension with added fullerene derivative **1**, *g* = 52 G/cm. The signals of the fullerene derivative components in suspension are indicated by vertical lines. Reprinted with permission from [172]. Copyright 2018 Elsevier.

**Figure 69:**
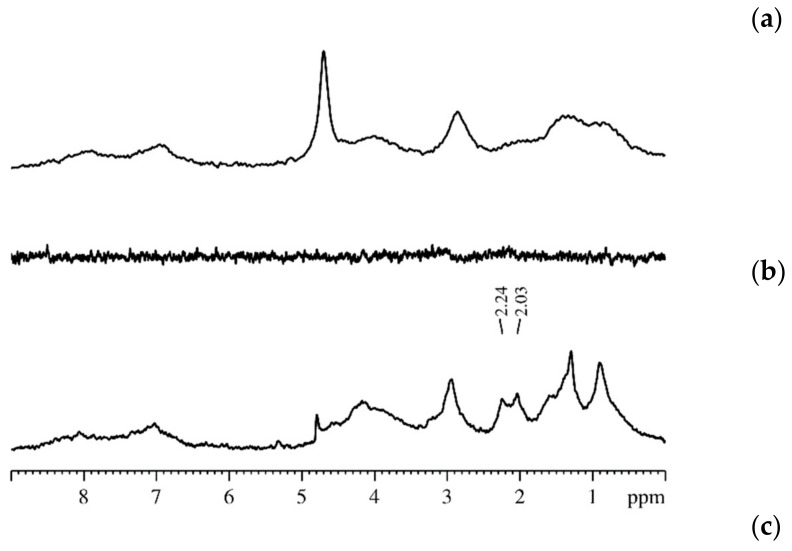
^1^ H spin–echo NMR spectra at high gradient amplitude pulses *g* = 700 G/cm: (**a**) RBCs suspension; (**b**) fullerene derivative **1** in aqueous solution; (**c**) RBCs suspension with added fullerene derivative **1**. The signals of the fullerene derivative components in suspension are indicated by vertical lines. Reprinted with permission from [172]. Copyright 2018 Elsevier.

Figure 68 and Figure 69 show examples of the spin-echo ^1^H NMR spectrum evolution in the pulsed field gradient experiment of RBCs suspension with and without addition of compound **1**. Figure 68 shows the echo spectra at a small value of the gradient pulse amplitude *g*. In these spectra, for all systems, intense signals of rapidly moving water molecules are observed; Figure 68b,c also show signals of molecules of fullerene derivatives in the aqueous phase. At large gradient amplitude, the NMR signals of rapidly moving water molecules and WSFD in the aqueous phase are suppressed, which is clearly observed in Figure 69b for an aqueous solution of WSDF. However, in Figure 69c, signals of slowly moving components of the RBCs-WSFD system are observed. This fact gives opportunity to record the tail in the diffusion decay of the WSFD and to estimate the corresponding self-diffusion coefficient. Similar results were also obtained for other systems. This observation indicates a relatively slow movement of WSFD molecules in a RBCs suspension compared to their diffusion in an aqueous solution. Therefore, we can assume that the translational mobility of WSFD molecules strongly depend on their interaction with RBCs.

**Figure 70:**
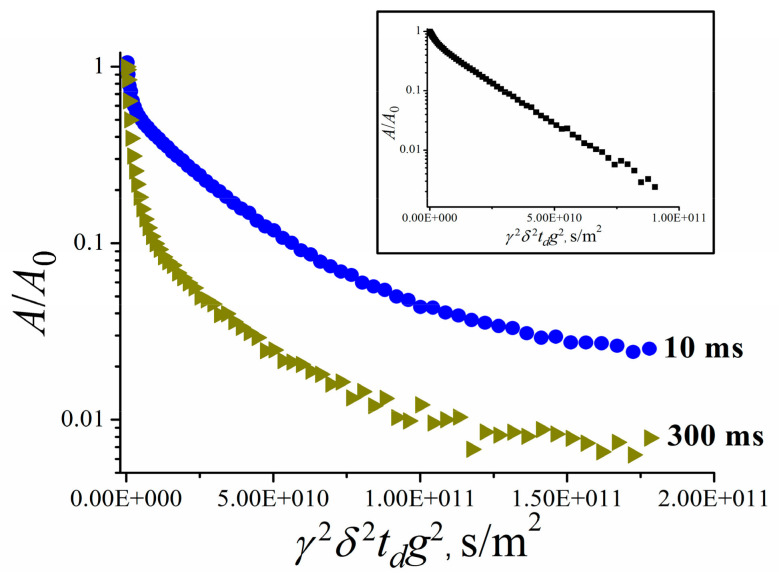
Diffusion decays at different diffusion times *t_d_* (indicated in the figure) of molecules **2** in RBCs suspension. Insert: diffusion decay of compound **2** in aqueous solution [173]. Reprinted with permission from [173]. Copyright 2021 Springer Nature.

In Figure 70, an example of diffusion decay of compound **2** molecules in RBCs suspension at diffusion time *t_d_* 10 and 300 ms is shown. The diffusion decay of compound **2** molecules in an aqueous solution is shown for comparison in insertion.

In aqueous solutions, diffusion decays of WSFD molecules are biexponential, with self-diffusion coefficients *D_s_*_1_^w^ and *D_s_*_2_^w^. At the same time, an additional third component appears in the RBCs suspension. Thus, the mobility of WSFD molecules in aqueous solutions is characterized by two self-diffusion coefficients *D_s_*_1_^w^ and *D_s_*_2_^w^, but in RBCs suspension—three diffusion components are observed *D_s_*_1_^s^, *D_s_*_2_^s^, and *D_s_*_3_^s^ (Table 12).

As it can be seen from Table 1, the self-diffusion coefficients *D_s_*_1_^w^, *D_s_*_2_^w^, and *D_s_*_1_^s^, *D_s_*_2_^s^ are close to each other in aqueous solutions and in a RBCs suspension. The larger value of the coefficients *D_s_*_1_^s^ in the suspension compared to aqueous solutions is apparently due to the partial overlap of the signals of protons of water molecules and the recorded signal, which, in turn, leads to an increase in the component *D_s_*_1_^s^. The presence of biexponentiality of diffusion decay in aqueous solutions of fullerene C_60_ derivatives is attributed to the formation of associates. In this case, the highest self-diffusion coefficient *D_s_*_1_^w^ corresponds to isolated molecules, and *D_s_*_2_^w^ to associated WSFD molecules.

As opposed to aqueous solutions in a RBCs suspension, the mobility of WSFD molecules is characterized by three coefficients of self-diffusion. The smallest coefficient *D_s_*_3_^s^ practically coincides with the coefficient of lateral diffusion of lipids in the erythrocyte membrane (≈7∙10^−12^ m^2^/s). The other two self-diffusion coefficients are close to the SDC of WSFD molecules in bulk aqueous solutions. Therefore, in RBCs suspension molecules of fullerene C_60_ derivatives are in an isolated and associated form in the aqueous phase, and are its also associated with the RBC membrane.

Due to the presence of permeability of RBCs membranes, there is an exchange between the WSFD molecules bound to the membrane and located in the aqueous phase. Quantitative information on metabolic processes can be obtained from an analysis of the dependence of the population *p_3_* (the relative fraction of the WSFD molecules in RBCs) on the diffusion time *t_d_*. Self-diffusion coefficients *D_s_*_3_^s^, population *p*_3_(0), and lifetimes *τ* of molecules of C_60_ fullerene derivatives in erythrocytes are presented in Table 13.

Thus, using the PFG NMR method, the ability of molecules of fullerene C_60_ derivatives to penetrate into biological membranes was revealed. Partial coefficients of self-diffusion of WSFD molecules in RBCs were determined. It was found that in a RBCs suspension WSFD molecules are in the form of isolated and associated molecules in the aqueous phase or are associated with the cell membrane. The time of exchange of WSFD molecules between the RBC membrane and an aqueous solution was estimated.

## 4. Summaries

The interaction of proteins with other macromolecules or small partner molecules plays an important role in most biological processes. NMR spectroscopy techniques in combination with pulsed magnetic field gradient NMR have a unique ability to extract information about these interactions and are used more often to investigate protein systems of increasing complexity, including proteins with an internally disordered structure. A characteristic feature of such proteins is that sample an ensemble of rapidly interconverting alternative conformations ranging from random coils to more structured conformations with secondary structure and residual tertiary structure elements. It should be recognized that the amount of experimental material concerning the transport properties of proteins in solutions and mixtures remains insufficient. As shown in a review of experimental results of studying the self-diffusion of polymers and globular proteins in solutions, NMR diffusion (PFG NMR) is one of the most effective methods for studying the structural and dynamic properties of molecules in condensed systems.

^1^ H pulsed field gradient NMR technique was shown to be a versatile tool for monitoring the penetration of water and biologically active species (fullerene derivatives) in RBC as well as their adsorption on the cellular membrane. The analysis of the spin echo attenuation curves during the pulsed field gradient ^1^H NMR experiment provides an opportunity to extract the partial self-diffusion coefficients and estimate the fractions of the water-soluble fullerene derivative molecules or clusters persistent in the aqueous phase or bound to the blood cells in the mouse erythrocyte suspension. It was revealed that fullerene derivative molecules most probably get fixed on the RBC surface (or inside the membrane) due to the fact that their self-diffusion coefficient is equal to the lateral diffusion coefficient of the cell lipids and close to fullerene derivative sorbed on RBC ghost and liposome self-diffusion coefficients. The average characteristic time reflecting the exchange of the fullerene molecules between the RBC-bound state and aqueous solution was estimated. Thus, the pulsed field gradient NMR can provide essential information projected on the in-vivo behavior of potential drug candidates after their intravenous administration in animals and humans.

## Figures and Tables

**Table 1:** Calculated from ^1^ H and ^7^ Li relaxation data (*D^calc^*) and experimentally measured (*D^exp^*) water molecule and lithium cation self-diffusion coefficients.

Amount of Water Molecules per One Sulfonate Group	*D*^calc^_H2O_ m^2^/s	*D*^exp^_H2O_ m^2^/s	*D*^calc^_Li+_ m^2^/s	*D*^exp^_Li+_ m^2^/s
4	5∙10^−12^	4∙10^−12^	2∙10^−12^	1∙10^−12^
20.5	3∙10^−10^	2∙10^−10^	4∙10^−11^	3∙10^−11^

**Table 2:** Hydration numbers of H^+^, Li^+^, Na^+^ and Cs^+^ cations in perfluorinated sulfonate (MF-4SC) and carboxylic (F-4CF) cation exchange membranes.

**Type of Membrane**	**MF-4SC**			
**Ionic Form**	**H^+^**	**Li^+^**	**Na^+^**	**Cs^+^**
*h* ± 0.5	2.3	3.8	3.5	1.7
**Type of membrane**	**F-4CF**			
**Ionic form**	**H^+^**	**Li^+^**	**Na^+^**	**Cs^+^**
*h* ± 0.5	1.3	2.8	2.0	1.0

**Table 3:** Hydration number *h* at different absolute amount of water molecules per ionic site SO_3_^−^ (*λ*) in acidic form Nafion 117 membrane [25].

*λ*, [H_2_O]/[SO_3_H]	Hydration Number *h*
1.9 ± 0.4	1.4 ± 0.5
3.2 ± 0.4	2.4 ± 0.5
4.4 ± 0.4	3.0 ± 0.3
5.8 ± 0.4	3.5 ± 0.3
6.4 ± 0.4	4.1 ± 0.3
7.4 ± 0.4	3.4 ± 0.3
12.0 ± 0.4	3.9 ± 0.3
17.5 ± 0.4	4.5 ± 0.5

**Table 4:** Hydration numbers *h* of Li^+^ cation in lithium form of Nafion 117 membrane at different water contents *λ* [109].

λ, [H_2_O/SO_3_^−^]	0.9	2.0	4.0	5.7	7.4	10.7	12
***H***	0.6 ± 0.3	1.2 ± 0.5	2.1 ± 0.5	2.6 ± 0.5	2.9 ± 0.5	4.2 ± 1.0	5.0 ± 1.0

**Table 5:** Crystallography radii, Stokes–Einstein hydrodynamic ion radii, hydration numbers (*h*) of Li^+^, Na^+^, and Cs^+^ cations in appropriate MSC membrane ionic forms at *RH* = 95% and in equimolar aqueous salt chloride solutions [17].

Cation	Li^+^	Na^+^	Cs^+^
Crystallography ionic radius, Å [30]	0.69	1.02	1.67
Stokes–Einstein hydrodynamic ionic radius, Å [31]	2.38	1.84	1.19
Stokes–Einstein hydrodynamic radius, estimated from ionic diffusion coefficient in chloride aqueous solution at infinite dilute concentration	2.70	2.20	1.50
Total water uptake of membrane (*λ*)	24.00	21.00	16.00
Water amount per membrane sulfonate group (*λ_s_*)	13.80	10.30	8.10
Hydration number of cations (*h*) in membrane	4.10 ± 1.00	5.00 ± 1.00	3.10 ± 1.00
Hydration number of cations (*h*) in aqueous solution [103,104]	4.00	4.60	3.90

**Table 6:** Ionic conductivities calculated based on diffusion coefficients of water and Li^+^ cations and measured for MF-4SC and F-4CF membranes in different ionic forms at the relative humidity of 90% [30].

Ionic Form	σ_MF-4SC_/S∙cm^−1^		σ_MF-4SC_/S∙cm^−1^	
	Experiment	Calculation	Experiment	Calculation
H^+^	2.8·10^−2^	4.3·10^−2^	1.5·10^−7^	1.5·10^−6^
Li^+^	6.2·10^−3^	1.1·10^−2^ (see ^a^)	1.0·10^−3^	4.3·10^−3^
		6.5·10^−3^ (see ^b^)		
Na^+^	6.1·10^−3^	1.2·10^−2^	1.2·10^−3^	4.6·10^−3^
Cs^+^	3.4·10^−4^	8.3·10^−4^	3.7·10^−4^	7.2·10^−4^

The calculations were carried out based on diffusion coefficients MF-4SC, F-4CF [20]: ^a^ water molecules and ^b^ lithium cations.

**Table 7:** Activation energies of Li^+^ and water molecule self-diffusion in Li^+^ ionic form of Nafion membrane *E_a_* at different water content *λ* [109].

*λ*, [H_2_O/SO_3_^−^]	0.9	2.0	4.0	5.7	7.4	10.7
***E_a_* Li^+^ cations self-diffusion, kJ/mol**	40.3 ± 2.0	38.4 ± 2.0	28.8 ± 2.0	25.0 ± 2.0	25 ± 2.0	27.8 ± 2.0
***E_a_* water molecules self-diffusion, kJ/mol**	-	28.8 ± 2.0	25.0 ± 2.0	23.0 ± 2.0	21.1 ± 2.0	19.2 ± 2.0

**Table 8:** Moisture content *λ*, amount water molecules per cation, self-diffusion coefficients *D_s_*, at 20 °C, self-diffusion activation energies *E_a_* of Li^+^, Na^+^, and Cs^+^ cations in Nafion membrane, MSC membrane at *RH* = 95% and chloride aqueous solutions [17,109].

Membrane Type	Cation	Moisture Content *λ*, Amount Water Molecules Per Cation	Cation Self-Diffusion Coefficient at 20 °C D_s_, m^2^/s	Cation Self-Diffusion Activation Energy *E_a_*, kJ/mol
Nafion	Li^+^	12	(1.5 ± 0.1)10^−10^	20.5 ± 1.0
	Na^+^	10	(2.1 ± 0.3)10^−10^	19.3 ± 1.5
	Cs^+^	4	(0.6 ± 0.2)10^−10^	24.8 ± 1.5
MSC [17]	Li^+^	24	3.7∙10^−10^	17.6
	Na^+^	21	4.4∙10^−10^	18.1
	Cs^+^	16	8.3∙10^−10^	16.5
Chloride aqueous solution	Li^+^	24	(8.2 ± 0.3)10^−10^	17.1 ± 0.5
	Na^+^	21	(1.1 ± 0.2)10^−9^	18.3 ± 0.6
	Cs^+^	16	(1.7 ± 0.2)10^−9^	16.8 ± 0.6

**Table 9:** Water content *λ*, hydration number *h*, self-diffusion coefficients *D_s_*, calculated conductivity *σ_c_*, measured conductivity *σ_e_* of Li^+^, Na^+^, Cs^+^ cations in Li^+^, Na^+^, Cs^+^ Nafion 117 membrane ionic forms, *RH* = 98%, *t* = 20 °C [109].

Ionic Form	Water Amount Per Sulfonated Group *λ*	Hydration Number h	Cation Self-Diffusion Coefficient *D_s_*, m^2^/s	Calculated Ionic Conductivity, *σ_c_* S/cm	Measured Ionic Conductivity, *σ_e_* S/cm
Li^+^	12	5.0 ± 1.0	(1.5 ± 0.1)10^−10^	(1.6 ± 0.1)10^−2^	(1.3 ± 0.1)10^−2^
Na^+^	10	6.0 ± 1.0	(2.0 ± 0.3)10^−10^	(2.0 ± 0.3)10^−2^	(1.1 ± 0.1)10^−2^
Cs^+^	4	1.0 ± 0.2	(0.6 ± 0.2)10^−10^	(6.0 ± 0.2)10^−3^	(2.3 ± 0.3)10^−3^

**Table 10:** Experimental values of ionic conductivity at 25 °C and conductivity activation energies of H^+^, Li^+^, Na^+^, Cs^+^ cations in MSC membrane with different humidity. Ion exchange capacity is 2.5 mg-eq/g [17].

*RH*, %	95		75		58		32	
Ionic Form	*E_a_*, kJ/mol	*σ_exp_* mS/cm	*E_a_*, kJ/mol	*σ_exp_* mS/cm	*E_a_*, kJ/mol	*σ_exp_* mS/cm	*E_a_*, kJ/mol	*σ_exp_* mS/cm
H	4.3	7.8	11	6.0	12	3.0	23	0.600
Li	7.5	1.9	30	0.5	39	0.2	52	0.008
Na	26.0	3.4	31	0.7	40	0.2	68	0.010
Cs	17.0	4.6	32	0.8	37	0.3	60	0.020

**Table 11:** Partial diffusion coefficients of water and ethanol and separation factor (*α*) of their mixture in membranes at 50 °C [48].

Membrane	*D*_H2O_·10^−10^/m^2^∙s^−1^	*D*_EtOH_·10^−10^/m^2^∙s^−1^	*α*
MF-4SC	6.0	1.500	48
PA-1	0.6	0.150	60
PA-2	40.0	0.100	10,000
PAA—PSF	1.3	0.026	800

**Table 12:** Self-diffusion coefficients of WSFD molecules in aqueous solutions and RBCs suspension.

Compound	Aqueous Solution		RBCs Suspension		
	*D_s_*_1_^w^·10^10^, m^2^/s	*D_s_*_2_^w^·10^11^, m^2^/s	*D_s_*_1_^s^·10^10^, m^2^/s	*D_s_*_2_^s^·10^11^, m^2^/s	*D_s_*_3_^s^·10^12^, m^2^/s
**1**	4.1 ± 0.4	7.4 ± 0.7	5.5 ± 0.8	3.9 ± 0.6	5.5 ± 0.8
**2**	4.3 ± 0.8	7.5 ± 1.5	7.1 ± 1.4	4.4 ± 0.9	5.0 ± 1.0
**3**	1.2 ± 0.1	4.9 ± 0.5	8.0 ± 1.0	3.8 ± 0.6	6.0 ± 1.0

**Table 13:** The self-diffusion coefficients *D_s_*_3_^s^, population *p*_3_, and lifetimes *τ* of WSFD molecules in RBCs [173].

Compound	*D_s_*_3_^s^·10^12^, m^2^/s	*p*_3_(0)	*τ*, ms
**1**	5.5 ± 0.8	0.33	440 ± 70
**2**	5.0 ± 1.0	0.13	470 ± 70
**3**	6.0 ± 1.0	0.06	1200 ± 300

**Figure A1:**
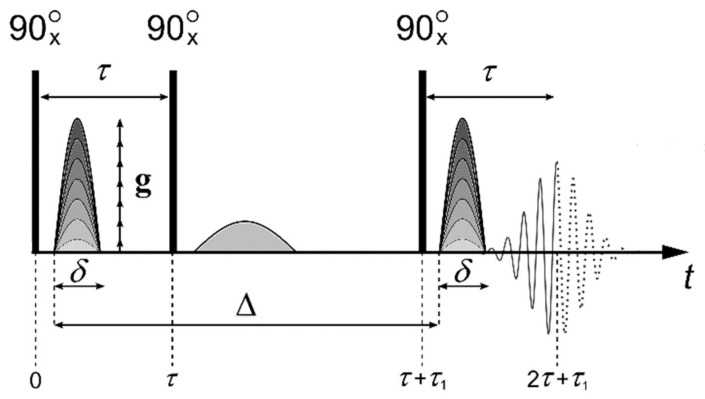
Stimulated echo pulse sequence with the magnetic field gradient pulses.

## Appendix A

### Pulsed Field Gradient NMR Technique

In our review we will discuss the results of ion-exchange membranes and biological systems investigation by hetero nuclear high resolution NMR, NMR relaxation and pulsed field gradient NMR. High resolution and spin relaxation NMR techniques are well known and describe elsewhere. Pulsed field gradient NMR is not so widely applied; therefore, in our paper we will give the brief introduction in this technique. Pulsed field gradient NMR is attractive for studying diffusion because it is suitable for direct measurement of the diffusion coefficients and the relative fractions of the diffusant in different phases of heterogeneous systems. As a rule, the diffusion coefficients are measured by stimulated echo sequences [42,43].

The stimulated spin echo signal involves three radio- frequency 90° (RF) pulses (Figure A1). Magnetic field gradients are formed between the first and the second and after the third RF pulse. Under these conditions, the translational displacement of a species is accompanied by irreversible dephasing of the magnetization vector, which results in a decrease in the spin echo signal amplitude. Conventionally, the dependence of the echo signal intensity *A* on the magnetic field gradient amplitude *g* called the “diffusion decay” *A*(*g*) is analyzed. For slow exchange of molecules occurs between the phases, the diffusion time *t_d_ = ∆−δ/3* much more compared to the diffusant lifetime in *i-th* phase diffusion decay is approximated by Equation (A1).

Here, *τ* is the time interval between the first and second RF pulses, *τ*_1_ is the time interval between the second and the third ones, ∆ is the interval between the gradient pulses, *δ* is duration of the equivalent rectangular magnetic field gradient pulses, *g* is the amplitude of the magnetic field gradient pulse.

$$A\left(g\right)=\frac{A\left(2\tau ,\tau_{1},g\right)}{A\left(2\tau ,\tau_{1},0\right)}=\overset{m}{\underset{i=1}{\sum }}p_{i}^{\prime }\;exp\;(-\gamma^{2}g^{2}\delta^{2}t_{d}D_{si})$$

(A1) $$p_{i}^{\prime }=p_{i}\;exp\;\left(-\frac{2\tau }{T_{2i}}-\frac{\tau_{1}}{T_{1i}}\right)/\overset{m}{\underset{i=1}{\sum }}p_{i}\;exp\;\left(-\frac{2\tau }{T_{2i}}-\frac{\tau_{i}}{T_{1i}}\right)$$

$$\overset{m}{\underset{i=1}{\sum }}p_{i}=1$$

where *γ* is the gyromagnetic ratio, *t_d_* is *∆−δ/3, D_si_* is the diffusion coefficient in the *i-th* domain (phase), *p_i_* is the relative fraction of molecules in the *i-th* domain (population of the *i-th* phase), *T_1i_, T_2i_* are the times of longitudinal and transverse relaxation times of nuclei in the *i-th* phase.

For the molecules undergoing unhindered isotropic Brownian motion, the evolution of spin echo signal is described by the following exponential Equation.

(A2) $$A\left(2\tau ,\tau_{1},g\right)=A\left(2\tau ,\tau_{1},0\right)\;\exp\;\left(-\gamma^{2}g^{2}\delta^{2}t_{d}D_{s}\right)$$

where *γ* is gyromagnetic ratio, *t_d_*=Δ-*δ*/3 is the diffusion time, and *D_s_* is the self-diffusion coefficient, *τ*, *τ*_1_ and *g* are shown in Figure A1; *A* (2*τ*, *τ*_1_, 0) is expressed by the Equation:

(A3) $$A\left(2\tau ,\tau_{1},0\right)=\frac{A\left(0\right)}{2\;exp\;\left(-\frac{2\tau }{T_{2}}-\frac{\tau_{1}}{T_{1}}\right)}$$

where *A*(0) is the signal intensity after the first radio frequency (RF) pulse (Figure A1). *T*_1_ and *T*_2_ are the spin–lattice and spin–spin relaxation times, respectively. During measurement of echo signal evolution, *τ* and *τ*_1_ are fixed, and only the dependence of *A* on *g* is analyzed.

By analyzing the *A*(*g*) dependence under slow exchange conditions, it is possible to determine the partial diffusion coefficients and the relative proportions of the diffusant molecules in various nano- and microvolumes of membrane systems.

The procedure for calculation of diffusion coefficients in complex polymer electrolytes was described in several publications [48,50]. The modern NMR spectroscopic instrumentation allows the diffusion coefficient to be measured on various spatial scales starting from several tenths of micrometer; the measurable diffusion coefficients vary from 10^−14^ to 10^−8^ m^2^/s.

Pulsed field gradient NMR technique application requires sufficiently long (several milliseconds and more) spin-spin relaxation times. This complicates the measurements of diffusion coefficients of ions with quadrupole nuclei (e.g., ^23^ Na, ^133^ Cs). The majority of studies were carried out on the nuclei characterized by sufficiently long transverse relaxation times (^1^ H, ^7^ Li, ^19^ F).

Modern NMR spectrometers give possibility to record a Fourier transform spin echo spectra, therefore, we can measure partial self-diffusion coefficients of different molecules or molecule fragments. Note that, as in the case of relaxation NMR experiments, applying of the Fourier transform of the stimulated echo, and thus acquiring the high-resolution NMR spectrum for investigated molecules significantly simplifies interpretation of the diffusion data particularly in terms of corresponding data to different lines in spectrum therefore, to the different molecules.

The uniqueness of the pulsed field gradient NMR method lies in the possibility of studying the geometry and permeability of porous media and biological cells. Its idea is based on analysis of how the diffusion decay curve depends on the diffusion time.

In Figure A2 idealized S-shape dependence of the self-diffusion coefficient *D_s_* of diffusant molecules on the diffusion time *t_d_* in porous systems with permeable walls is shown [82,83,174,175,183,184,185]. It is known from the literature that the dependence of the SDC of water molecules on the diffusion time in porous systems and biological cells is S-shaped, as shown in Figure A2.

At short diffusion times *t_d_* (region free diffusion), the diffusant molecules are not able to reach the pore walls so a self-diffusion coefficient does not depend on *t_d_* and *D_s_* = *D*_0_, where *D*_0_ is the self-diffusion coefficient of a bulk liquid.

In the region of restricted diffusion diffusant, molecules are collided with the pore wall and *D_s_* depend on *t_d_*, becoming a decreasing function of time.

At long diffusion time the diffusant molecules as a result of the permeation effect to diffuse into adjacent pores. Their movement is averaged over the entire pore space, the measured diffusion coefficient *D_s_ = D_p_* also does not depend on *t_d_* (region of hindered diffusion), and *D_p_* value is less than *D*_0_.

If self-diffusion is observed in a completely isolated pore, then the mean square displacement of molecules is limited by the size of the pore. Therefore, with the increasing of diffusion time *D_s_* decreases as *t_d_^−1^* and in the limit *t_d_*→∞, *D_s_*→0. From the Einstein Equation (A4) pore size *a* may be calculated.

(A4) $$D_{s}\left(t_{d}\right)=\frac{a^{2}}{6t_{d}}$$

The permeability may be estimated from Equation (A5) [174]:

(A5) $$\frac{1}{D_{p}}=\frac{1}{D_{0}}+\frac{1}{p_{a}}$$

Biological cells are permeable therefore in restriction region *D_s_*(*t_d_*)∝*t_d_*^−n^, where *n*< 1. For quantitative determination of the limitation size and permeability, for permeable pores the scaling approach which is described in several papers was applied [82,83].

In this case, the effective self-diffusion coefficient *D_s_^ef^*^f^ is calculated in which dependence on the diffusion time *t_d_* will be∝*t_d_*^−1^:

(A6) $$D_{s}^{eff}\left(t_{d}\right)=\frac{D_{s}\left(t_{d}\right)-D_{p}}{D_{0}-D_{s}\left(t_{d}\right)}\cdot D_{0}$$

where *D*_0_ is the self-diffusion coefficient at *t_d_*→0 (not restriction region), *D_s_(t_d_)* is the experimentally obtained dependence, *D_p_* is a hindered self-diffusion coefficient (*t_d_*→∞).
